# Barley Seeds miRNome Stability during Long-Term Storage and Aging

**DOI:** 10.3390/ijms22094315

**Published:** 2021-04-21

**Authors:** Marta Puchta, Jolanta Groszyk, Magdalena Małecka, Marek D. Koter, Maciej Niedzielski, Monika Rakoczy-Trojanowska, Maja Boczkowska

**Affiliations:** 1 National Centre for Plant Genetic Resources, Plant Breeding and Acclimatization Institute (IHAR)—National Research Institute, 05-870 Radzików, Poland; j.groszyk@ihar.edu.pl (J.G.); m.malecka@ihar.edu.pl (M.M.); 2 Department of Plant Genetics, Breeding, and Biotechnology, Warsaw University of Life Sciences, 02-787 Warsaw, Poland; marek_koter@sggw.edu.pl (M.D.K.); monika_rakoczy_trojanowska@sggw.edu.pl (M.R.-T.); 3 Department of Plant Conservation Biology, Polish Academy of Sciences Botanical Garden—Center for Biological Diversity Conservation in Powsin, 02-973 Warszawa, Poland; mniedz@obpan.pl

**Keywords:** barley, long-term storage, next generation sequencing, miRNA, seed aging

## Abstract

Seed aging is a complex biological process that has been attracting scientists’ attention for many years. High-throughput small RNA sequencing was applied to examine microRNAs contribution in barley seeds senescence. Unique samples of seeds that, despite having the same genetic makeup, differed in viability after over 45 years of storage in a dry state were investigated. In total, 61 known and 81 novel miRNA were identified in dry seeds. The highest level of expression was found in four conserved miRNA families, i.e., miR159, miR156, miR166, and miR168. However, the most astonishing result was the lack of significant differences in the level of almost all miRNAs in seed samples with significantly different viability. This result reveals that miRNAs in dry seeds are extremely stable. This is also the first identified RNA fraction that is not deteriorating along with the loss of seed viability. Moreover, the novel miRNA hvu-new41, with higher expression in seeds with the lowest viability as detected by RT-qPCR, has the potential to become an indicator of the decreasing viability of seeds during storage in a dry state.

## 1. Introduction

The aging process does not discriminate against any living organism. It begins at birth, leads to inevitable death, and is based on retrograde cellular changes occurring during the lifetime [1]. Life expectancy is a feature that is highly differentiated among living organisms. It is worth noting that it is significantly prolonged in plants in relation to animals, e.g., such plants as *Pinus aristata* Engelm. in White Mountain, California, which is about 5000 years old [2]. For most species, plants at the seed stage are able to survive extremely long, e.g., *Nelumbo nucifera Gaertn.* seeds dating back to around 1300 years old with a germination capacity of 84% [3]. Due to environmental degradation, intensive plant species conservation is required. Seeds that have been developed evolutionary for generative reproduction and as spore organs constitute the vast majority of germplasm stored in Gene Banks. They are preserved there in case of the species extinction in the natural environment. However, the fundamental problem is molecular changes associated with the natural aging process, reducing seed viability and vigor.

In the last decade, intensive scientific research has been conducted to identify changes in nucleic acids, proteins, and metabolites during seed dormancy. This omnibus approach has not yet led to important, innovative insights, but has enriched the understanding of the dormancy and germination process. In addition, the resulting data will be a valuable background for further studies. Despite the progress made in recent years, some important questions remain unanswered. The molecular characterization of seeds aging, dormancy, and germination requires continuing research to understand these processes [4]. Among the factors that can play a key role are small non-coding RNAs.

Small RNAs (sRNAs) include several short non-coding RNAs groups: microRNA (miRNA) and small interfering RNA (siRNA), which regulate gene expression at the posttranscriptional level. Variable sRNA levels in plant cells, suggests their regulatory role. The best-characterized class of plant sRNA is miRNA [5]. They are 19–24 nucleotides non-coding endogenous RNAs [6] that play an important role in regulating gene expression at posttranscriptional level, resulting in mRNA cleavage or translation inhibition of target transcripts.

It is estimated that genes coding miRNA represent 1% of predicted genes, in higher eukaryotic genomes and the expression of 10–30% of genes can be regulated by them [7]. After the discovery of *lin-4*, the first miRNA in *Caenorhabditis elegans*, different miRNA has been identified in different species of living organisms including plants [7]. The role of miRNAs in plants has been illustrated by regulating processes responsible for root, leaf, shoots, and flowers development. Additionally, they also take part in response to phytohormones, nutrients, and environmental stresses, e.g., the target genes for miR393 are auxin receptors. Together with siRNAs, they are essential for cell development, differentiation, morphogenesis, signal transduction, and have also showed their involvement in adaptive reactions to various biotic and abiotic stresses [7]. miRNAs are also induced by pathogens, which indicates their involvement in interactions between plants and microorganisms [5]. In addition, the studies have shown that species-specific and conserved miRNAs play an important role in shaping morphologies variability, developmental variability, and resistance to abiotic and non-abiotic stress [8,9,10].

Currently, 14 197 mature miRNAs from 82 plant species have been discovered and deposited in a public database, i.e., miRBase [11]. So far, there are 325 miRNAs identified in maize, 738 in rice, and 428 in *Arabidopsis thaliana* L. [11]. Identifying of a complete set of miRNAs is fundamental to understanding the regulation of small RNAs and their diversity [12].

Although small RNAs have been involved in many developmental processes, their role in the seed germination phases is poorly known. Das et al. [6] suggested that the dynamic change in the expression of several miRNAs, their targets, and the interference during signal transmission between miRNAs and ta-siRNAs (trans-acting siRNA) contribute to the regulation of seed germination in *A. thaliana*. Studies have indicated numerous miRNAs involved in the dynamic process of seed germination. However, little is known about specific miRNA regulations and their targets, which are potentially important factors contributing to the early stages of seed germination [13]. The studies on small RNAs and their targets are intended to provide information to further understand the regulatory network managed by miRNAs for seed germination.

*Hordeum vulgare* L. is one of the most important cereals, which ranks fourth within the world’s cereal crops [14,15]. It is used as animal feed, human food, and in brewing [16]. Due to the known genome and the significant interest of researchers around the world, which resulted in an extensive amount of information, barley qualifies as a model plant for research into tribe *Triticeae*. The haploid barley genome contains 83,105 putative genetic loci including 39,734 high-confidence ones. It is among the largest diploid genomes sequenced to date [17]. However, barley miRNAs sequence identification has so far been limited to only four tissues, i.e., stem, leaves, ears, and roots at different stages of development (seedlings, tillering, shading, grain filling) using Illumina sequencing technology. In their research, Lv et al. [7] identified barley miRNAs to produce barley miRNA profiles that will shed more light on their roles in biological function and evolution.

The principal aim of the study is the profiling of miRNA in dry barley seed after long term storage and its role in determining and regulating seed viability. 

## 2. Results

### 2.1. Overview of Small RNA Library Sequencing

In order to explain the role of miRNA in the seeds aging process, nine miRNA libraries were constructed. 

A total of 28,019,900 reads from the first run and 25,796,390 reads from the second run were obtained. After filtering the reads in terms of quality and length (<17 bp and >25 bp), there were obtained on average: 815,627 reads for renewed seeds (Rc- regenerated in 2017/2018 season), 480,785 for seeds with low viability (Lv, low viability; 2% of germability after storage 1972–2018), and 870,166 for highly viable seeds (Hv, high viability; 86.7% of germability after storage 1972–2018). Over 40% were mapped to the *H. vulgare* reference genome, of which about 10% were rRNA and tRNA (Table 1). 

**Figure 1:**
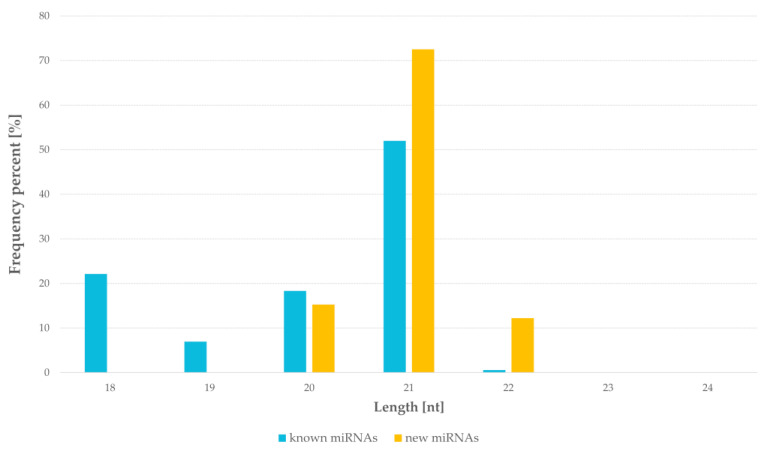
Size distribution of miRNAs in results of next generation sequencing of barley seeds.

In miRNAs libraries, the lengths of the miRNAs sequences were 18–22 nucleotides. The most frequent was 21 nucleotides fraction and it represented 52% of known miRNAs and 72% of novel miRNAs. The 18 nucleotides fraction was 22% of known miRNAs and 20 nucleotides was 15% of novel miRNAs. The smallest fraction of miRNAs was 22 nucleotides for both groups. Neither known nor novel miRNAs of length 23 nt and 24 nt were identified in the studied samples (Figure 1).

### 2.2. Identification of Known and Novel miRNAs

**Figure 2:**
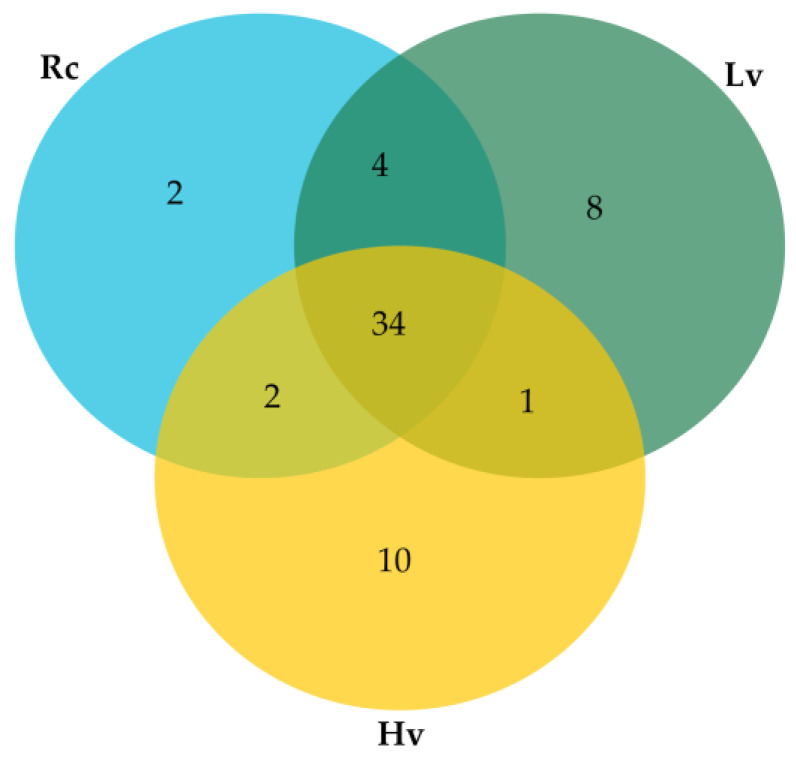
Venn Diagram for number of known miRNAs detected in barley dry seeds. Rc—renewed seeds sample; Lv—low viable seeds after long-term storage in a dry state; Hv—highly viable seeds after long-term storage in a dry state.

Briefly, 34 known miRNAs occurred in all samples, two miRNAs unique to renewed seeds (Rc) sample, eight miRNAs unique to low viable seeds (Lv), and ten miRNAs unique to highly viable seeds (Hv) samples were identified (Supplementary Table S1). Four common miRNAs were identified for Rc samples and Lv samples, one miRNA for Hv and Lv samples, and two miRNAs for Rc and Hv samples (Figure 2).

**Figure 3:**
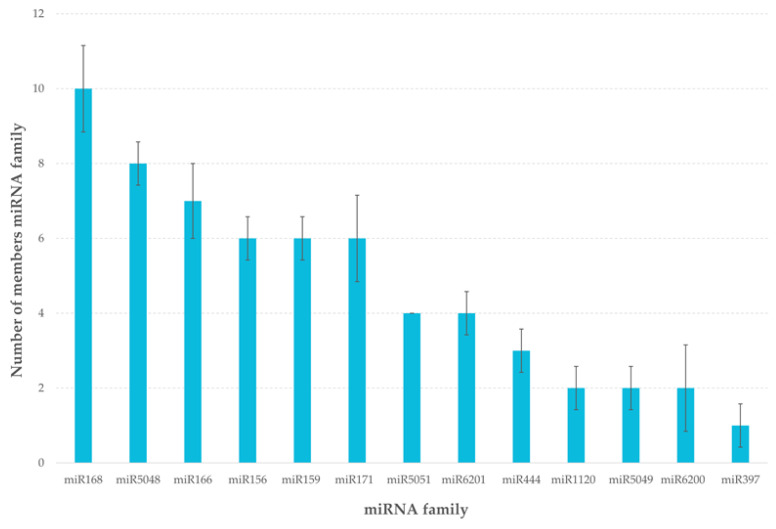
Number of isomiRs in miRNA family based on results of sRNA-Seq analysis of barley seeds.

The most numerous group was the 168 miRNA family containing ten different isomiRs and the miR5048 and miR166 families containing eight and seven isomiRs, respectively. As isomiRs, variants of mature miRNAs differing from their reference sequences in the miRBase [11] were considered. The variation results from 5′- or 3′-trimming variants arising during miRNA biogenesis or during pri-miRNA post-trancriptional editing [18,19]. The smallest family was miR397 containing only one member (Figure 3). 

Canonical mature miRNAs represented slightly over 50% of all identified known miRNAs. The remaining fraction consisted mainly of non-coincidental 3′trimmed isomiRs (average 47%), 5′trimmed isomiRs (about 0.2%) and two-side trimmed (about 0.5%). 0.1% of known miRNAs with very low frequencies were considered to be random isomiRs. For three families, i.e., miR1120, miR168, and miR6200, the canonical forms with a length of 24 nt (miR1120 and miR168) and 22 nt (miR6200) were not identified in the examined samples. The occurring canonical miRNAs were mostly 21 nt in length. Only in the case of the miR6201 family were there molecules with a length of 22 nt.

**Figure 4:**
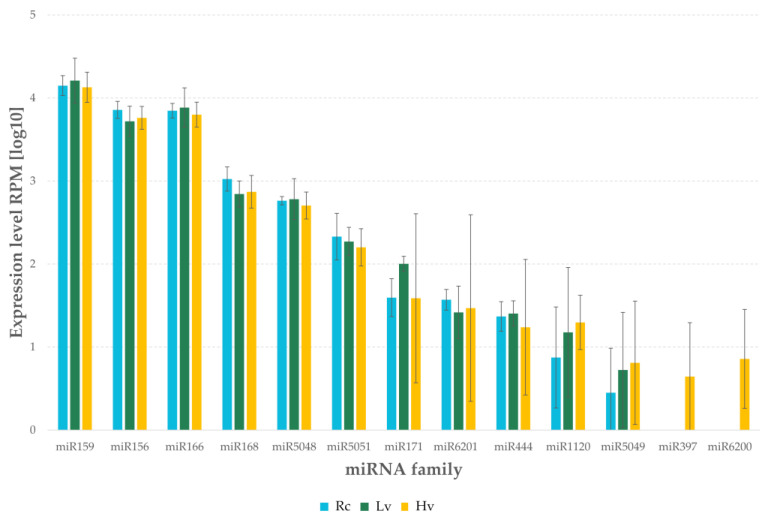
Expression level of miRNAs by sRNA-Seq barley dry seeds. Rc—renewed seeds sample; Lv—low viable seeds after long-term storage in a dry state; Hv—highly viable seeds seeds after long-term storage in a dry state.

The highest level of expression was observed for the family miR159 (Figure 4). The level of expression was 48,560.66 RPM in Lv sample, 42,273.78 RPM in Rc sample and 40,435.63 RPM in Hv sample. A comparably high level of expression was observed in miR156 and miR166 families, where the level of expression was over 15,000 RPM for all of the samples. The highest level of the miR156 family expression was observed in Rc sample, whereas the miR166 family in Lv sample. The level of expression above 10,000 RPM was observed for the miR168 and miR5048 families in all samples. However, both families had the lowest expression level in Lv sample. For the families, miR387 and miR6200 expression was observed only in the Hv sample (Supplementary Table S1).

**Figure 5:**
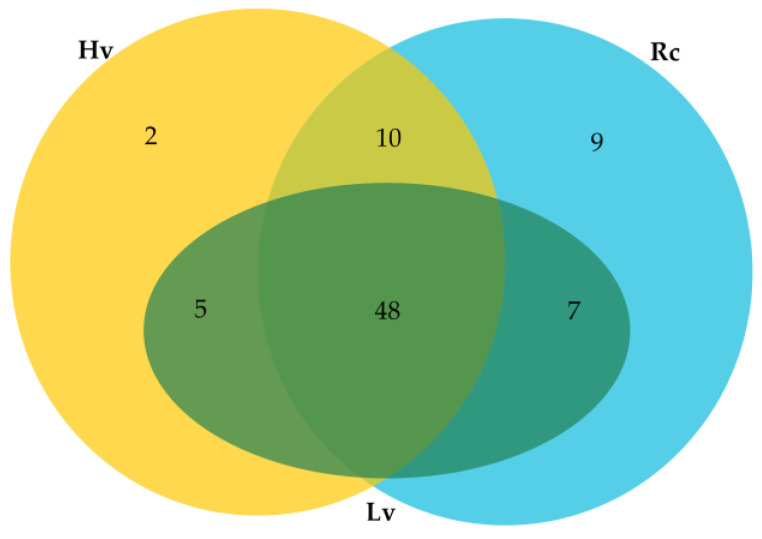
Venn diagram for number of novel miRNAs detected in barley dry seeds. Rc—renewed seeds sample; Lv—low viable seeds after long-term storage in a dry state; Hv—highly viable seeds after long-term storage in a dry state.

In addition, 81 novel miRNAs were identified, of which 48 miRNA were found in all the samples. Two miRNAs unique to Hv sample and nine miRNAs unique to Rc sample were identified. Moreover, ten novel miRNAs common to Rc and Hv samples, seven to Rc and Lv samples, and five to Lv and Hv samples were found (Figure 5, Supplementary Table S2). Overall, the level of expression of the novel miRNAs was much lower than the known ones. The highest expression had hvu-new80. As many as 70 novel miRNAs had an expression level below 100 reads per million (RPM) (Supplementary Table S2).

Among the novel miRNAs, eighteen miRNAs were found with a mature sequence matching in the miRBase [11]. Some novel miRNAs are isomiRs in already known families: thirteen novel miRNAs were assigned to known families, one novel miRNA per miR159, miR166, miR168, miR156, miR396, miR171, and miR397, and five to miR167 family (Table 2). The secondary structures of miRNA hairpin precursors were predicted according to the criteria reported by Axtell and Meyers [20] (Supplementary Table S3; Supplementary Figure S1).

### 2.3. Differential Expression of miRNAs 

The differential expression (DE) analysis was performed to compare the level of miRNA in barley seeds with different viability and storage time. It based on the normalized read counts for each identified miRNA. Samples after long-term storage having different viability (Hv and Lv) did not show significant differences in the miRNA expression level. Only two miRNAs significantly differentiated the regenerated seed (Rc) sample from the long-term stored samples (Hv and Lv). Both of them were novel miRNAs. Hvu-new60 was present only in Rc sample and hvu-new2 was present only in samples after long-term storage (Supplementary Table S4).

**Figure 6:**
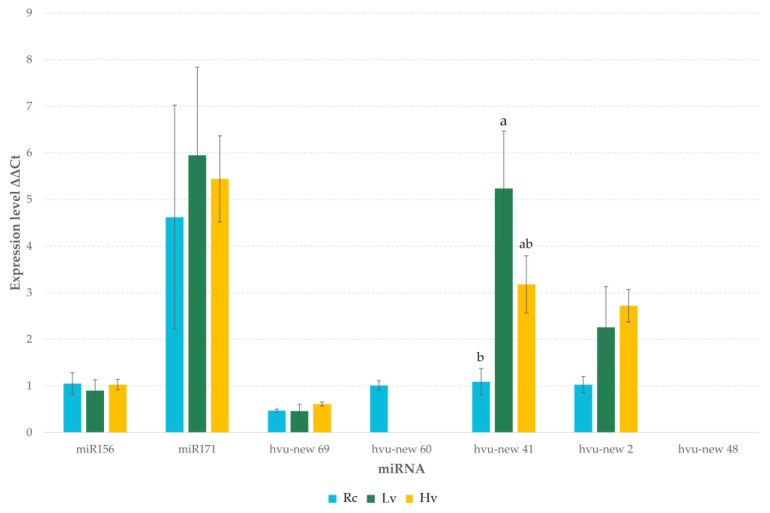
Expression level of selected miRNA by RT-qPCR in barley dry seeds. Rc—renewed seeds sample; Lv—low viable seeds after long-term storage in a dry state; Hv—highly viable seeds after long-term storage in a dry state. Letters indicate homogeneous groups based on the Tukey’s HSD test.

The expression levels of some novel and known miRNAs were verified using RT-qPCR methods (Figure 6). A similar expression profile was observed for the majority of miRNAs in the tested sample. The hvu-new48 failed to amplify. However, this novel miRNA was found in a very small number in NGS sequencing results. As in DE analysis, the results of RT-qPCR did not indicate the presence of significant differences in the level of tested miRNAs in seed samples with different viability and storage duration. However, ANOVA analysis of RT-qPCR results showed significant differences in hvu-new41 expression levels. The Tukey’s honest significant difference test showed that the level of the miRNA is significantly higher in Lv sample than in Rc, while Hv sample had an intermediate level.

### 2.4. Prediction Targets of miRNA

The analysis of the targets functions was performed for all miRNAs. Target predictions indicated more than one target for each miRNA. In total, 11,771 potential targets were identified for the known and novel miRNAs, of which only 856 originated from the barley genome (Supplementary Table S5). The most suitable targets identified in barley genome were TC240039 (UniRef A4HRC2) encoding NAC transcription factor (expectation 1) for hvu-new63 and TC253359 (UniRef Q40073) encoding Ribulose bisphosphate carboxylase/oxygenase activase A for miR1120 (expectation 3). The novel miRNA hvu-new60 had an affinity for thirteen potential targets with five encoding proteins, while hvu-new2 had eleven potential targets and encoding proteins. Four proteins were encoded by seven identified targets for miRNA hvu-new41 (Table 3). Approximately 15% of the potential targets in the barley genome for both new and known miRNAs had the translational inhibition suggested as a possible process of miRNA-RISC mediated gene inhibition. The translational inhibition potential was reported by psRNATarget [21] whenever a mismatch was found in the central complementary region of the miRNA sequence.

**Figure 7:**
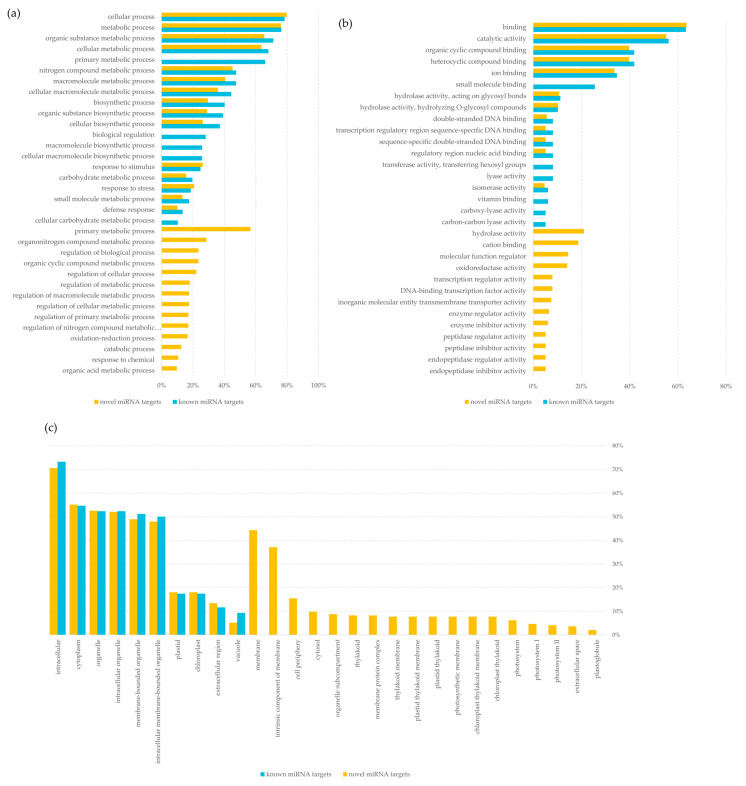
Analysis of target function using g:Profiler toolset [22] for known and novel miRNA detected in barley seeds, (**a**) biological process; (**b**) molecular function; (**c**) cellular anatomical entity.

Furthermore, all the potential targets of miRNAs were subjected to Gene Ontology (GO) analysis to determine their GO terms, which could provide insight into the miRNAs function. The GO analysis showed that the highest number of putative targets (over 75%) was involved in cellular and metabolic processes. Potential targets for novel miRNAs were involved in 114 biological processes and those for known miRNAs only in 38. Interestingly, 57% of potential targets for novel miRNAs were involved in primary metabolic processes. No targets for known miRNAs were assigned to this category (Figure 7a, Supplementary Table S6). The most common group among identified potential targets were those related to the molecular functions involved in binding and catalytic activity (over 55%). Similarly to biological processes, the putative targets for the novel miRNAs represented more groups of molecular functions, although the difference was not so significant (46 for the novel miRNAs and 32 for known miRNAs). Uniquely 21% of the potential targets for the novel miRNAs had a molecular function related to hydrolase activity. In contrast, 26% of the selected targets for known miRNAs performed a small molecule binding function. None of the targets identified for novel miRNAs had such a function (Figure 7b, Supplementary Table S6). In turn, the most common cellular anatomical entities associated with potential targets for both known and novel miRNAs were intracellular, cytoplasm, and organelle (over 70%, 55%, and 50%, respectively). Among the unique potential targets for the novel miRNAs were those associated with chloroplast internal structures, i.e., thylakoid and its membranes and photosystem (Figure 7c, Supplementary Table S6).

## 3. Discussion

Small non-coding RNAs, including miRNAs, play a key role in plant ontogenesis [23]. More and more research on the role of miRNAs in seed development and germination is being conducted [5,24,25,26,27]. However, miRNome in dry seeds with quiescent metabolism has been relatively rarely analyzed [25,28,29]. So far, the effect of long-term seed storage and the inextricably linked ageing on miRNA in barley seeds has not been studied. 

Here, the unique plant material was used. Two samples of barley seeds exposed to long-term storage in a dry state were used. Their uniqueness results from the origin of a single seed lot. This guarantees no differences resulting from environmental conditions during ripening, post-harvest treatment and preparation for storage. Therefore, it can be assumed that, at the beginning of storage, they had not only homogeneous genetic background but also homogeneous transcriptome and miRNome. During long-term storage, the moisture content of the seeds in one flask increased probably due to its slight leakage. This resulted in a decrease in seed viability. The significant difference in the viability of the two seed samples made them a perfect material for studying the effects of aging at a molecular level. The studies presented here were aimed to determine the relationship between the aging process of seeds and miRNome.

As a result of high-throughput sequencing in the presented study, 61 known miRNAs were identified that were matched to 13 families available for barley at miRBase ver. 22.1 [11]. Also, 81 novel miRNAs, of which 13 were matched as new isomiRs for barley miRNAs, were found. 

### 3.1. miRNA and Seed Aging

Seed aging is associated with changes in macromolecules such as proteins, lipids and nucleic acid [30,31,32]. These changes are most likely caused by reactive oxygen species (ROS) [33,34,35,36]. In dry seeds a metabolism is quiescent because of the low water content, the viscosity increases, and the glassy state forms and the chemical reactions are reduced [37,38]. Aging process progressively occurs due to ROS origin damage. ROS are formed as a result of non-enzymatic reactions such as lipid peroxidation or Maillard reactions [39,40,41]. Compared to DNA, which is double stranded, bound to chromatin and located in the nucleus, RNA undergoes damage easily. mRNA is susceptible to breakage and nucleotides can be removed or modified [42]. Dry, mature seeds contain various long-lived mRNA, which are present in seeds from late embryogenesis to early seed germination and are crucial for protein synthesis during the early phase of germination [43,44]. The relationship between RNA fragmentation and reduced viability has been demonstrated in soybean seeds and the damages occur randomly throughout the transcriptome [45,46]. Random RNA threads breakage occurred also during the aging of *Arabidopsis* seeds [47]. Dry seeds also contain long-lived miRNAs, which act as a regulator of gene expression during germination [28,48,49]. Therefore, it is crucial to answer the question to what extent long-term seed storage and the inextricably linked ageing is reflected in the miRNome. The material used in the study provided a unique opportunity to find answers to the questions about the role and relationship of miRNA with the process of seeds aging preserved in a dry state. As numerous studies have shown a pivotal role of miRNAs in senescence in both animals [50] and plants [51], the question arises about the role of miRNAs in seed aging. Recent studies on artificial seed aging have identified a relationship between miRNAs and the loss of seed viability [52,53]. Artificial aging, also known as accelerated aging, is an extensively used method in seed aging studies. The deterioration of the seeds quality is accelerated by affecting them with high temperature and humidity for a relatively short time [54,55]. Despite many years of investigations, researchers’ opinions on the uniformity of changes caused by accelerated and natural aging are divided. However, when analyzing the role of miRNAs in accelerated and dry aging, we are talking about two definitely different processes. The quiescent metabolism in dry seeds prevents the degradation of miRNA by exoribonucleases [56]. 

Taking into account previous studies indicating the presence of changes at the level of macromolecules caused by the process of seed aging, the presented here constant level of known and novel miRNAs in barley seeds regardless of their viability or storage time is quite surprising. Due to the lack of metabolic activity in dry seeds, it indicates very high stability of this non-coding RNA fraction. The uneven degradation of different RNA classes in barley seeds with low viability was indicated by earlier studies of Puchta et al. [57]. Although that study did not include the small non-coding RNA class, its results were consistent with uneven RNA degradation in male and female mouse hearts [58]. The high stability of miRNAs in vitro was also reported in other studies. Researchers indicated that miRNA molecules with increased GC-ratio or lacking AU/UA in the seed sequences and tail region had higher stability [59,60]. Extraordinarily interesting is the higher *in vitro* stability of miRNA compared to mRNA during NaOH, RNase A, Exonuclease T and Exonuclease T7 or Benzonase treatment [58]. Moreover, miRNAs seemed to be also insensitive to heat degradation [61]. These results suggest the stability of miRNAs to be generally robust. Therefore, it can be assumed that the lack of significant differences in miRNAs level of observed in the study results from their high stability. Moreover, it may also indicate that miRNA molecules are not destroyed by ROS, as happens with other macromolecules. 

In cells, miRNA are closely associated with RNA-induced silencing complex (RISC) and only a relatively small fraction is considered to be independent of RISC [62]. The association of miRNAs with the components of the RISC complex increases their stability [63]. The presence of RISC complex was confirmed by proteomic studies of barley seeds [64]. Besides, EMBL-EBI Expression Atlas indicates the highest expression of RISC is related to the development and germination of barley seeds [65]. Moreover, in plants, the stability of miRNA is additionally modified by 2′-*O*-methylation at 3′ end catalyzed by methyltransferase HEN1 [66]. Thus, in dry seeds, the miRNA-protein complex can provide an additional form of protection for this RNA fraction and prevents degradation during long-term storage.

The above-mentioned properties make miRNAs are the sole RNA faction identified so far that is not suffering from the deterioration associated with seeds aging. This discovery may have unprecedented significance for biobanks and indicate the direction of further research into the storage of human tissues and fluids. Perhaps, part of the material should be stored at low temperatures, but after prior freeze-drying.

### 3.2. miRNome of Barley Seeds

Analysis of embryos derived from dry seeds with quiescent metabolic activity revealed the presence of numerous isomiRs. Initially, the isomiRs observed in high-throughput sequencing results were thought to be either sequencing artefacts or to arise as a result of imprecise excision of miRNAs from the pre-miRNA hairpin by Dicer during biogenesis [67,68,69]. It is currently believed that isomiRs are biologically functional and have specific roles in plant cells [70]. 

The obtained results showed the presence of isomiRs differing from the canonical form only in length. They are most likely formed from a single pre-miRNA and are only length variants of the canonical form [71]. However, the data currently available in miRBase for barley are too limited to unequivocally state that they are not shared by several members of particular miRNA families.

All identified isomiRs were shorter than the canonical form. Their formation may therefore be related to partial degradation of miRNA molecules by exonucleases or to variable processing by Dicer [19,72,73]. The absence of 24 nt miRNAs may indicate a lack of Dicer-like protein DCL3 and DCL5 activity. DCL3 is involved in the biosynthesis of 24 nt long siRNAs derived mainly from transposons and repetitive DNA fragments and is involved in transcriptional gene silencing (TGS) [74,75,76]. In contrast, DCL5 reported in monocots is expressed in developing inflorescences and is responsible for the formation of 24 nt reproductive phasiRNAs [77,78]. Among the observed isomiRs, the 3′trimmed ones predominated, which is most likely due to nucleotide trimming at the 3′ end of the AGRONAUTE (AGO)-associated miRNA [79]. As shown by previous crystallographic studies of AGO, the 5′ end of the miRNA is located within the MID domain, whereas the 3′ end overhangs the PAZ domain and is thus more vulnerable to 3′–5′ exonuclease activity [80,81]. Trimming the 3′ end decreases the length of the miRNA molecule and increases its stability in the AGO protein [82,83]. The length of the miRNAs sequence is also an important factor determining sorting them into particular AGO complexes. Most miRNAs with a length of 21 nt bind to AGO1 and AGO2 while 24 nt miRNAs bind to AGO4 [84]. Therefore, it is suggested that the isomiRs length variation may be related to the strategy of target differentiation by assigning isomiRs to different AGO complexes [85]. 

Higher levels of 20 nt and 18 nt isomiRs of miR156 than its canonical form were also observed in the studied material. This indicates that isomiRs may regulate key processes at this developmental stage. Notably, previous studies have shown differential expression of miR156 isomiRs induced by drought stress in barley. The isomiRs regulated the expression of several genes including dehydration-responsive element-binding proteins [86]. Other studies confirm that indeed differential expression of isomiRs occurs tissue-specific and is associated with specific regulatory functions [87,88,89].

In the study, the most numerous group was 21 nt miRNAs, both for known and novel miRNAs. Many canonical 21 nt miRNAs have a high level of expression in plants [90]. The study of Ding et al. [91] showed 21 nt miRNAs constituted the major fraction. Generally, in plants, the common length distribution of miRNA sequences were between 21 and 24 nt, which was related to miRNA repetition [92]. Based on the differences in miRNA expression, it was found that there were characteristic expression profiles for dry and imbibed seeds, which is consistent with the results obtained by Ding et al. [91]. Previous studies concerning soybean shown that 22 nt small RNA class was highly abundant and was associated with repetitive sequences like rDNA and transposons. This class also played other functions than 21- and 24-nt small RNAs [92]. Such results were obtained for wild barley [14], wheat [9], corn [93], and soybean [92]. The length distribution in the Deng et al. [14] study on wild barley varied between 20–24 nt and the most abundant group was 21 nt (77.78%).

Six of the identified miRNA families, i.e., miR156, miR159, miR166, miR168, miR171, and miR397 occur both in mono- and eudicots, which may indicate that miRNAs are involved in regulating key genes for the development and functioning of plants [5]. Furthermore, the miR156, miR159 and miR166 take part in regulating of transcription of genes encoding activators and germination and dormancy repressors [5,13,94,95]. The families miR444, miR5049, and miR6201 were identified only in monocots, which suggests that they evolved as early as, at least, 90 Mya [96,97]. The families miR5051 and miR6200 were identified only in *H. vulgare*, which confirms that they are species specific. According to Wang et al. [98], most of the identified miRNAs in barley were highly conserved in monocotyledonous species, especially in the *Poaceae* family. Some miRNAs were present in mono- and eudicots plants, while others were specific to barley. Differences in miRNA occurrence may be affected by gains and losses due to evolution [13]. Data analysis has shown that some identified miRNAs had been sequenced only a few times, while others had been sequenced thousands of times. Among the identified conserved miRNA families, miR159, miR156, miR166, and miR168 showed the highest expression in all studied groups. Deng et al. [14] explained miRNAs high expressions by their critical role in growth and development of wild barley. The miR159 family, which showed the highest expression in the study regardless of the sample, regulates GAMYB transcription factor, which interacts in response to giberelic acid (GA) [99]. The miR156 was identified as one of the most expressed families in *H. vulgare* subsp. *spontaneum* (K. Koch) Thell. [14]. It regulates expression of a transcription factors family *SPL* (Squamosa promoter binding proteins like) genes [100,101]. The miR166 family that had the highest abundance in the early phase of maize seeds germination targeted DNA-binding transcription factor homeobox-leucine zipper in wild barley [5,14]. The miR168 family, that targets *AGO1* in *A. thaliana* and regulates RISC was also one of the most frequently expressed in the wild barley [14,102]. In general, the novel miRNAs identified here, were at low or even very low level. According to Wang et al. [5], differences in the number of readings associated with the level of expression are caused by the activity of various physiological and biochemical processes in the seeds at the moment. However, in dry seeds where metabolism is suppressed, the differences in the level of individual miRNAs will indicate which metabolic pathways will be triggered in the very initial germination phase. They may also be related to processes associated with the last phase of seed ripening and dormancy.

### 3.3. miRNA in Regulating Gene Expression Based on in Silico Analysis

Contrary to animals, in plants, almost perfect complementarity of miRNAs to their target transcripts is required. The target sites are located within the open reading frames (ORFs) of target gene [103]. Therefore, an efficient and accurate prediction method is based on a basic matching of plant miRNA and mRNA sequences [104]. The in-silico analysis performed here identified a total of 817 putative targets for miRNA found in dry seeds.

According to GO results, it is clear that targets’ biological functions are largely related to the metabolism of macromolecules. Both known and novel miRNAs regulate the catalytic activity associated with hydrolysis. It is well documented that in barley seeds both the scutellum and the aleurone layer are involved in the production and secretion of hydrolytic enzymes that catalyze the degradation of starch and protein reserves of the endosperm during germination [105]. In the initial germination phase of barley seed, amylolytic activity was detected almost exclusively in the region of epithelial cells of the scutellum [106]. Therefore, miRNAs derived from embryonic part of barley seeds may be responsible for regulating hydrolytic processes during initial germination phase. Among others, miR159, miR168 and miR171 can be involved in regulating starch hydrolysis. However, cells walls of endosperm constitute a potential barrier that limits access of hydrolytic enzymes to their substrates within endosperm cells. Thus, cell wall degradation is an important event in the early germination phase. Barley endosperm cells walls consist of about 70% (*w*/*w*) of (l-3,1-4)-p-glucans [107]. The (1-3,1-4)-beta-D-glucanase (EC 3.2.1.73) secreted during barley germination is responsible for their depolymerization [108]. The hvu-new41, that had significantly higher expression in seeds with the lowest viability might be involved in regulation of 1-3,1-4-beta-D-glucanase synthesis. During barley germination glucanase is synthesized de novo, mRNA encoding these enzymes increase during germination [109]. Thus, the increased content of hvu-new41 might be responsible for the removal of (1-3,1-4)-beta-D-glucanase transcripts and lowering or completely blocking the synthesis of this key enzyme for seed germination. However, experimental target verification for this novel miRNA is needed. It should also be stressed that in the case of dry seeds, in which metabolism is quiescent, the identified miRNAs do not regulate gene expression exactly at the moment of performing the analysis. Their role is to regulate genes which expression will be initiated only at the moment of the environmental conditions favorable for germination occurrence, i.e., at the moment of seed rehydration. Thus, miRNAs accumulated in dry seeds represent a guarantee of proper regulation of gene expression at the next developmental stage. The phenomenon is that it may take place even over several decades.

## 4. Methods

### 4.1. Plant Material

**Figure 8:**
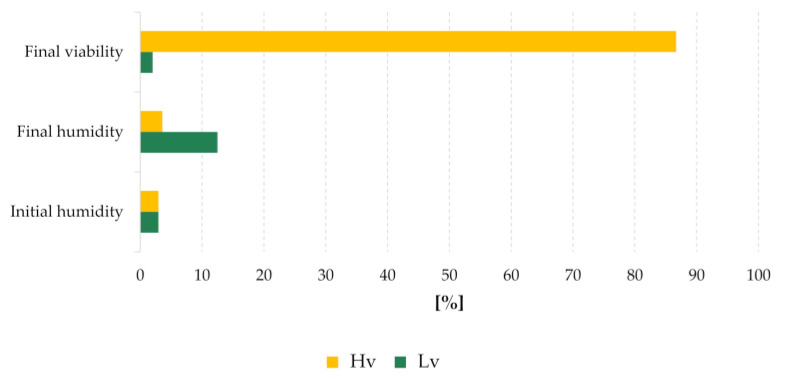
*Hordeum vulgare* cv. ‘Damazy’ seeds parameters.

Barley seeds of cv. ‘Damazy’ harvested in 1972, were dried to 2.96% water content and seed germination tests were carried out. Seeds which showed viability above 95% were placed in two air-filled glass flasks and hermetically sealed. All flasks were stored under the same conditions, i.e., in a basement with limited light, at an ambient temperature (10–25 °C) and a relative humidity in the room of about 80–90%. Germination tests were carried out again in 2015 and it turned out that seed viability differed between flasks. A sample of the high viable seeds was taken, and the remaining seeds were then stored further. The high viable sample was regenerated in the field (in 2017/2018) according to the gene bank standards and provided the reference material after drying them to a low water content (approx. 7%). In 2018, seed viability and moisture content (Figure 8) were evaluated and three groups were selected for the study: seeds stock with high viability (Hv) 86.7% and moisture content 3.58%, seeds with low viability (Lv) 2% and moisture content 12.5% and regenerated seeds (Rc), which were a reference sample. Three biological replicates of 25 dry seeds each were used at every stage of the study.

### 4.2. miRNA Extraction and Library Preparation

The initial step in miRNA isolation was the dissection of the part containing the embryo and the scutellum from dry seeds. This procedure was performed to reduce the amount of starch, which negatively affects the effectiveness of miRNA isolation. Parts of the seeds prepared in this way were crushed into fine powder in a mortar under liquid nitrogen. miRNA was isolated using a Micro RNA kit (A&A Biotechnology, Gdynia, Poland). The miRNA was quantitatively evaluated using Nanodrop 1000 spectrophotometer (Thermo Fisher Scientific, Waltham, MA, USA) and agarose electrophoresis. To confirm the results and evaluate miRNA integrity 2100 Bioanalyzer with Small RNA Kit (Agilent Santa Clara, Santa Clara, CA, USA) was used. Small RNA libraries were prepared from three biological replicates of isolates based on the NebNext Multiplex Small RNA Library Prep Set for Illumina (New England BioLabs, Ipswich, MA, USA). miRNA libraries were cleaned of post-reaction elements by Monarch Kits for RNA Cleanup kit (New England BioLabs, Ipswich, MA, USA). The size selection was performed by Pippin prep 3% Agarose Gel Cassette (Sage Science, Beverly, MA, USA). The final quality of the libraries was evaluated by Agilent Bioanalyzer 2100 High Sensitivity DNA Analysis and the Qubit dsDNA HS Assay Kit (Thermo Fisher Scientific, Waltham, MA, USA). The miRNA was finally sequenced on MiSeq (Illumina) using Reagent Kits v2 (50-cycles) in the 51 single-end modes.

### 4.3. Bioinformatic and Statistical Analysis

The quality of the readings from the Illumina sequencing was evaluated using FastQC [110]. The raw data was filtered: adapters, low-quality sequences (Q < 30), and sequences shorter than 17 bp and longer than 25 bp were removed. The UEA Small RNA Workbench: Adapter Removal and Filter software was used to remove the adaptor sequences, sequences containing ambiguous nucleotides, and t/r RNA contamination [111]. High-quality reads were forwarded for further analysis. Using the Sequence Alignment UEA Small RNA Workbench [112], the miRNA sequences were mapped to the *Hordeum vulgare* reference genome (Hv_IBSC_PGSB_v2) obtained from Ensembl Plants database release 40 [113] accessed 16.03.2020. To find known miRNAs deposited in the miRBase version 22.1 database [11] and their isoforms, analyses were performed using the UEA Small RNA Workbench miRProf with no mismatch allowed. Identification of novel miRNAs was performed in the miRCat UEA Small RNA Workbench tool. The software was used with plant parameters (genome hits = 16, hit dist = 200, max gaps = 3, max overlap percentage = 80, max percent unpaired = 50, max unique hits = 3, maxsize = 25, min abundance = 1, min energy = −25, min gc = 20, min hairpin length = 60, min paired = 17, min size = 18, orientation percentage = 80, hairpin extension = 100, *p*-value = 0.05).

The total number of mapped reads from given library was used for normalization to reads per million (RPM) of novel miRNA read abundance. The sequence was considered as a miRNA candidate, when its secondary structure met the criteria reported by Axtell and Meyers [20]. Novel miRNAs were assigned to the known miRNA families in miRBase 22.1 using miRclassifity [114]. Prediction of target genes for the found miRNAs was implemented in psRNATarget software (https://plantgrn.noble.org/psRNATarget/, accessed on 12 February 2021) [21] with default Schema V2 (2017 release) with an expectation score up to 5 and the length complementarity scoring was 17. Quantitative analyses were carried out using DESeq2 from SARTools R package [115]. The results were presented in a graphical form using ggplot2 [116] package for R. ANOVA analysis (*p* < 0.05) with post hoc Tukey’s HSD test by XLSTAT ECOLOGY 2017.1.1 (Addinsoft) were used to determine the relevant differences between the samples and the miRNA in the family. In order to define potential roles of *H. vulgare* miRNA in the biological and molecular process, Gene Ontology (GO) annotations of miRNA target genes were downloaded from UniProt identifiers GO categories represented by predicted targets of miRNA were demonstrated using g:Profiler toolset [22]. Raw data is available in the Open Science Framework repository under identifier HDG78 and NCBI GEO record GSE164512.

### 4.4. RT-qPCR

A RT-qPCR (Reverse transcription qPCR) reaction was performed to confirm the results obtained during deep sequencing for statistically significant miRNAs [117]. miRNAs occurring in each of the samples and showing a stable level of expression in the samples were chosen as the reference miRNA using RefFinder [118]. Primers for sequences of mature miRNA were designed using miRprimer software [119] and the primer sequences are presented in Supplementary Table S7. miRprimer is an automatic method for design of functional primers for miR-specific RT-qPCR. The cDNA synthesis reaction with miRNA was carried out using the Mir-X™ miRNA First-Strand Synthesis Kit (Takara Bio, Kusatsu, Japan) and 400 ng miRNA, the reactions were conducted according to the manufacturer’s protocol. Real Time PCR reactions were performed with the following composition: 2 µL 1× HOT FIREPol EvaGreen qPCR Mix Plus (Solis BioDyne Tartu, Estonia), 0.25 µM primers, 5.5 µL water and 2 µL 20-fold diluted cDNA. The reaction was carried out under the following conditions: initial denaturation: 60 °C/15 min, 40 cycles: denaturation 95 °C/25 s, hybridization of primers 60 °C/25 s, elongation 72 °C/25 s and final elongation 72 °C/5 min, the reaction was completed in 4 °C. The analysis was performed in Rotor-Gene 6000q series (Corbett Life Science, Mortlake, Australia) thermocycler. All analyses were performed in three biological and three technical replicates. The expression of the investigated miRNAs was calculated based on the ^ΔΔ^Ct analysis. Three miRNAs, namely miR159, miR166, and miR168, were used as references.

## 5. Conclusions

The results presented here clearly indicate that miRNAs are not destroyed during the long storage of barley seeds in a dry state. The level of the majority of known and novel miRNAs remained stable regardless of whether the seeds were fully viable or had very low germination capacity, as well as whether they were long-term stored or non-stored. Therefore, it can be concluded that this is the only RNA class which does not suffer deterioration due to the aging of the seeds. The novel miRNA (hvu-new41) was the only one that had a significantly higher expression in seeds with low germination ability. Further research is needed to verify whether it actually regulates 1-3,1-4-beta-D-glucanase synthesis. The analysis of the degradome and detailed analysis of the transcriptome of the same plant material are ongoing and the results will be published soon.

## Figures and Tables

**Table 1:** Data set summary of sequencing of the two small RNA libraries.

Small RNA Data	Raw Sequencing Reads	Clean Reads	Mapped Reads	t/rRNA Matches	Match miRNA
Rc-1	2,496,964	672,701	239,836 (35.65%)	54,665 (8.13%)	874(0.13%)
Rc-2	2,593,309	657,323	240,539 (36.59%)	66,134 (10.06%)	1103 (0.17%)
Rc-3	5,425,372	1,116,857	438,066 (39.22%)	40,201 (3.60%)	1740 (0.16%)
Lv-1	2,258,988	511,598	173,417 (33.90%)	61,253 (11.97%)	725 (0.14%)
Lv-2	4,174,307	930,756	324,366 (34.85%)	111,254 (11.95%)	1444 (0.16%)
Lv-3	4,635,289	1,085,034	390,864 (36.02%)	127,376 (11.74%)	1553 (0.14%)
Hv-1	2,521,775	626,232	197,658 (31.56%)	79,540 (12.70%)	867 (0.14%)
Hv-2	3,369,389	821,586	286,502 (34.87%)	85,666 (10.43%)	1083 (0.13%)
Hv-3	4,932,306	1,162,680	430,005 (36.98%)	120,497 (10.36%)	1711 (0.15%)

**Table 2:** Novel miRNAs matched to known families.

Novel miRNA	Matched miRNA	Probability
hvu-new80	miR159	0.77
hvu-new45	miR166	0.62
hvu-new49	miR167	0.28
hvu-new50		0.33
hvu-new51		0.2
hvu-new52		
hvu-new53		
hvu-new43	miR168	0.7
hvu-new69	miR396	0.4
hvu-new71	miR171	0.6
hvu-new73	miR397	0.5

**Table 3:** Target prediction and their encoding proteins.

Target Accession	Expectation	Target Descriptor	Gene	Uniprot Protein Accession
**hvu-new60**				
BF261584	3.5	Precursor of CP29, core chlorophyll a/b binding (CAB) protein of photosystem II	*N/A*	Q40039
TC240558, TC267263, TC276211, TC257011, BI953040, TC275119, BI955619, BJ482334	4.5			
BI951019	4	Hordoindoline-B1	*HINB-1*	Q9FSI9
TC265164	4	Rust resistance gene ABC1041	*ABC1041*	Q2L7E7
TC251527	5	Cystatin Hv-CPI7	*icy7*	Q1ENE8
TC238665	5	NADPH-dependent thioredoxin reductase isoform 2	*NTR2*	A9LN30
**hvu-new41**				
TC238419, BJ468992	3.5	Boron transporter	*Bot1*	A9XTK3
TC267583	4.5	Basic helix-loop-helix protein	*HvIRO2*	Q0KKX2
DN185239	4	Glb 1 1-3,1-4-beta-D-glucanase precursor (Lichenase precursor)	*N/A*	Q02345
TC277443, TC239158, TC252681	5	Knotted 7	*kn7*	Q717U4
**hvu-new2**				
TC241603	2.5	Glyceraldehyde-3-phosphate dehydrogenase	*GAPC*	P26517
TC256820	4	Non-specific lipid-transfer protein 1	*LTP1*	P07597
TC238588	3.5	Germin-like protein 5a	*GER5a*	Q0GR06
BI949246	3	B hordein	*N/A*	Q40026
TC278915	4	Ribulose bisphosphate carboxylase large chain	*rbcL*	P05698
TC240271	3.5	Ribulose bisphosphate carboxylase/oxygenase activase B, chloroplast	*RCAB*	Q42450
BI950805	2.5	Pathogenesis-related protein PRB1-2	*N/A*	P35792
TC247384	2.5	Sucrose-phosphatase	*N/A*	Q84ZX7
TC273036	4	Beta-amylase	*Bmy1*	Q9AVJ8
TC279634	3	14-3-3-like protein A	*N/A*	P29305
TC275451	3	Hordoindoline-b1	*hinb-1*	Q5IUH9

## Data Availability

The data presented in this study are openly available in NCBI GEO at https://www.ncbi.nlm.nih.gov/geo/query/acc.cgi?acc=GSE164512 accessed on 12 February 2021, reference number GSE164512.

## Supplementary Materials

Supplementary Materials can be found at https://www.mdpi.com/article/10.3390/ijms22094315/s1. Table S1. An average level of expression of known miRNAs identified in the tested seed samples; Table S2. An average level of expression of novel miRNAs identified in the tested seed samples; Table S3. Hairpin structures of novel miRNA; Table S4. Results of Differential Expression (DE) analysis; Table S5. Targets for known and novel miRNAs; Table S6. Results of GO analysis; Table S7. Primers used in the RT-qPCR of miRNA; Figure S1. Novel miRNA hairpins structure.

## Abbreviations

miRNA	microRNA
sRNA	small RNA
siRNA	small interfering RNA
ta-siRNA	trans-acting siRNA
Rc	renewed seeds
Hv	seeds with high viability
Lv	seeds with low viability
rRNA	ribosomal RNA
tRNA	transfer RNA
NGS	next generation sequencing
RT-qPCR	quantitative reverse transcription-PCR
