# Supplementary material for: Barley Seeds miRNome Stability during Long-Term Storage and Aging
Source: Int J Mol Sci. 2021 Apr 21;22(9):4315. doi: 10.3390/ijms22094315 (PMC8122619; doi:10.3390/ijms22094315)

Hvu-new1

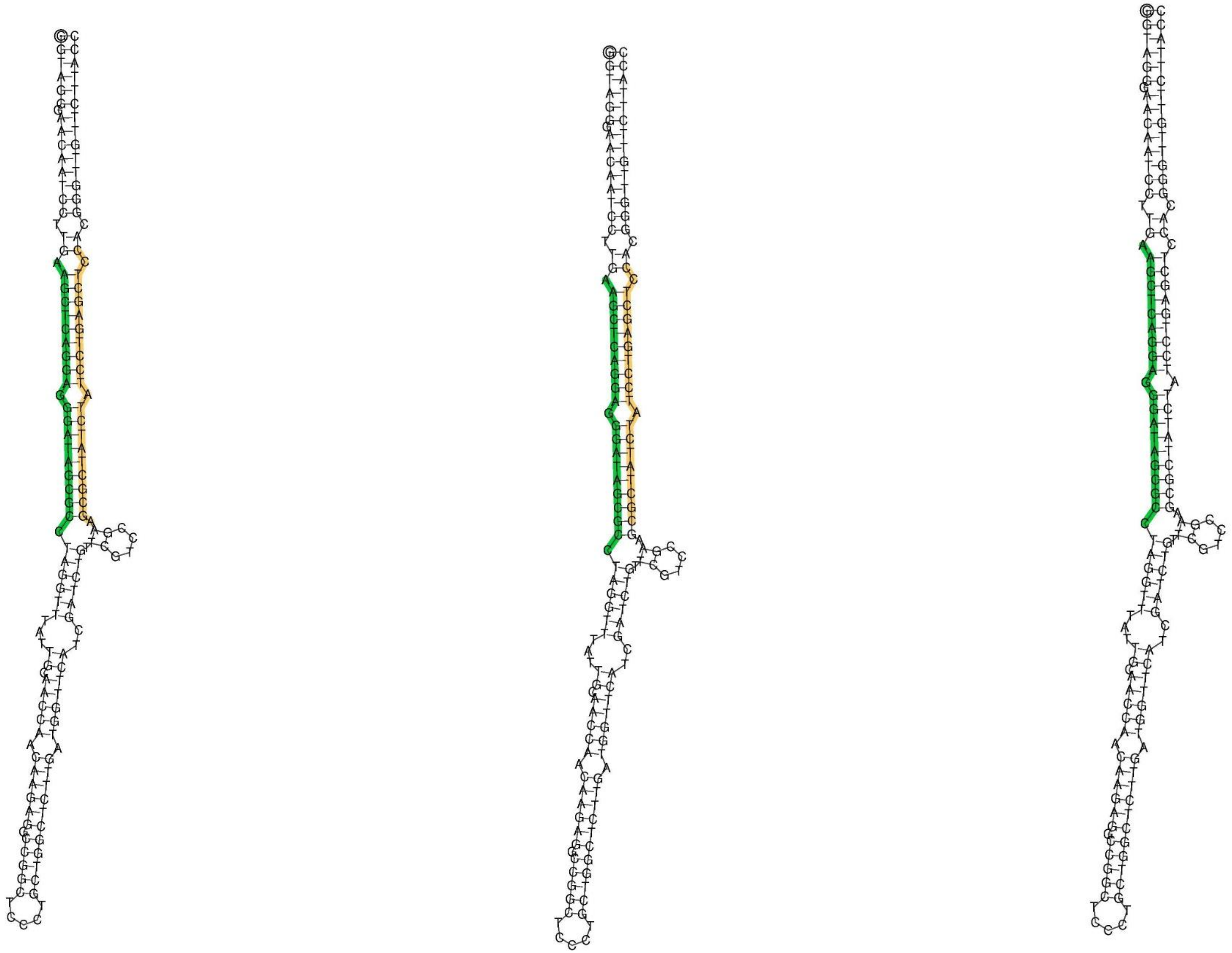

Hvu-new2

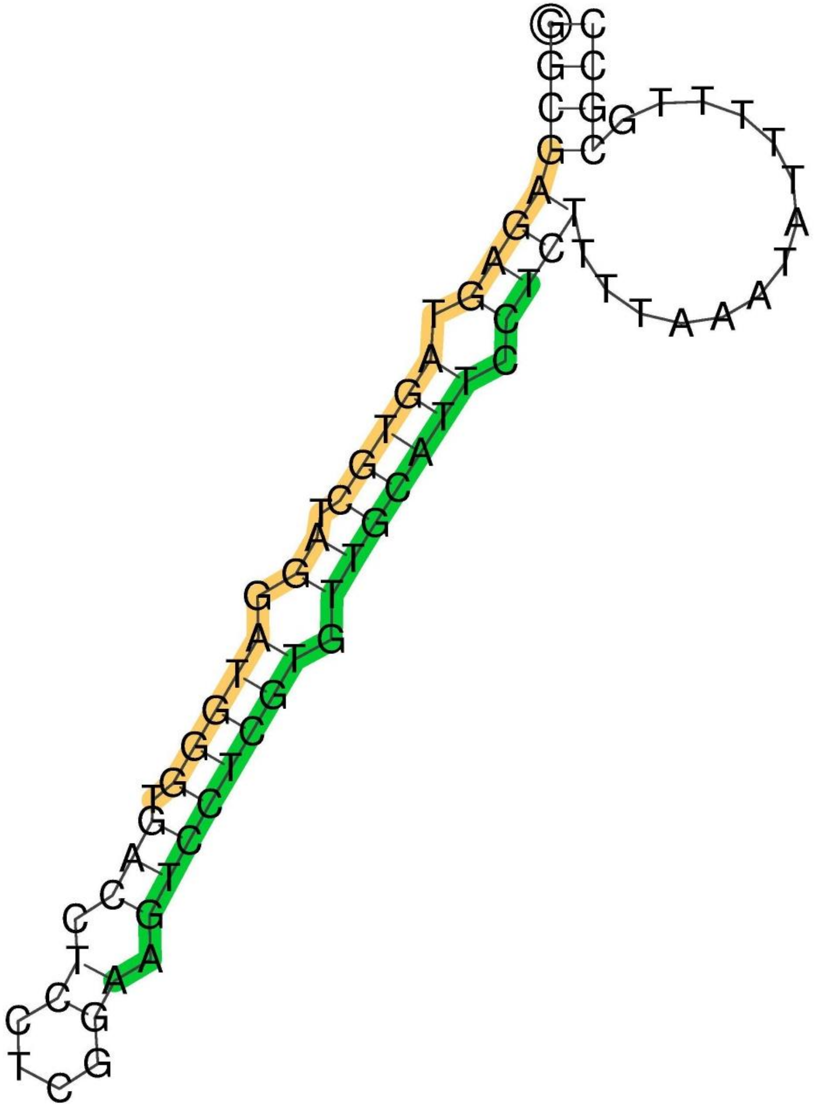

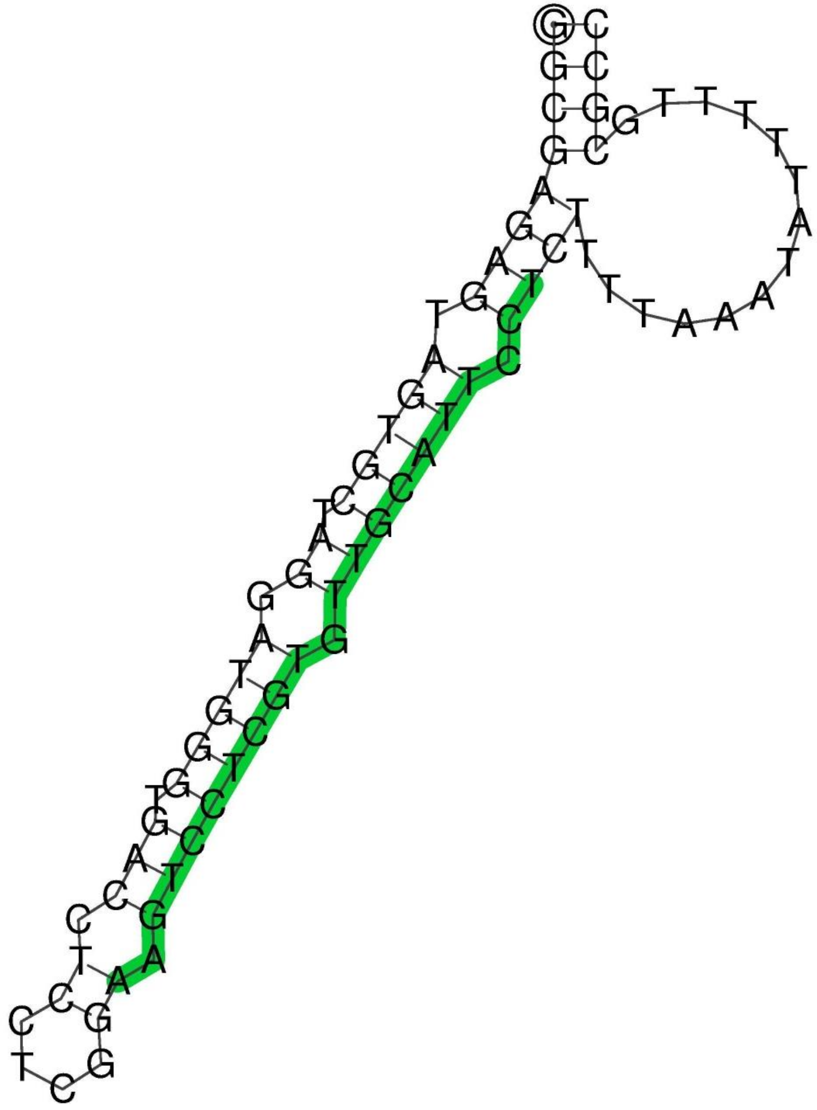

Hvu-new3

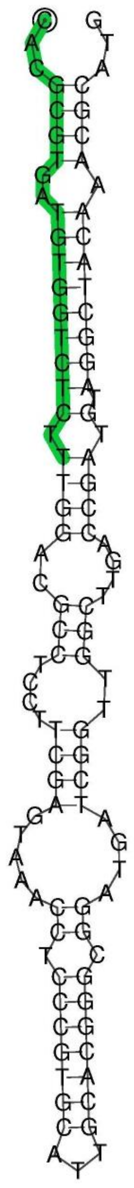

Hvu-new4

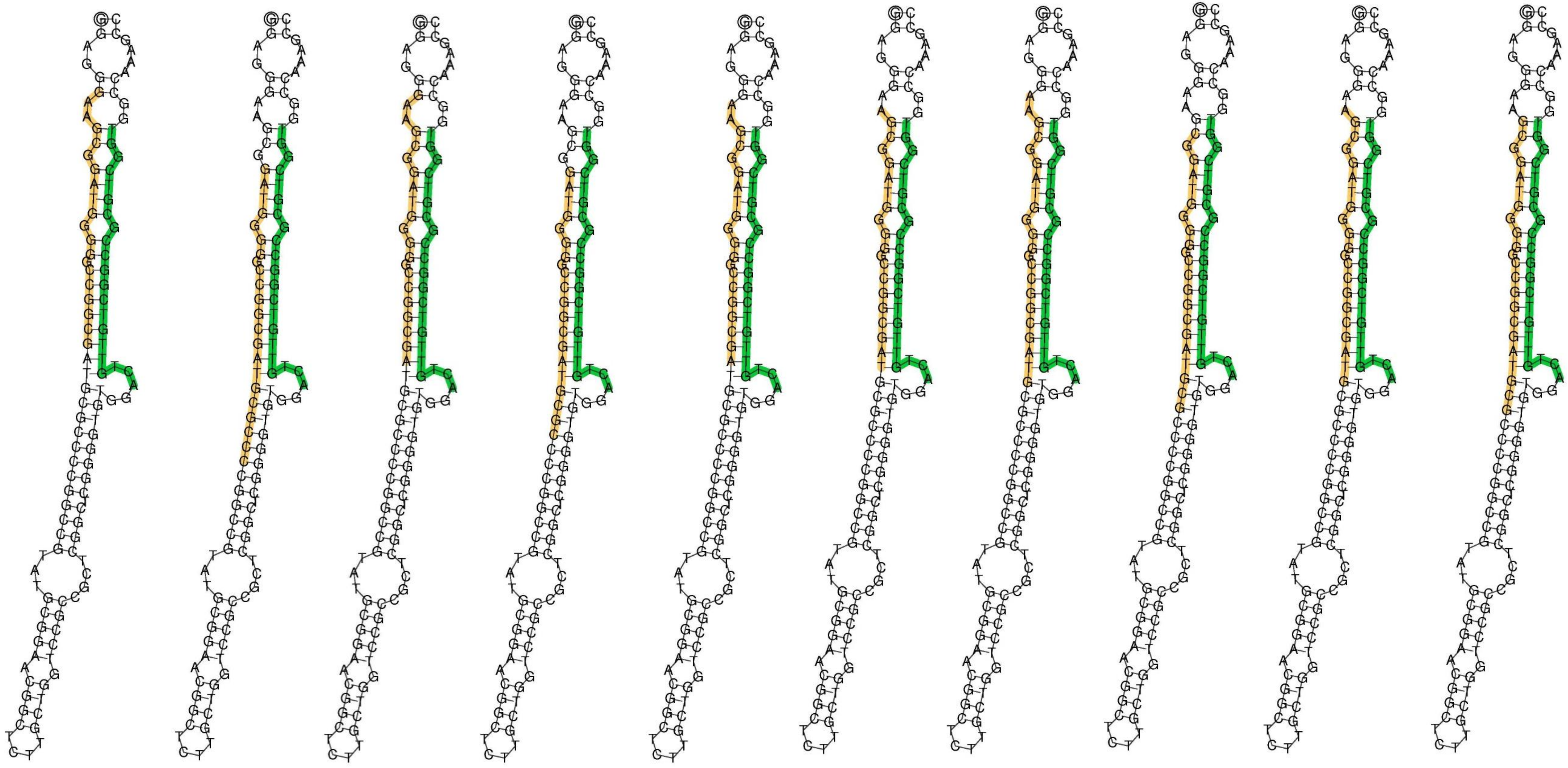

Hvu-new4

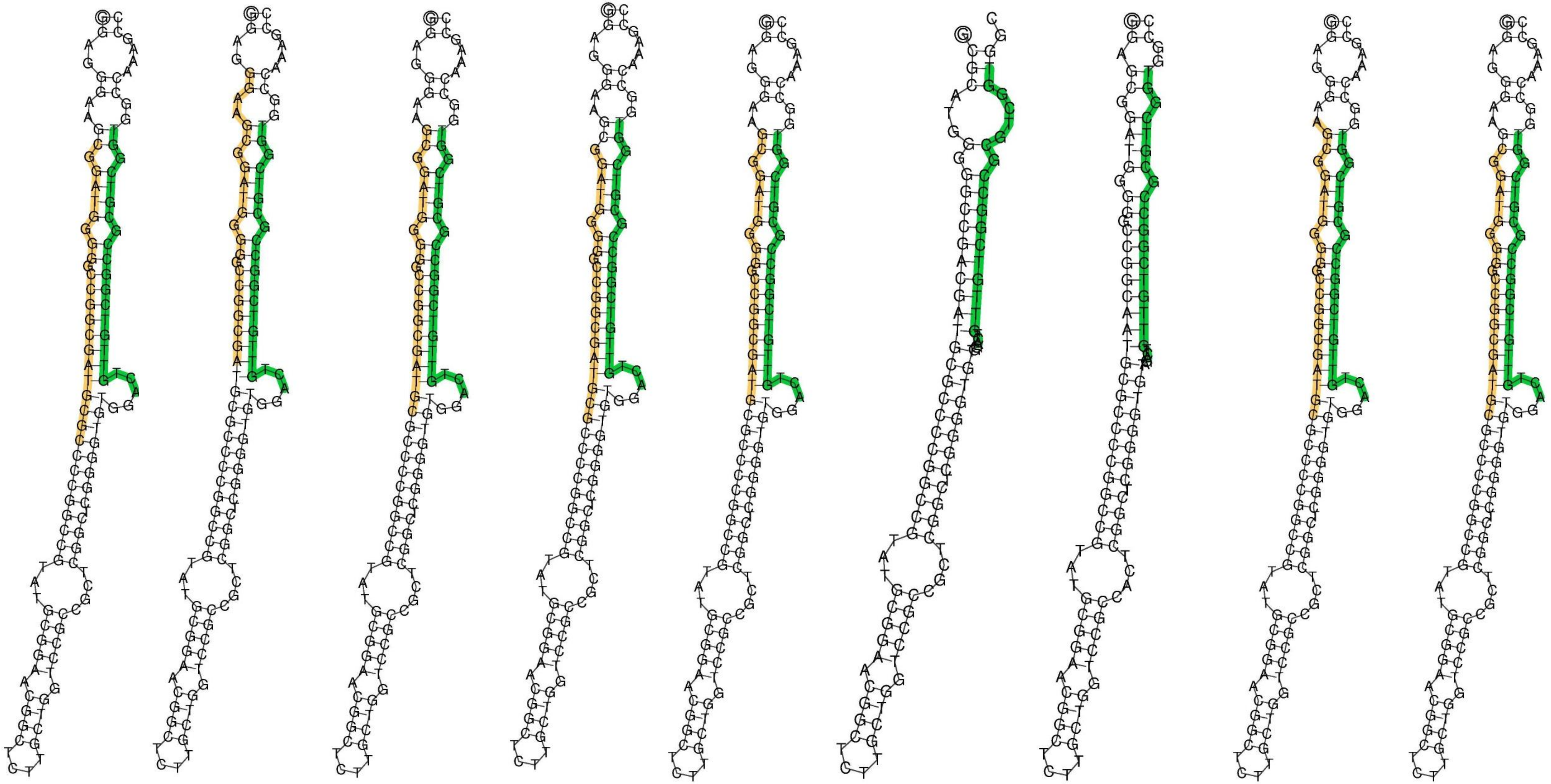

Hvu-new5

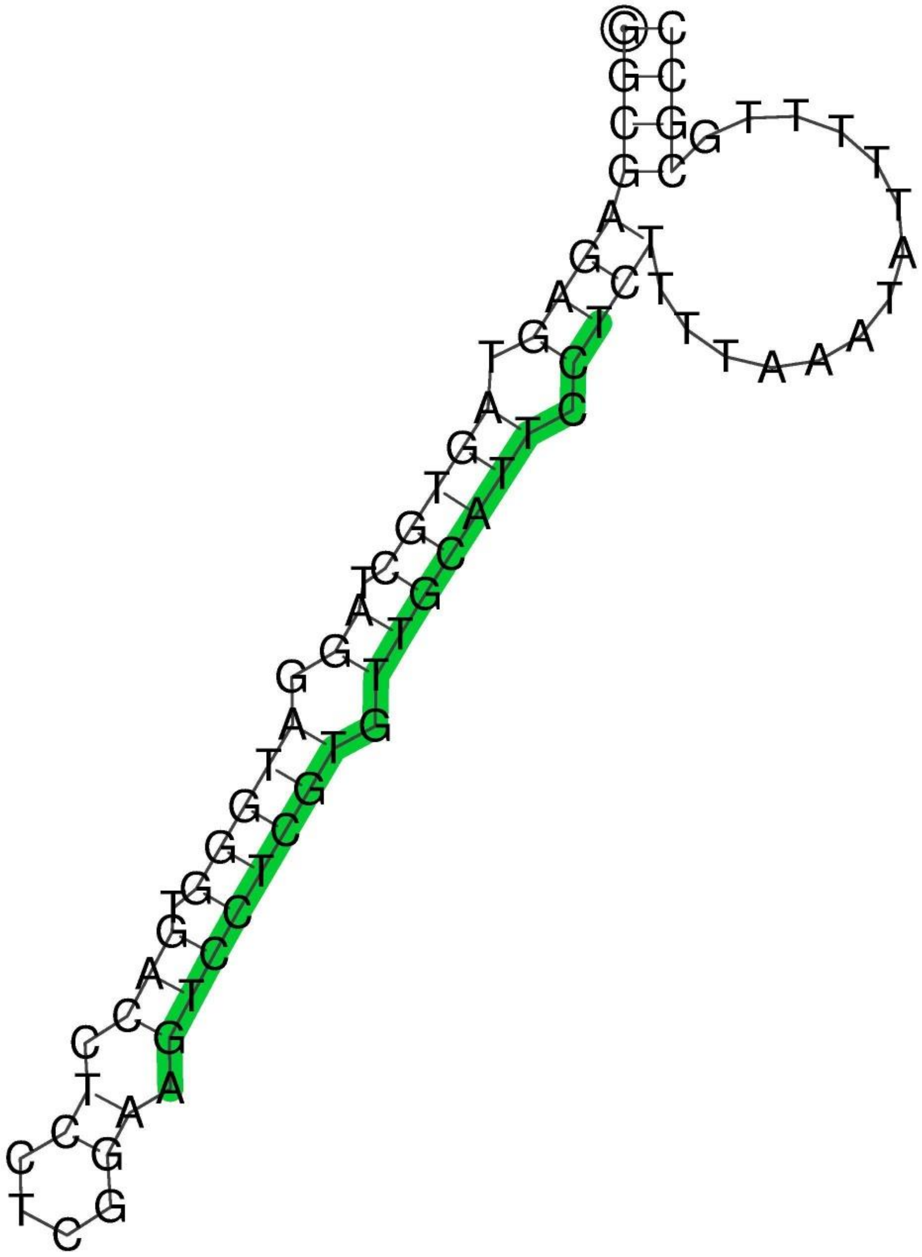

Hvu-new6

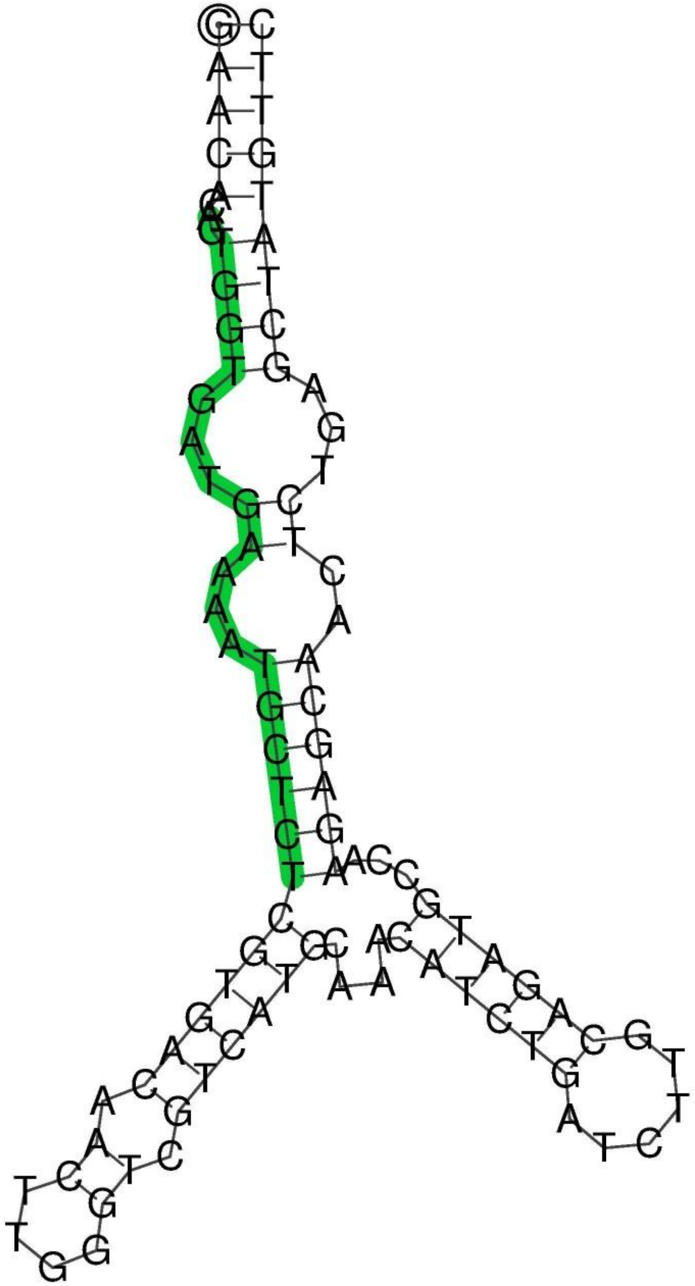

Hvu-new7

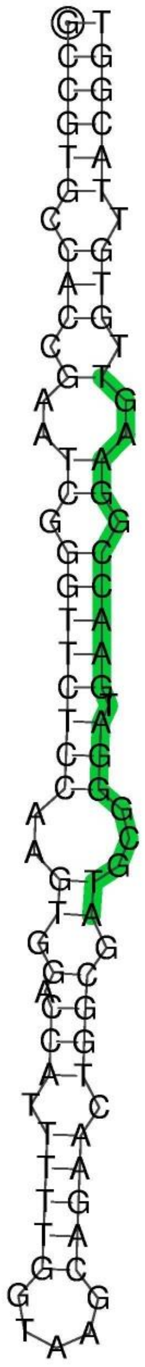

Hvu-new8

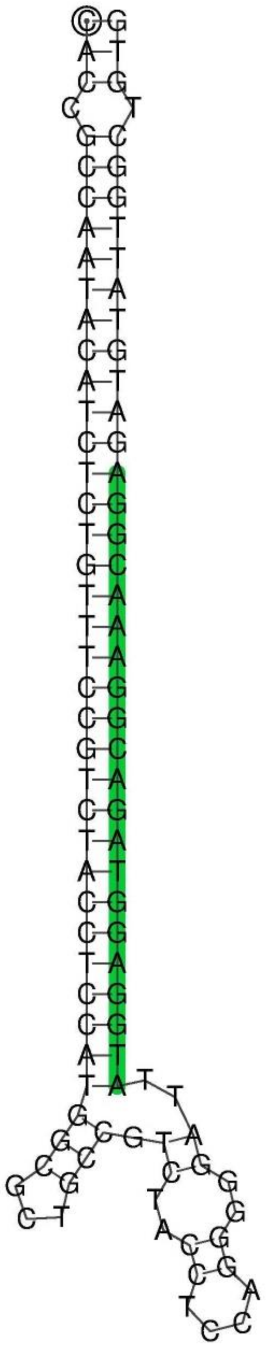

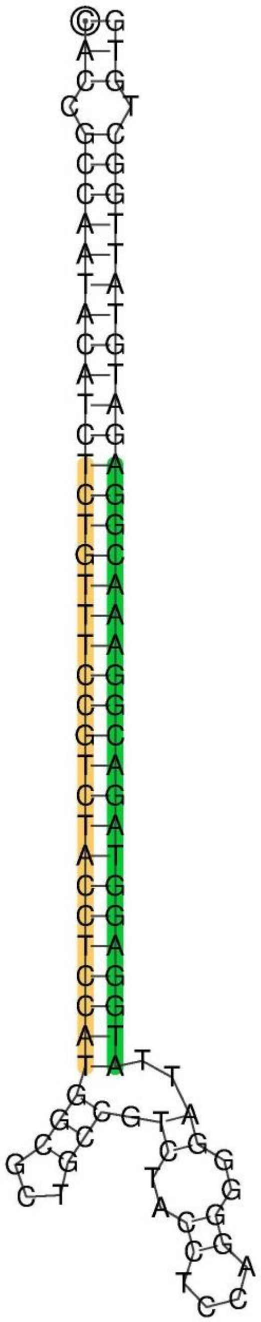

Hvu-new9

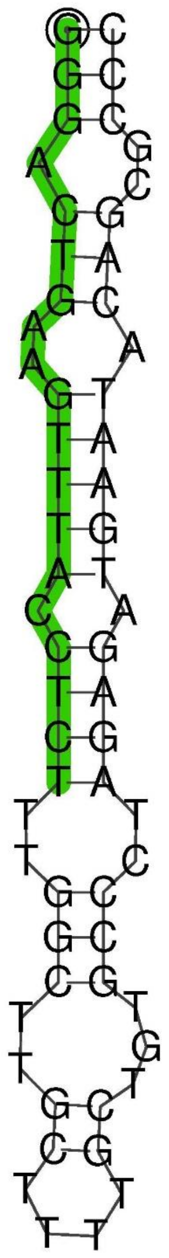

Hvu-new10

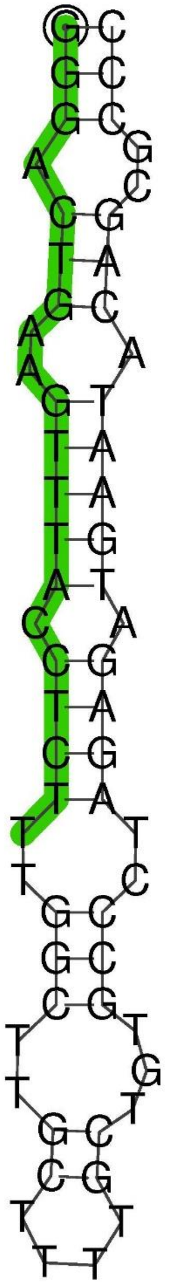

Hvu-new11

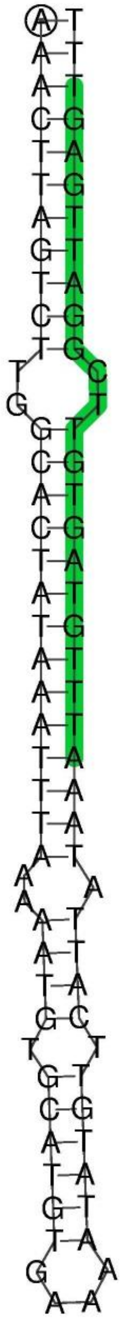

Hvu-new12

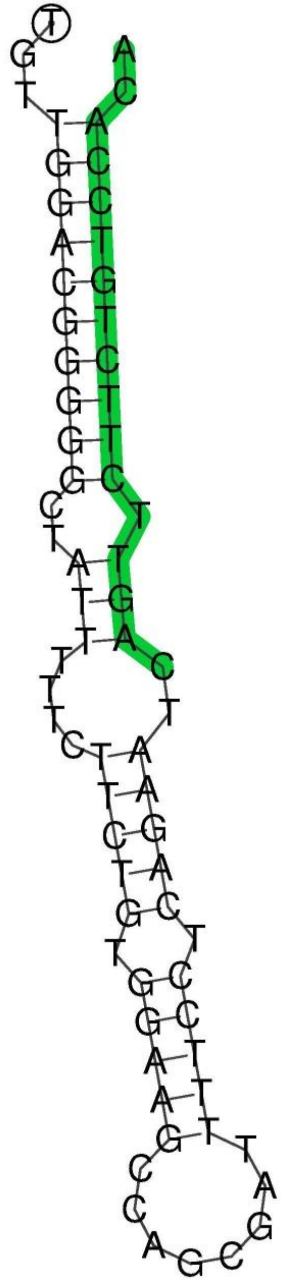

Hvu-new13

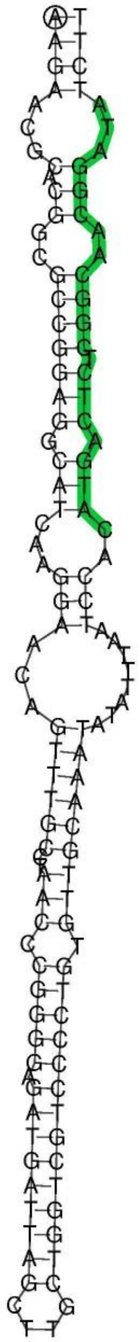

Hvu-new14

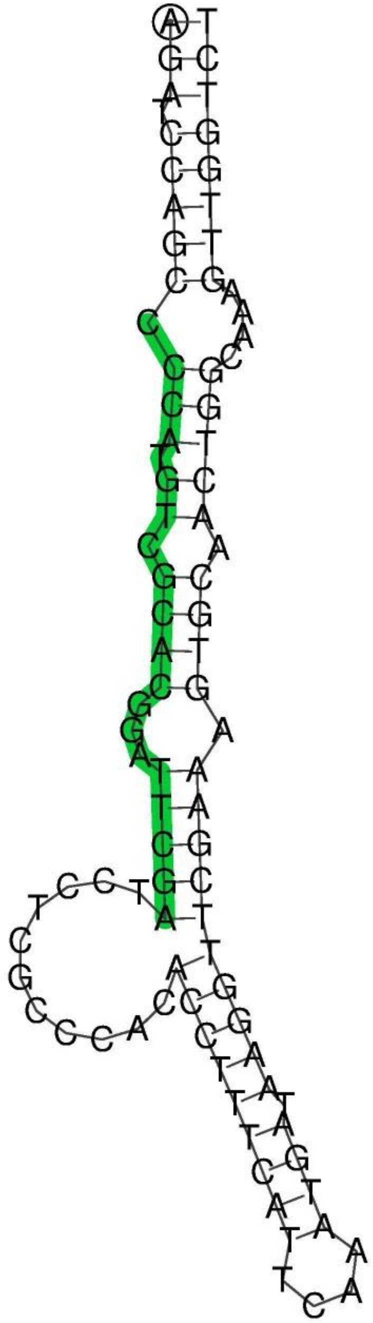

Hvu-new15

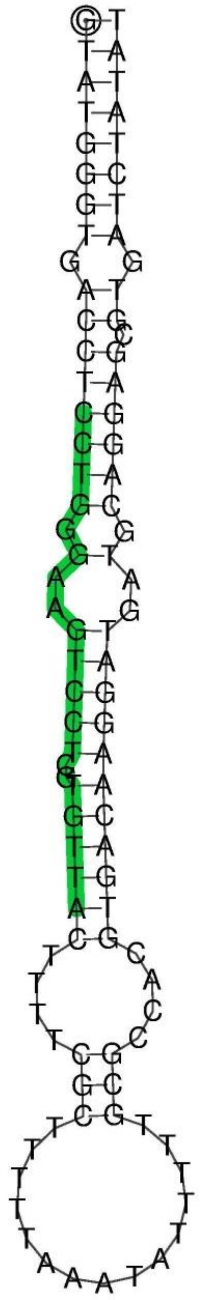

Hvu-new16

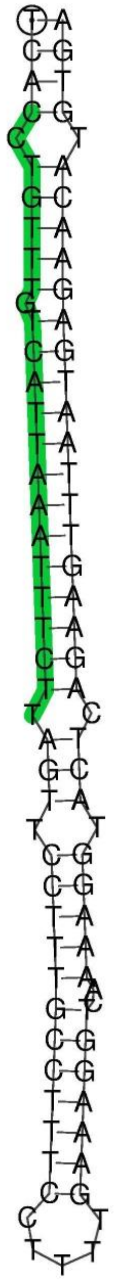

Hvu-new17

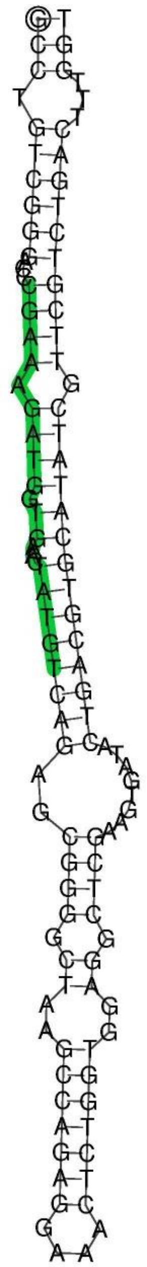

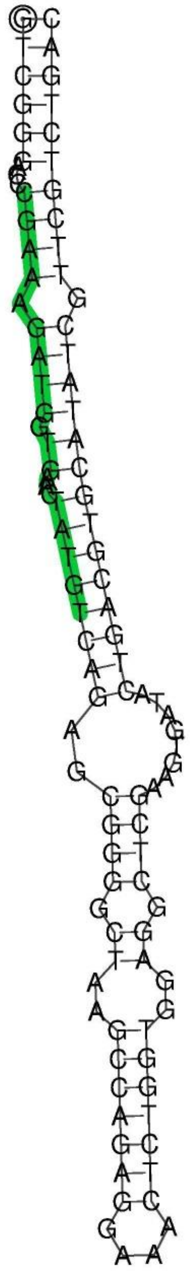

Hvu-new18

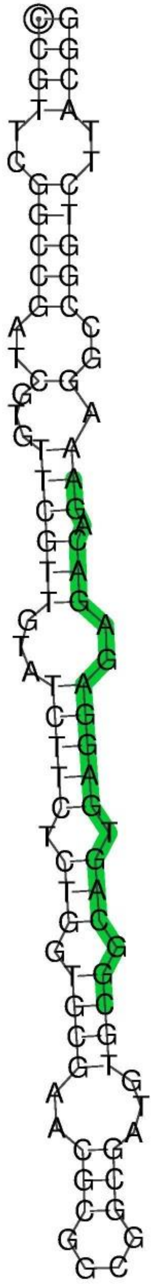

Hvu-new19

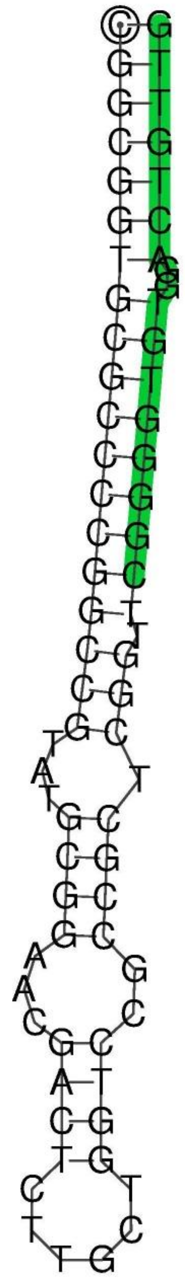

Hvu-new20

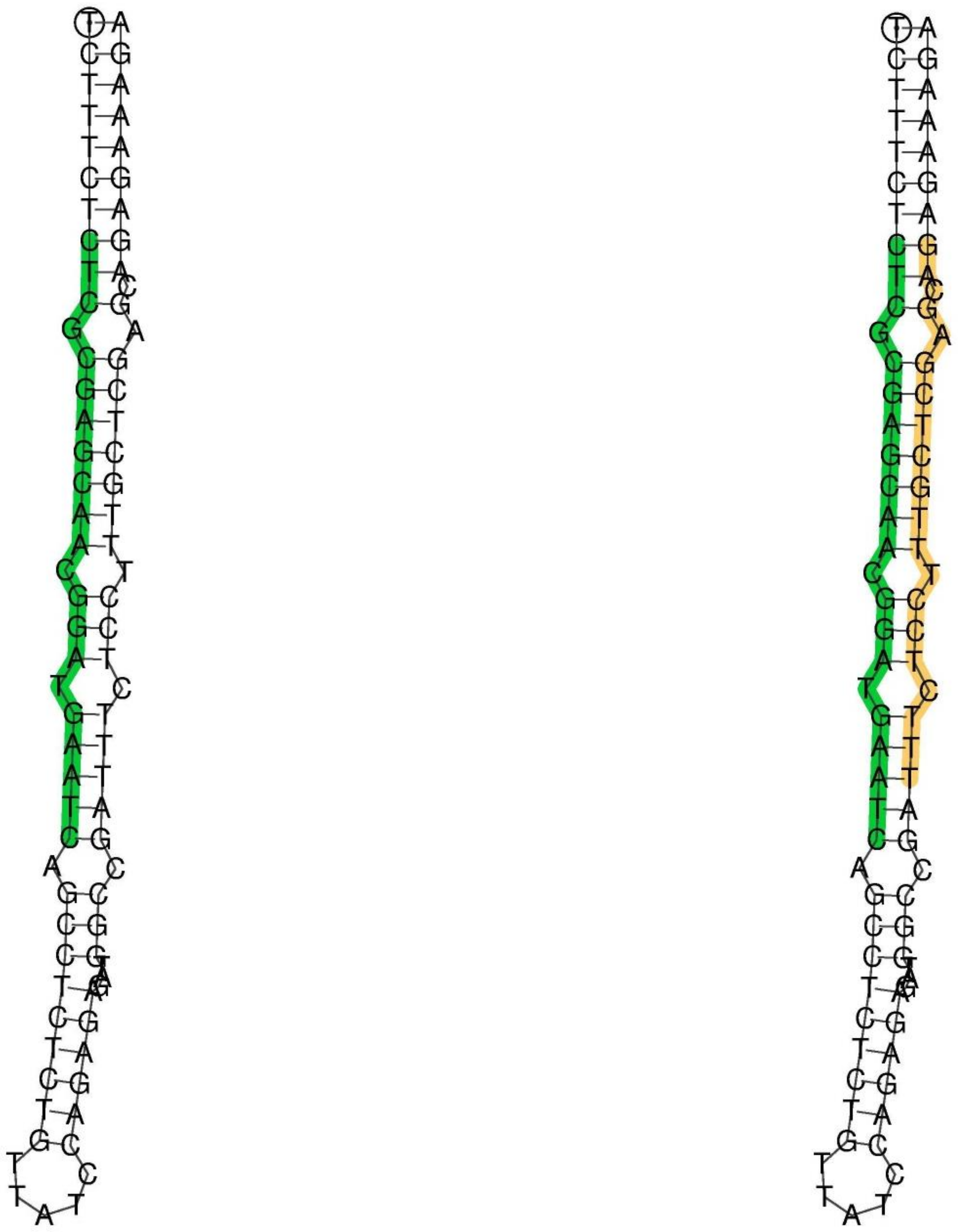

Hvu-new21

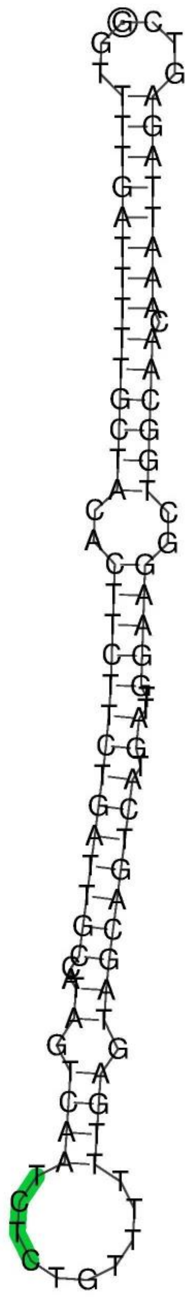

Hvu-new22

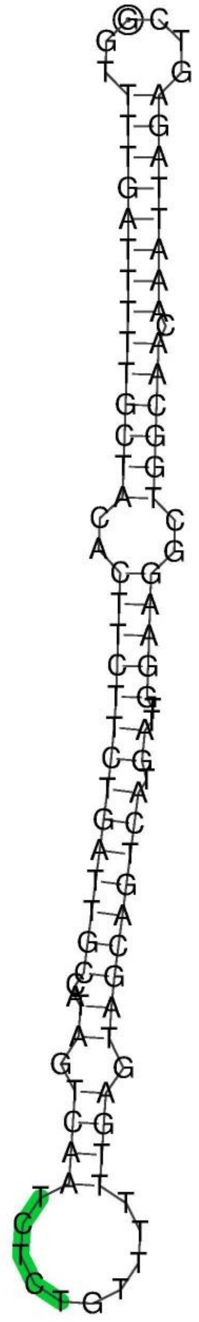

Hvu-new23

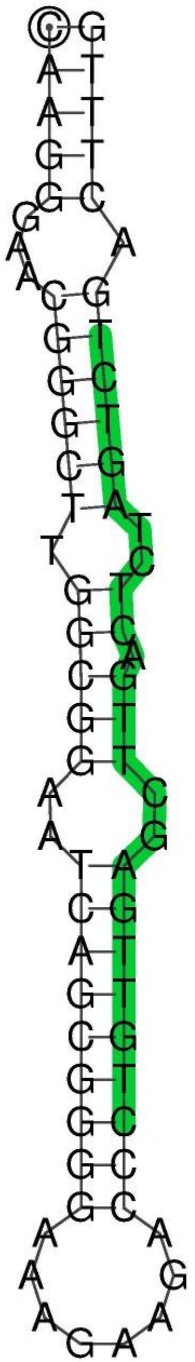

Hvu-new24

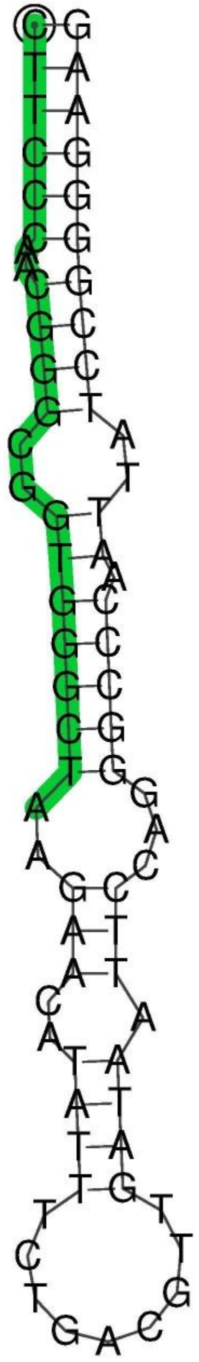

Hvu-new25

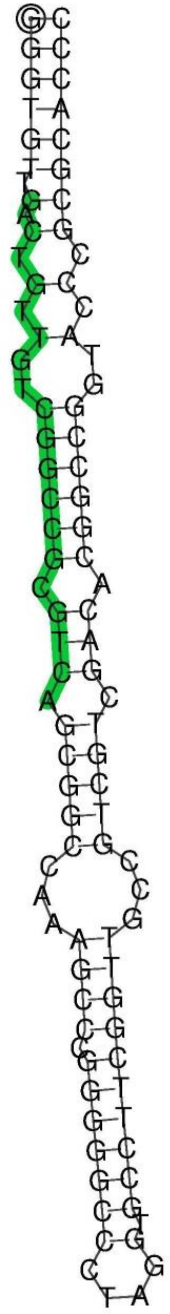

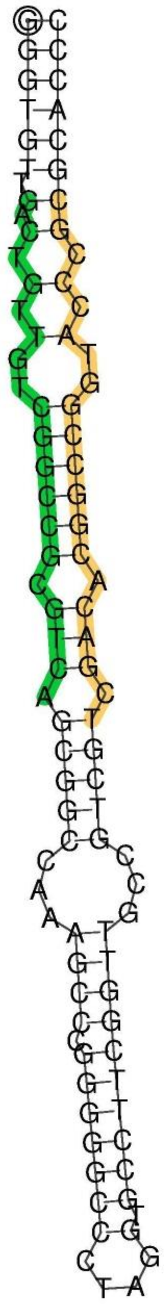

Hvu-new26

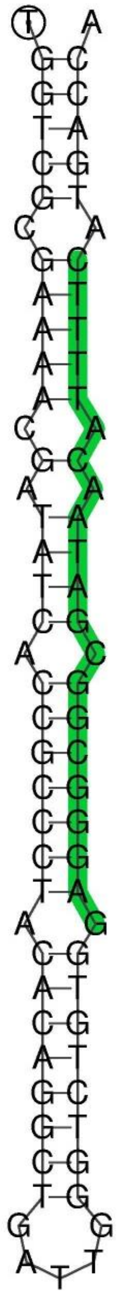

Hvu-new27

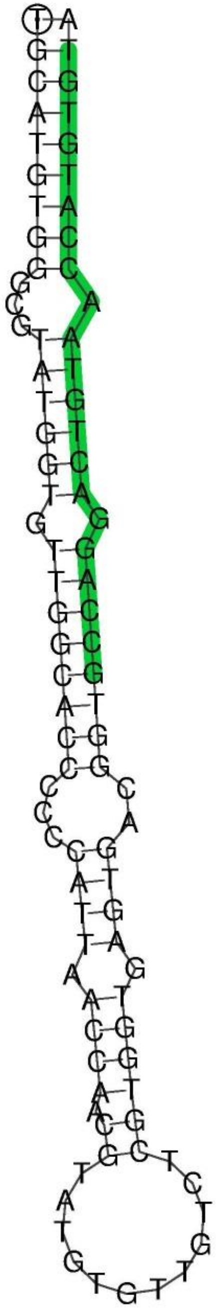

Hvu-new28

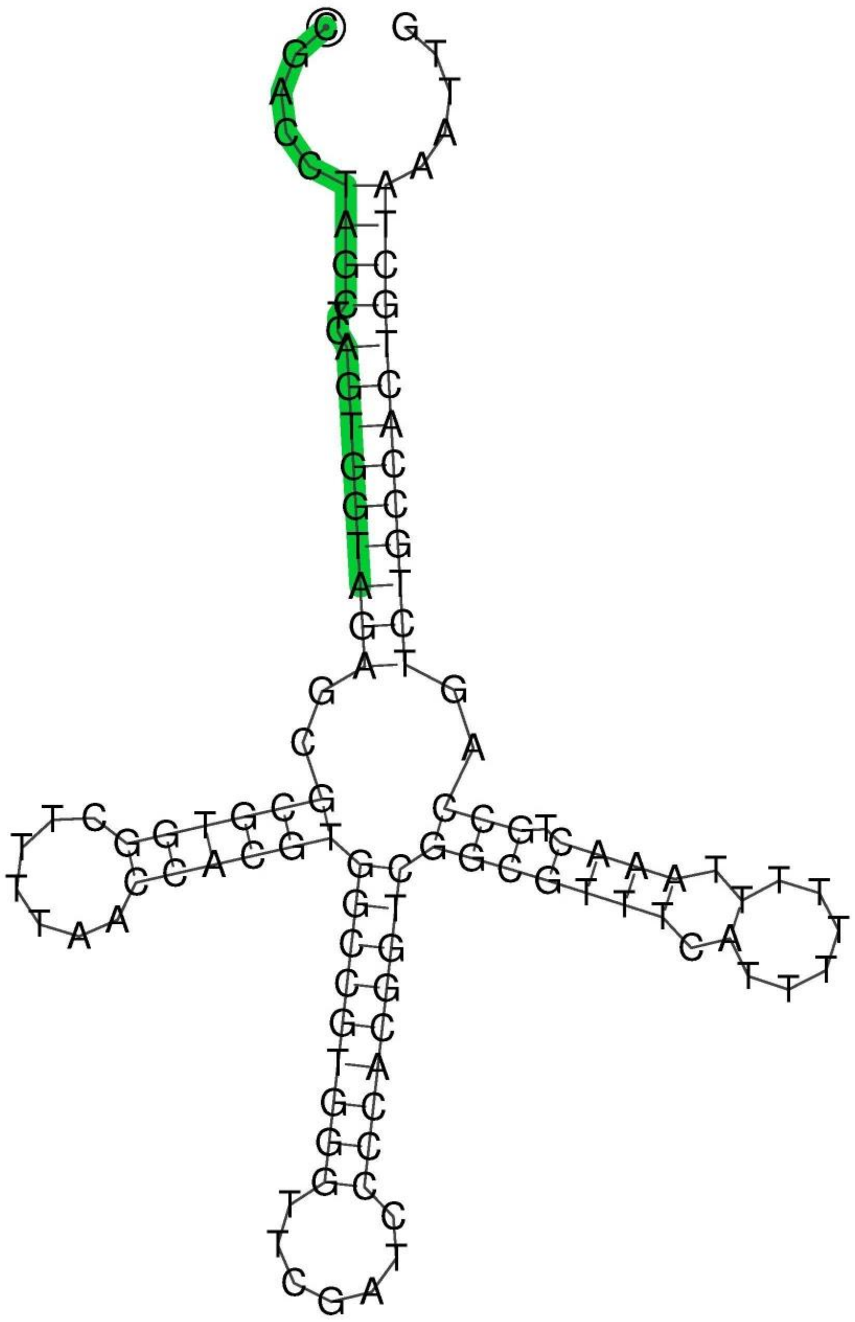

Hvu-new29

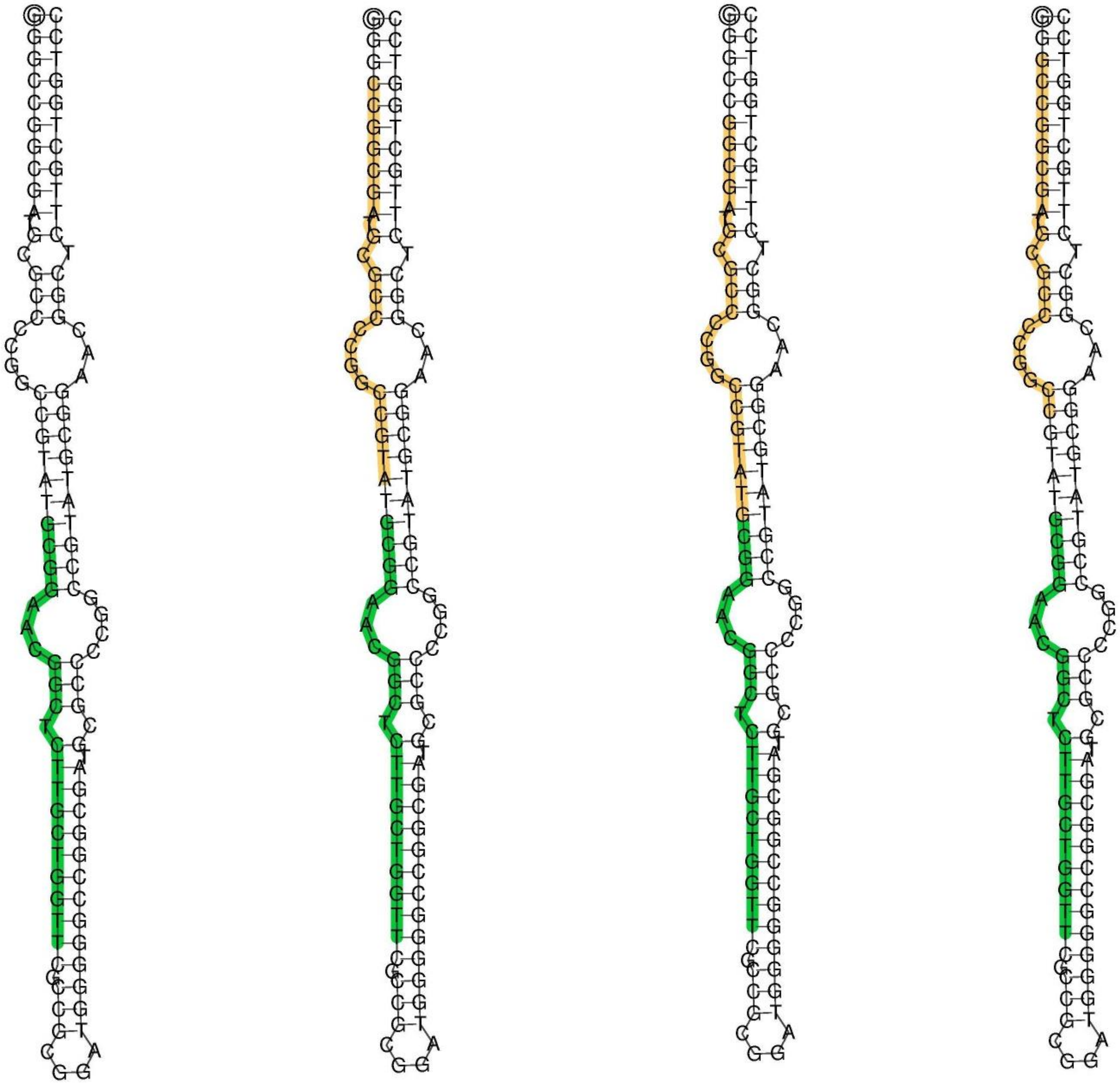

Hvu-new30

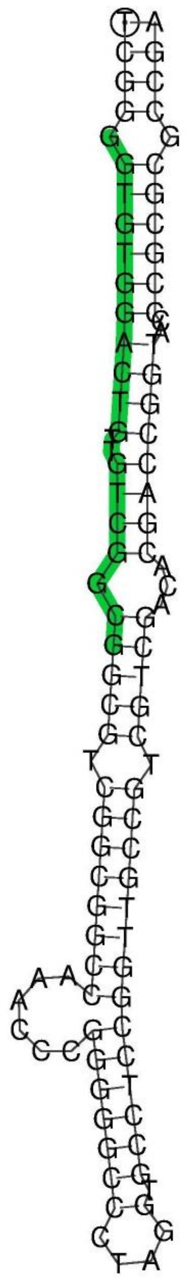

Hvu-new31

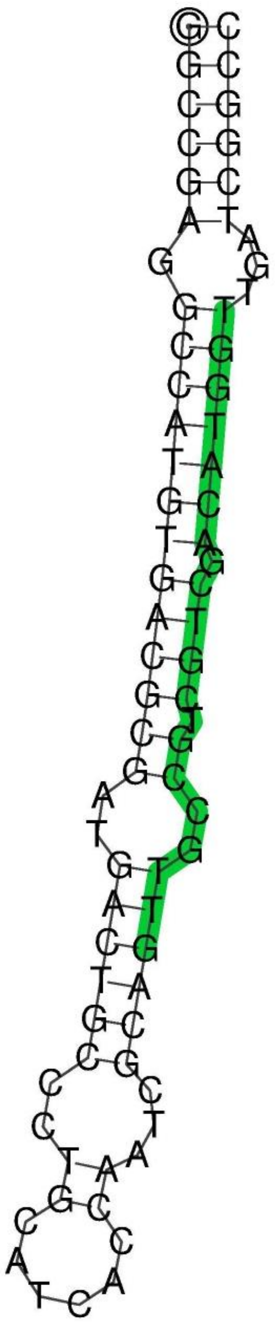

Hvu-new32

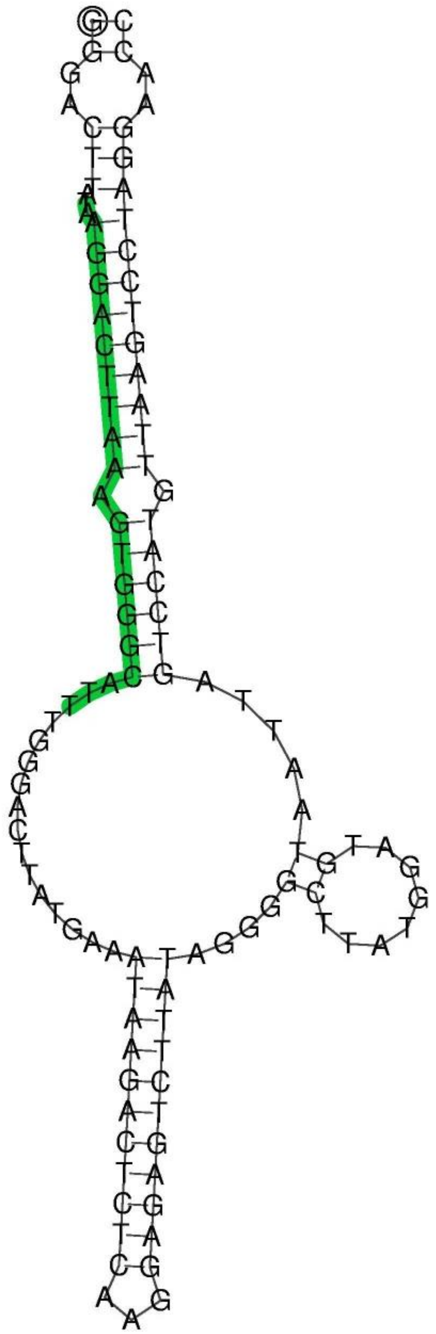

Hvu-new33

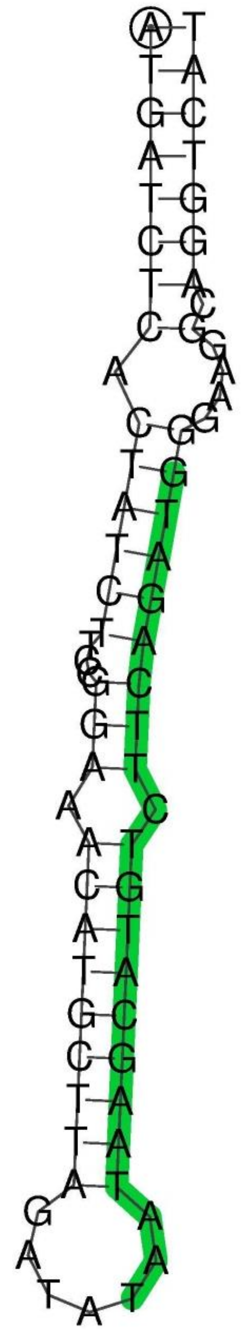

Hvu-new34

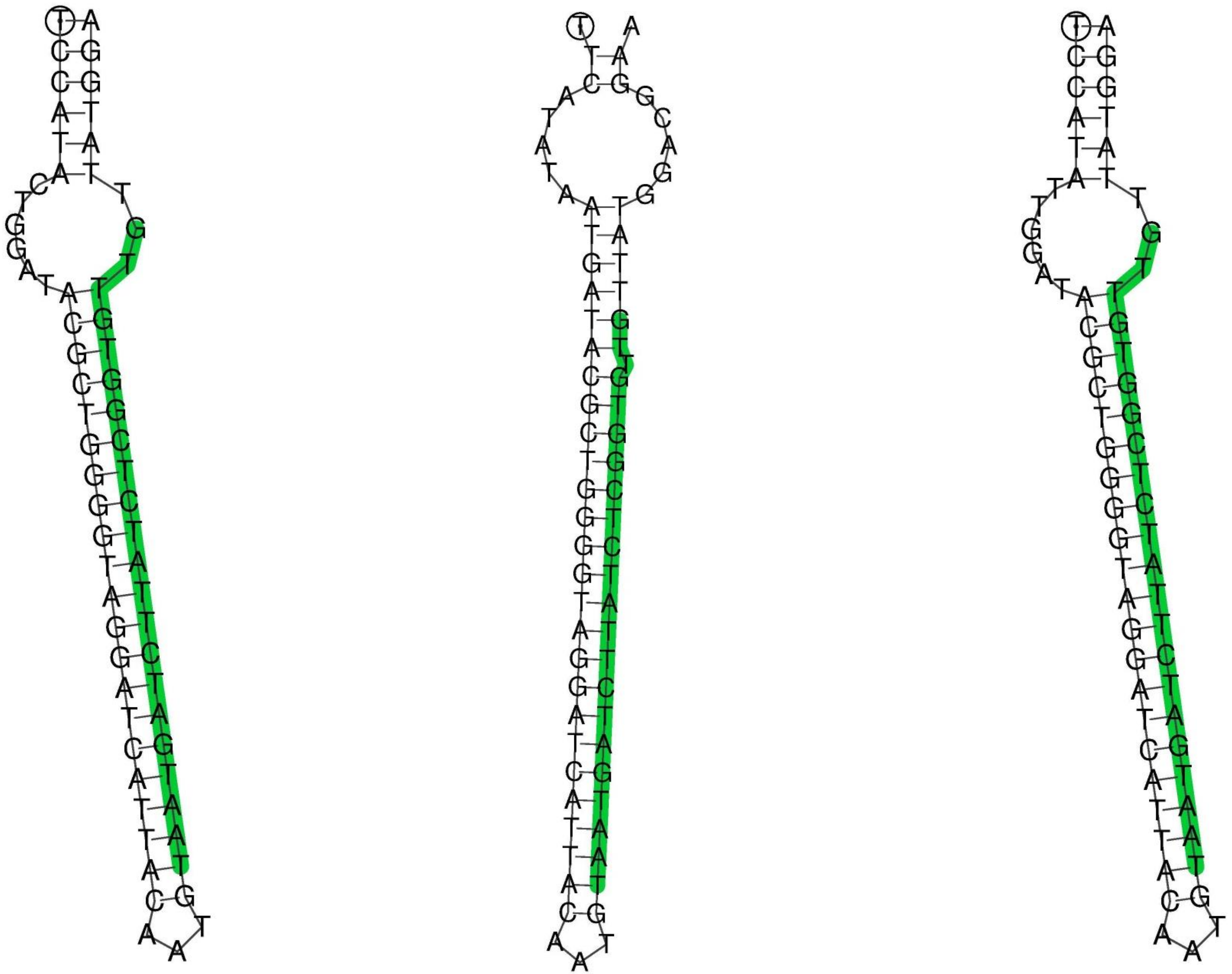

Hvu-new35

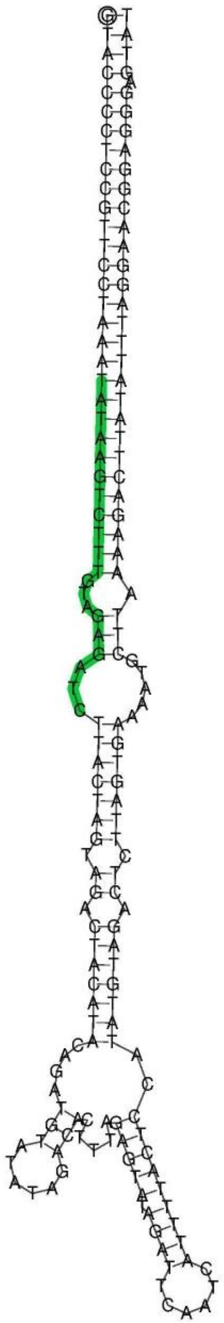

Hvu-new36

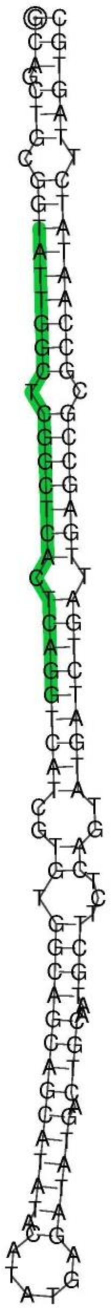

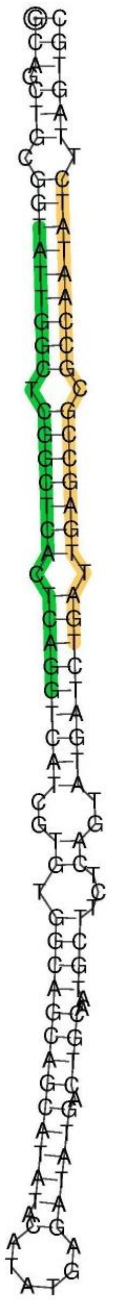

Hvu-new37

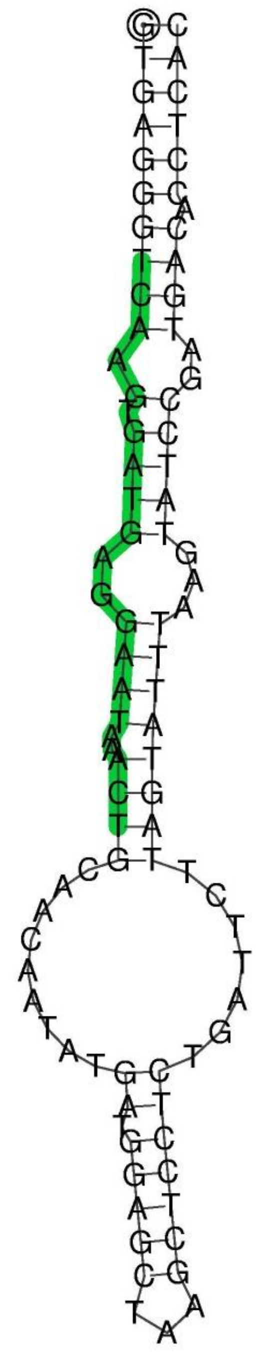

Hvu-new38

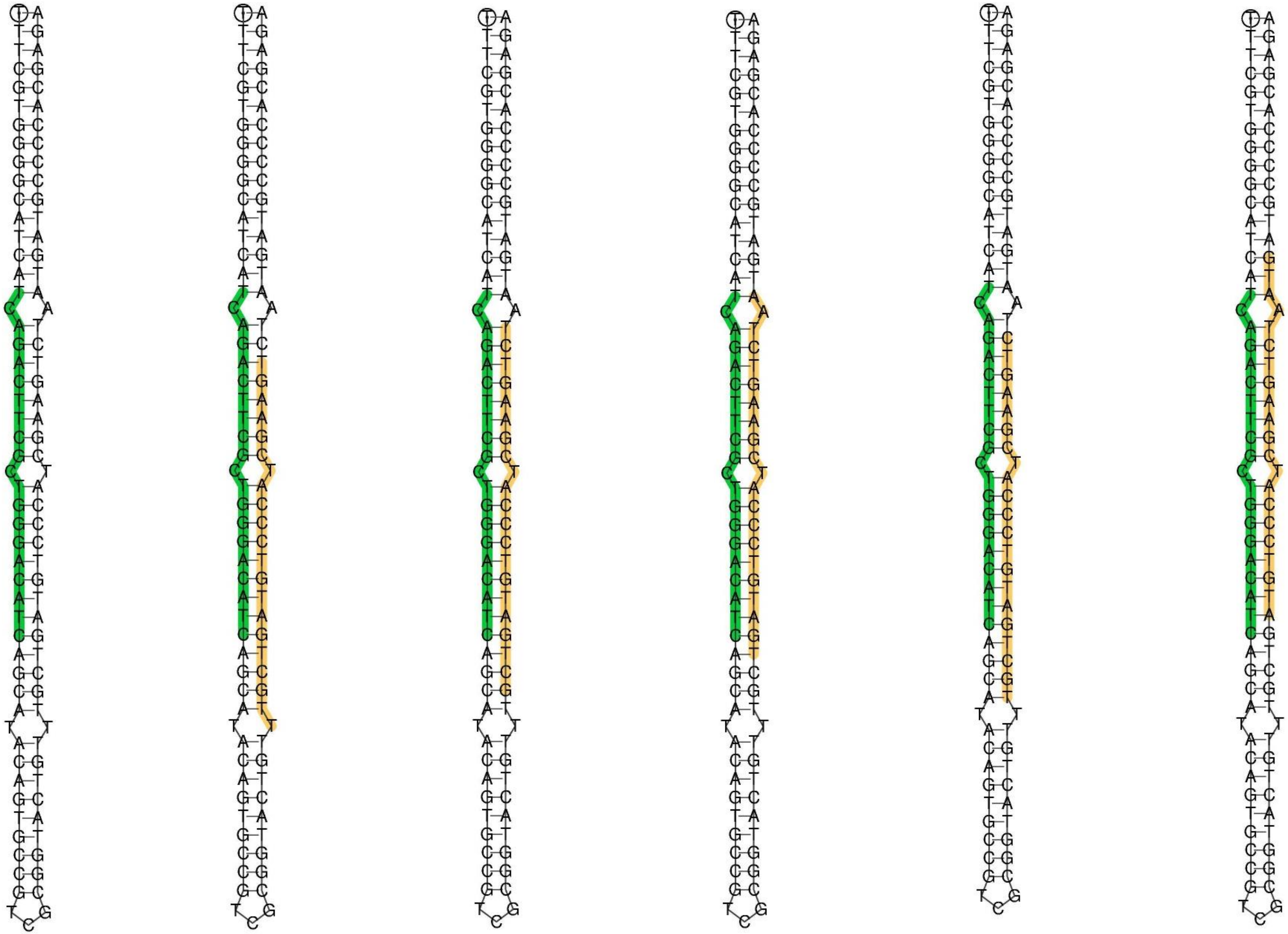

Hvu-new39

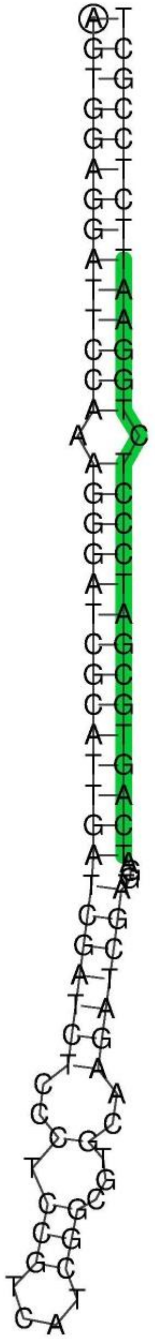

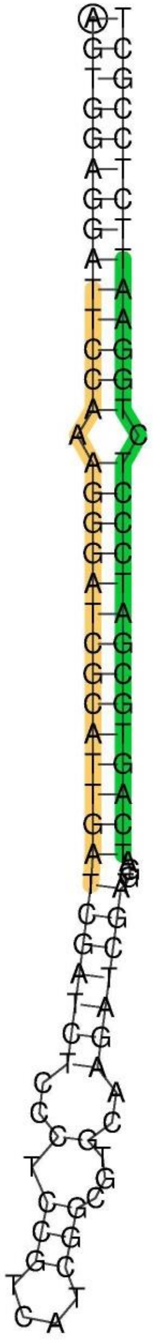

Hvu-new40

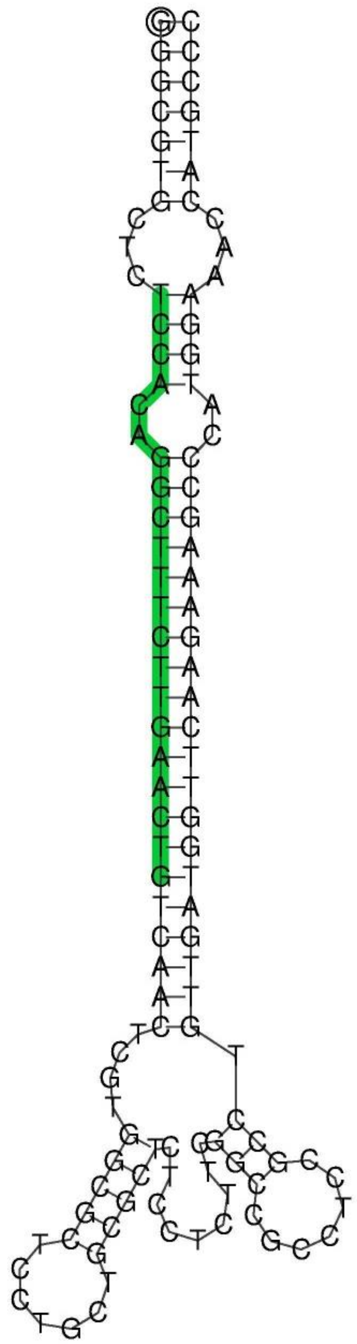

Hvu-new41

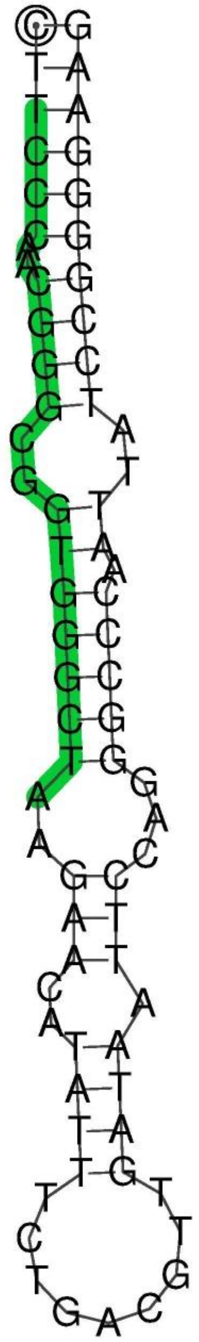

Hvu-new42

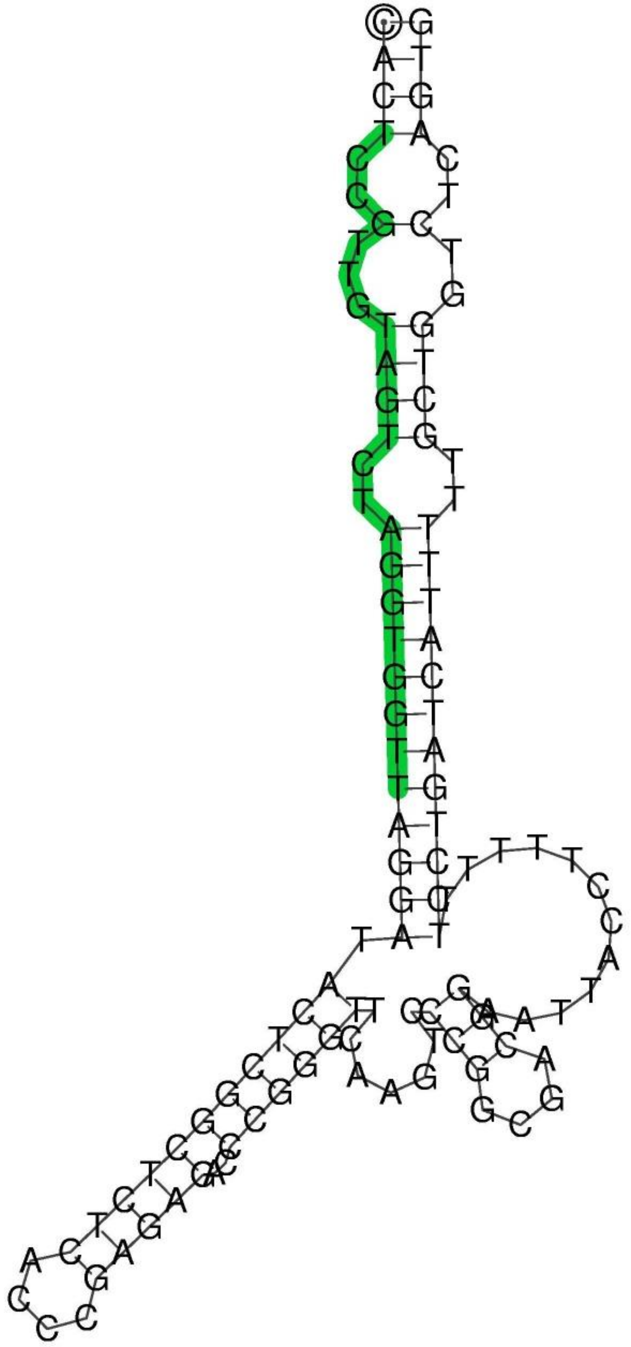

Hvu-new43

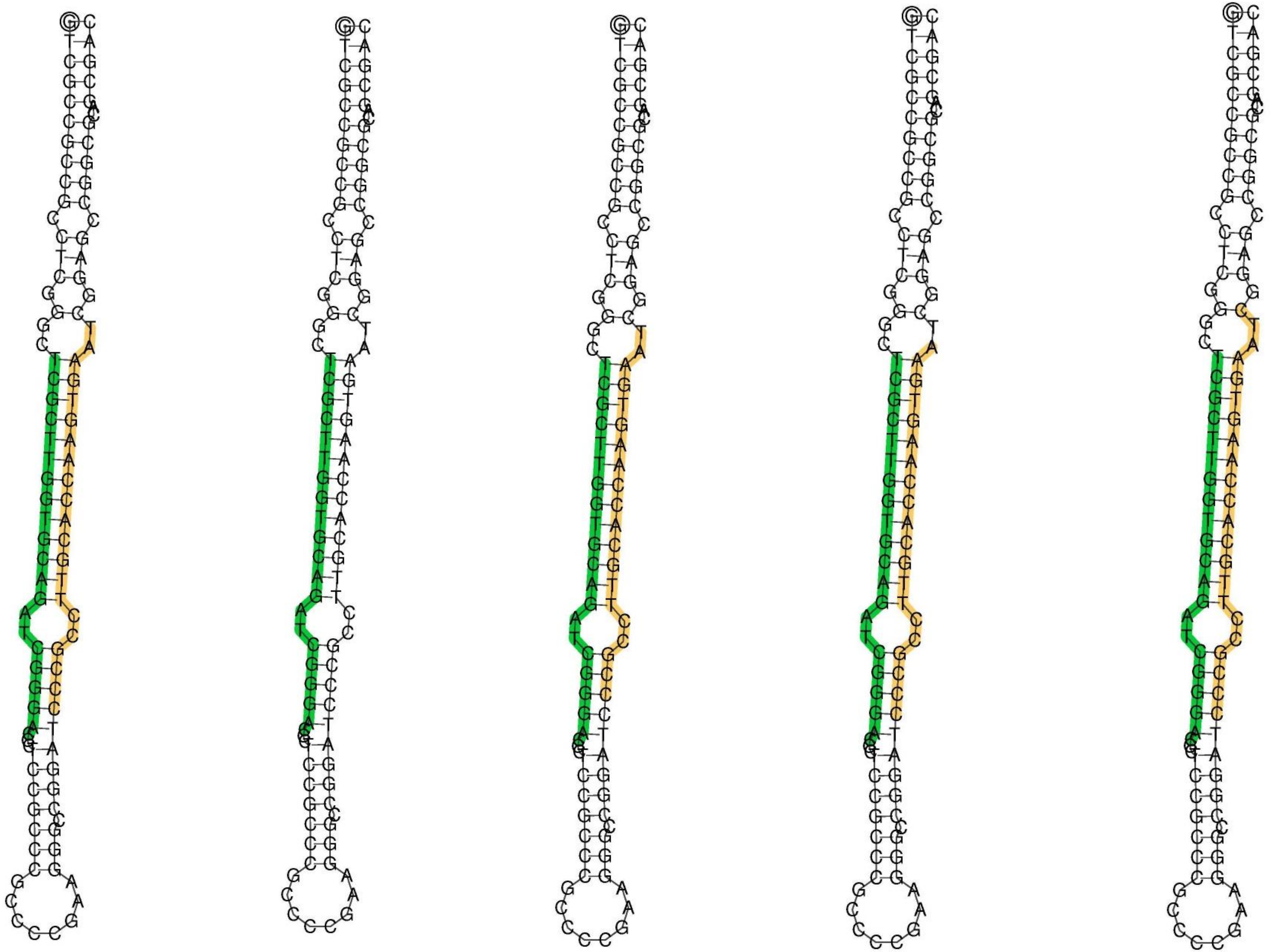

Hvu-new44

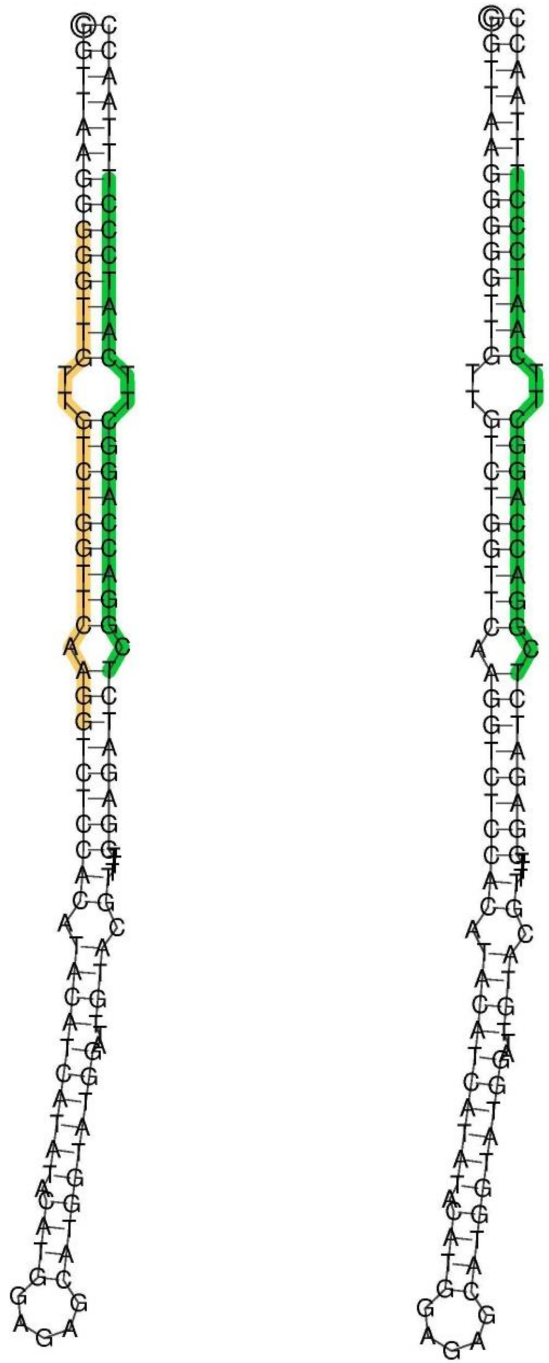

Hvu-new45

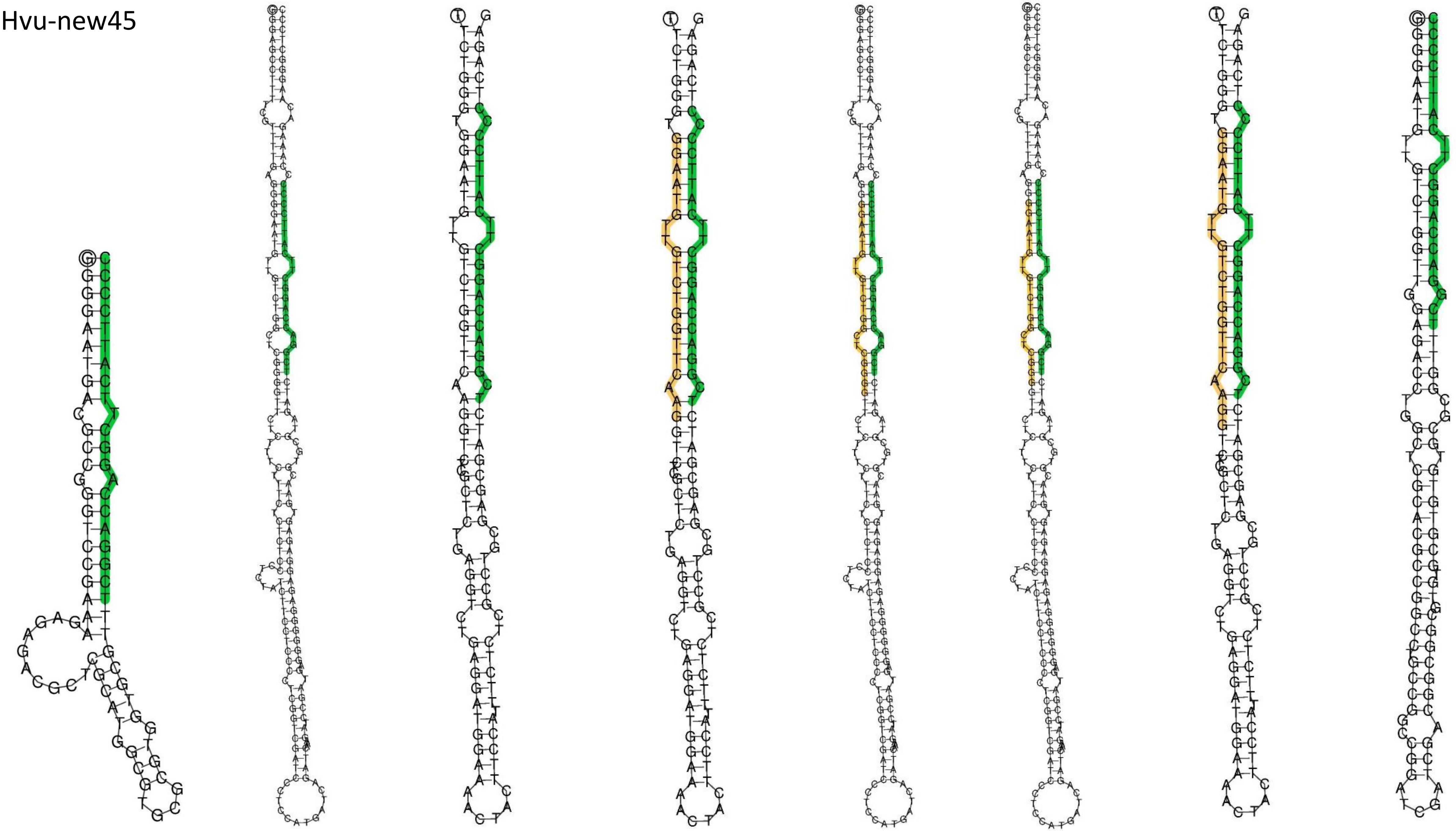

Hvu-new46

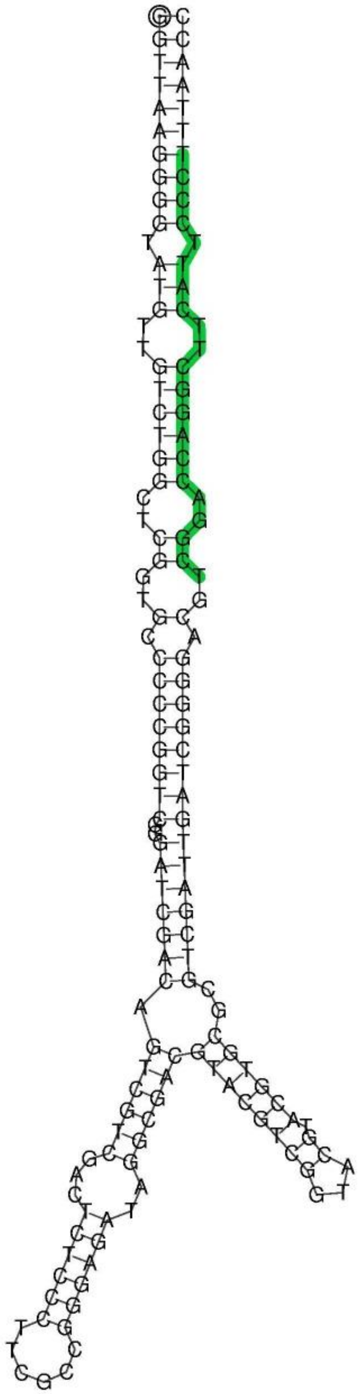

Hvu-new47

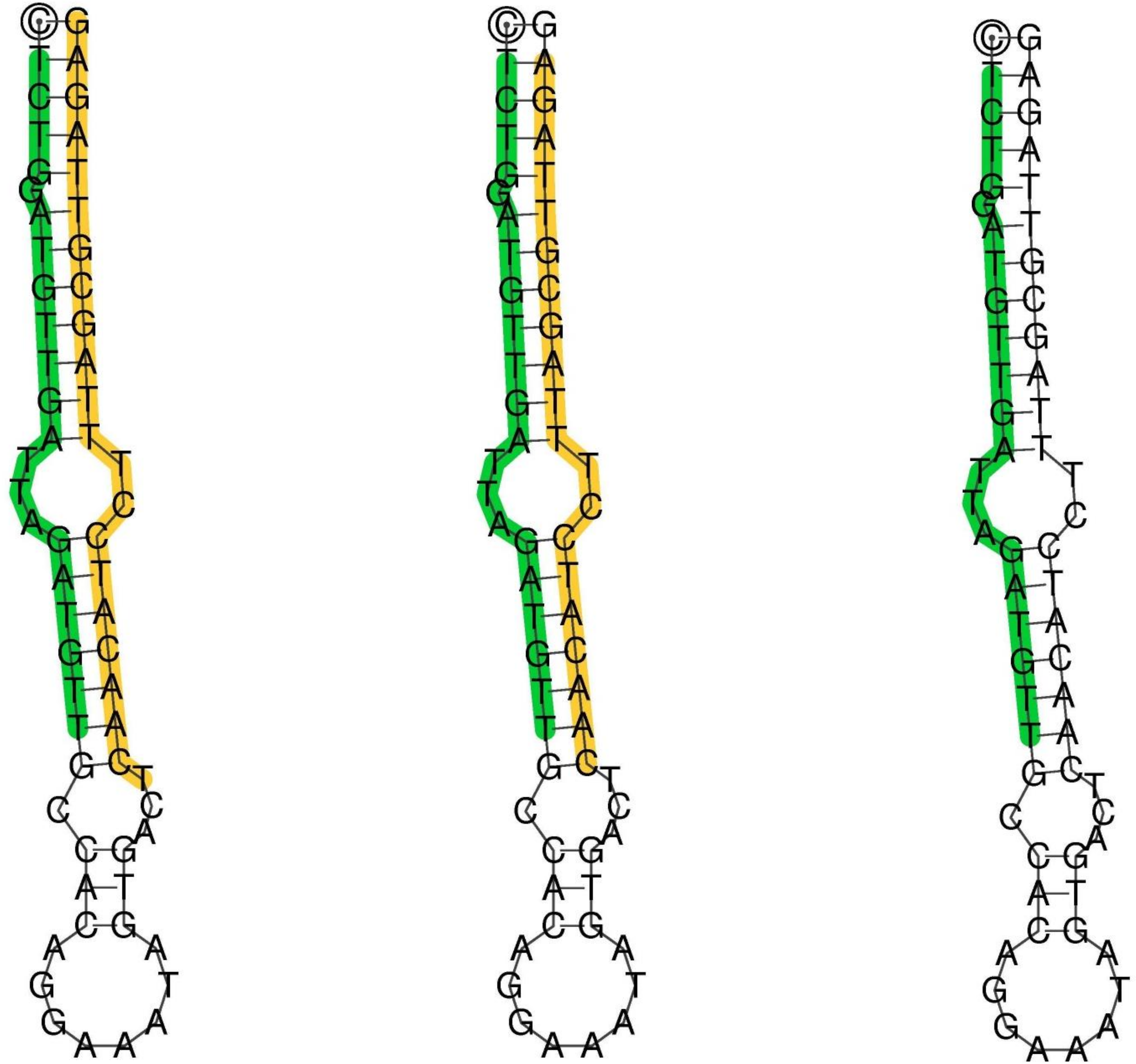

Hvu-new48

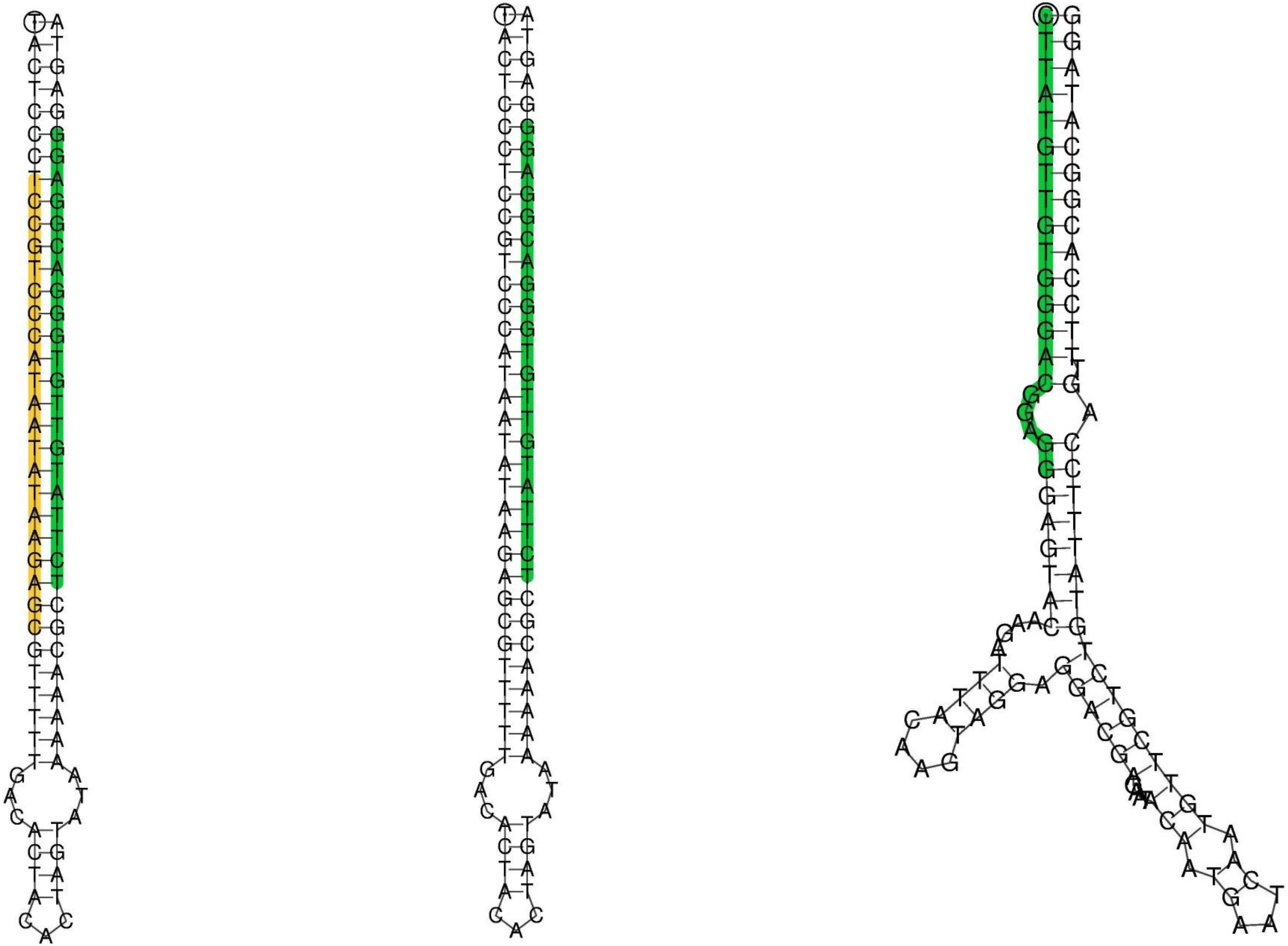

Hvu-new49

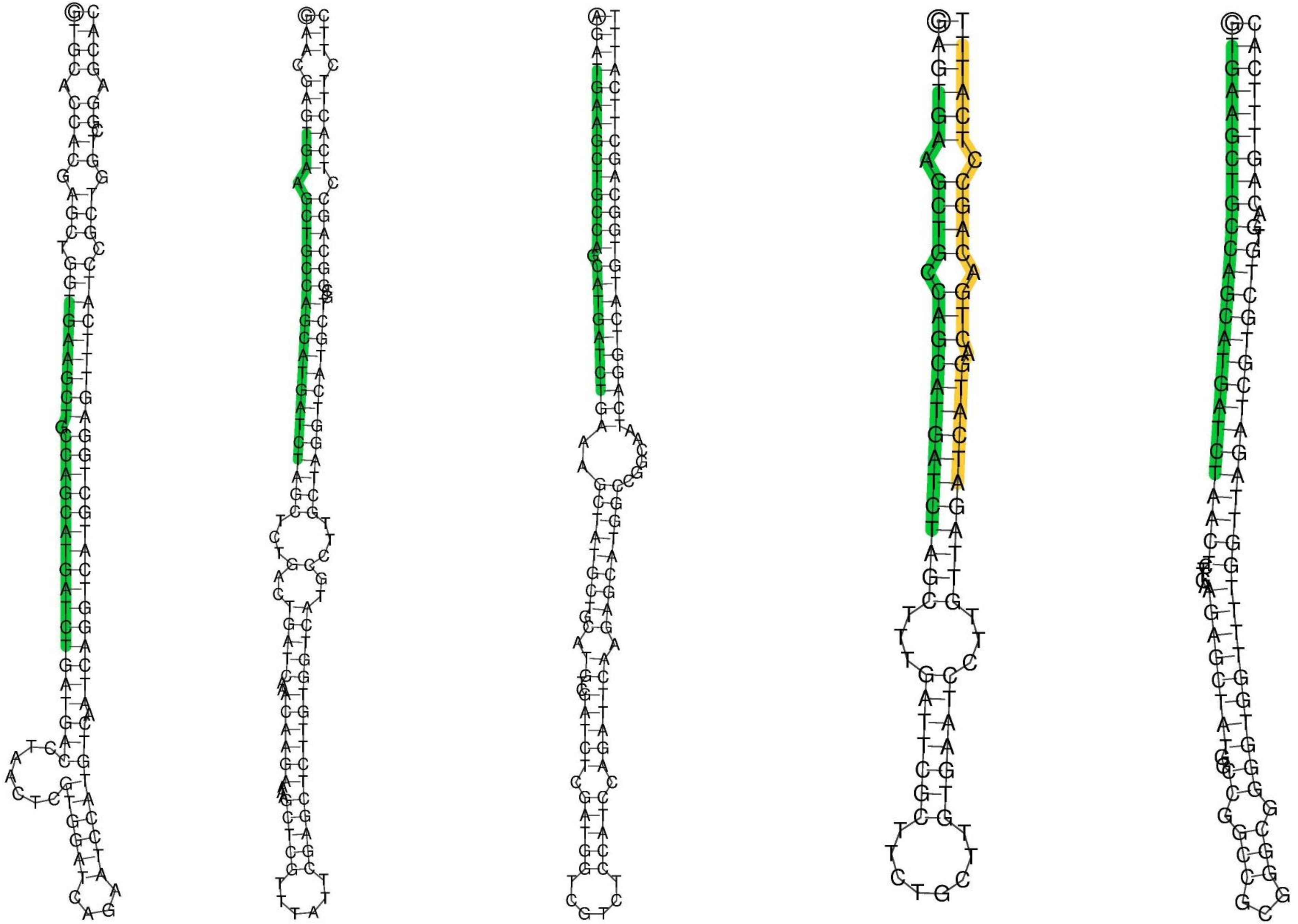

Hvu-new49

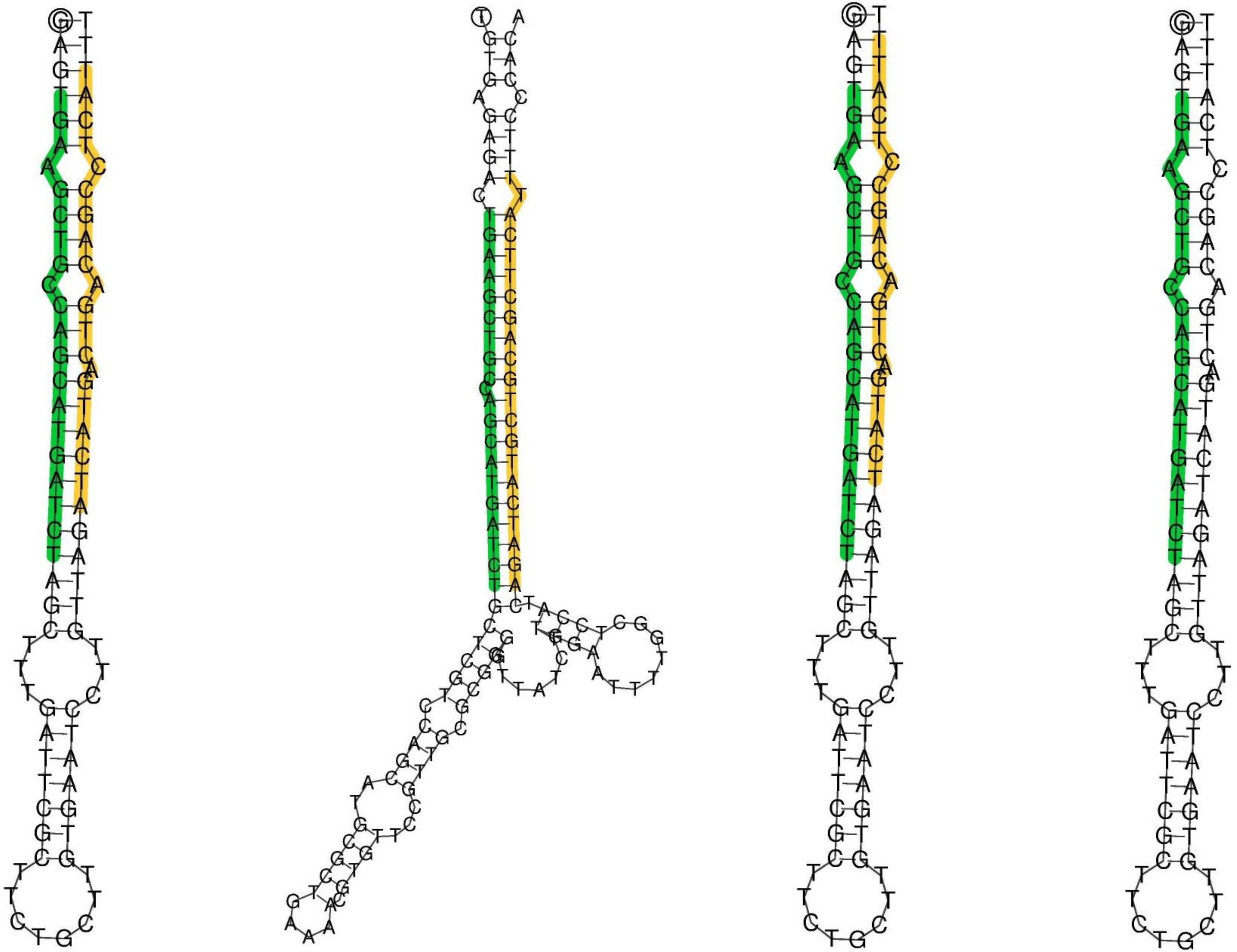

Hvu-new5[illegible]

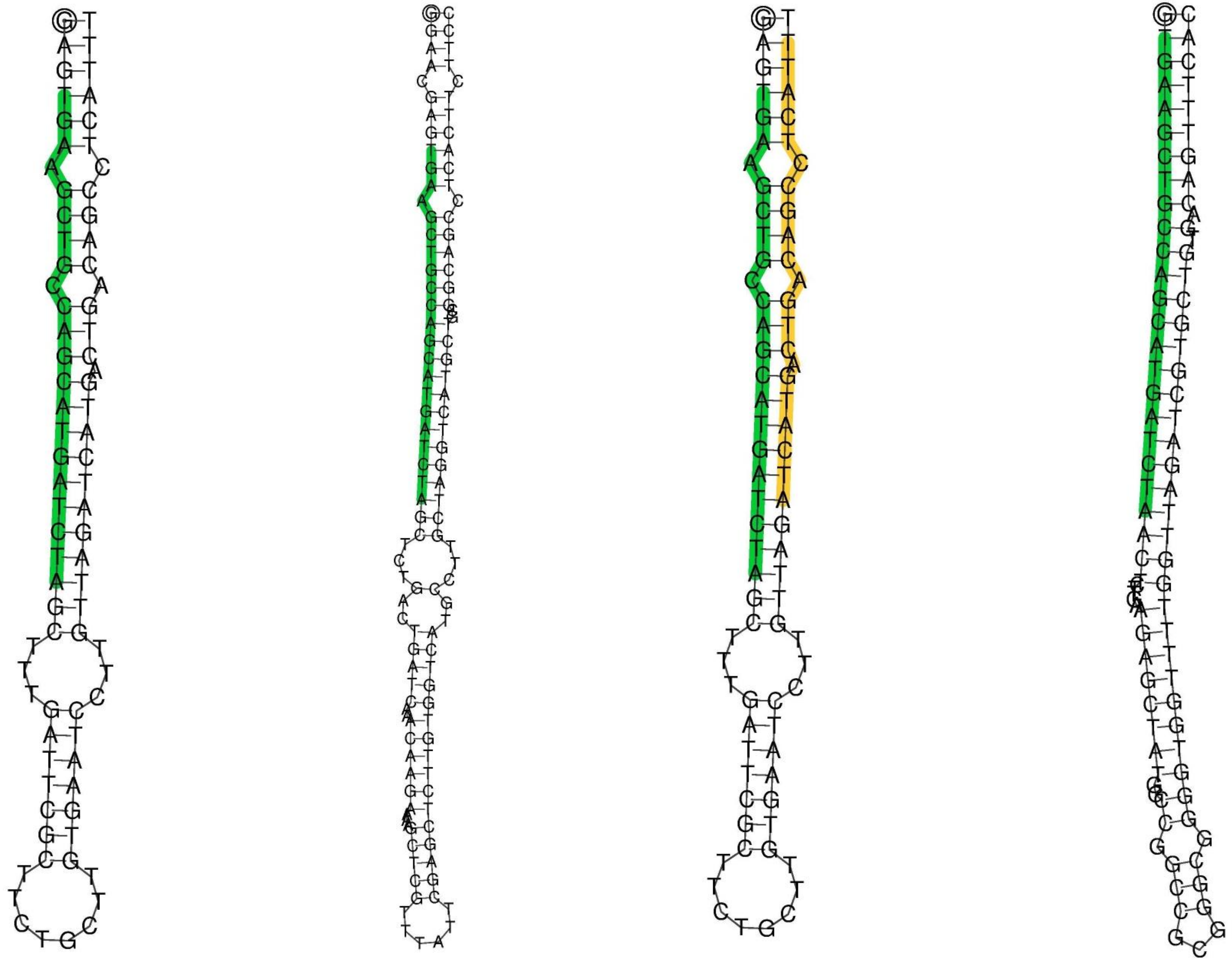

Hvu-new51

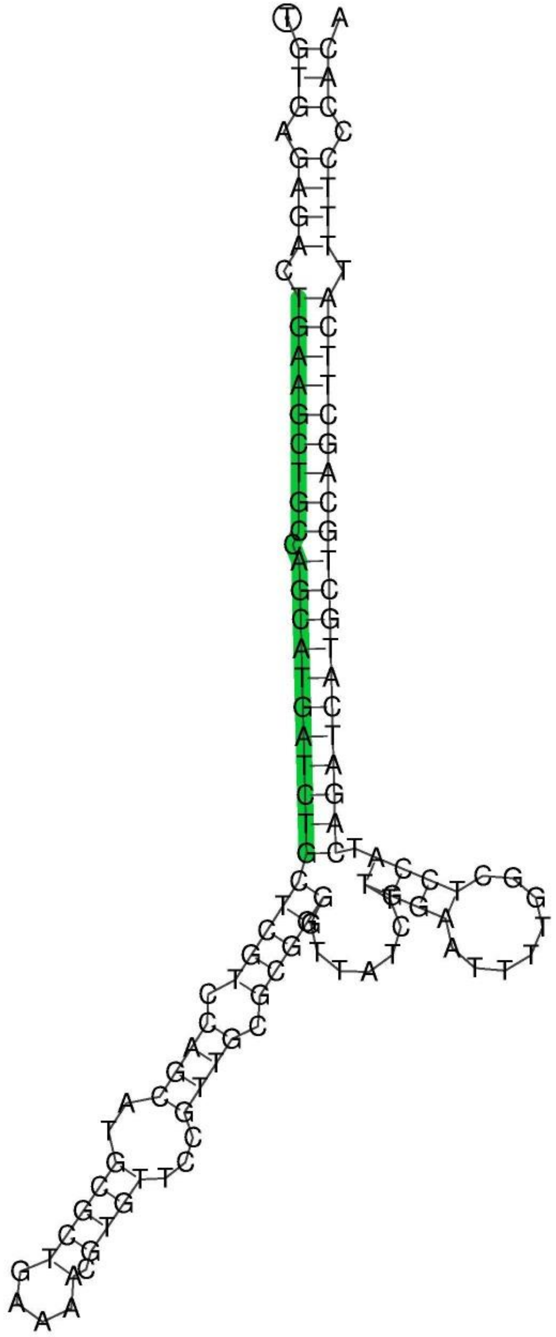

Hvu-new52

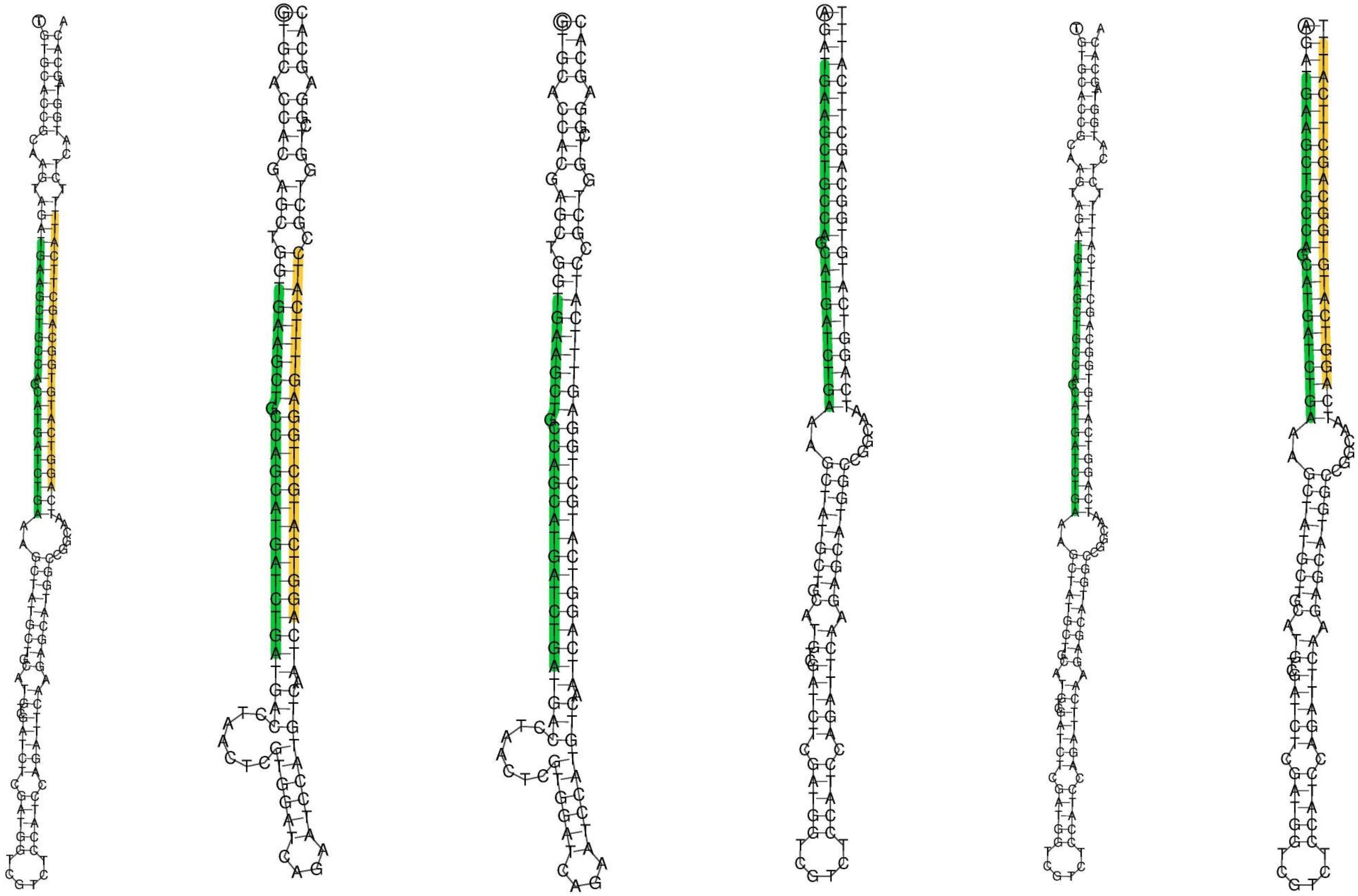

Hvu-new53

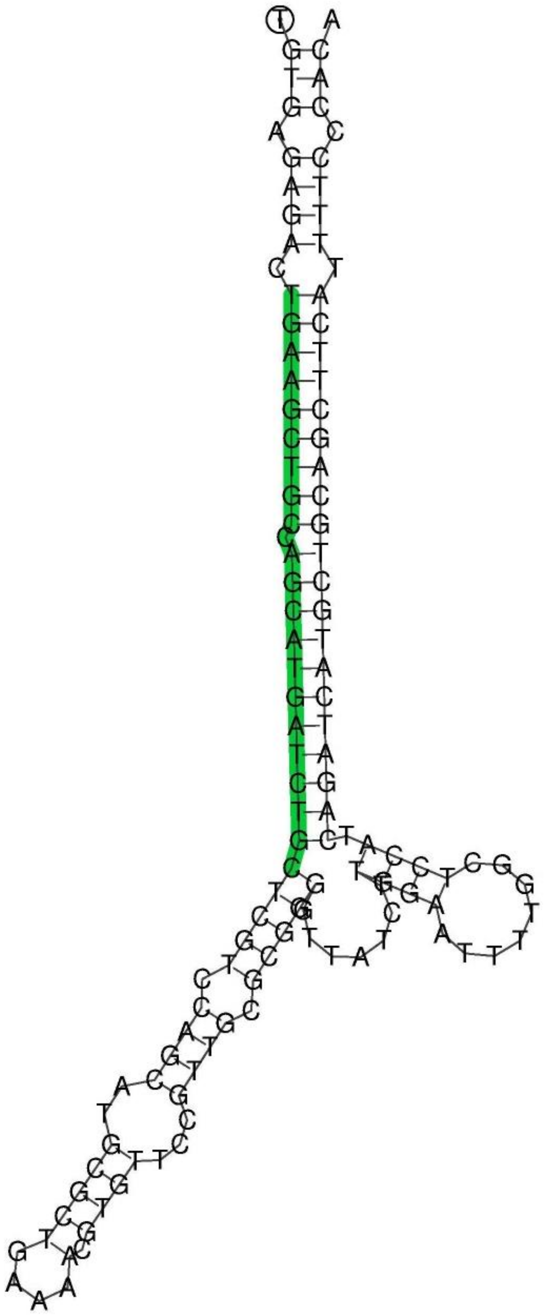

Hvu-new54

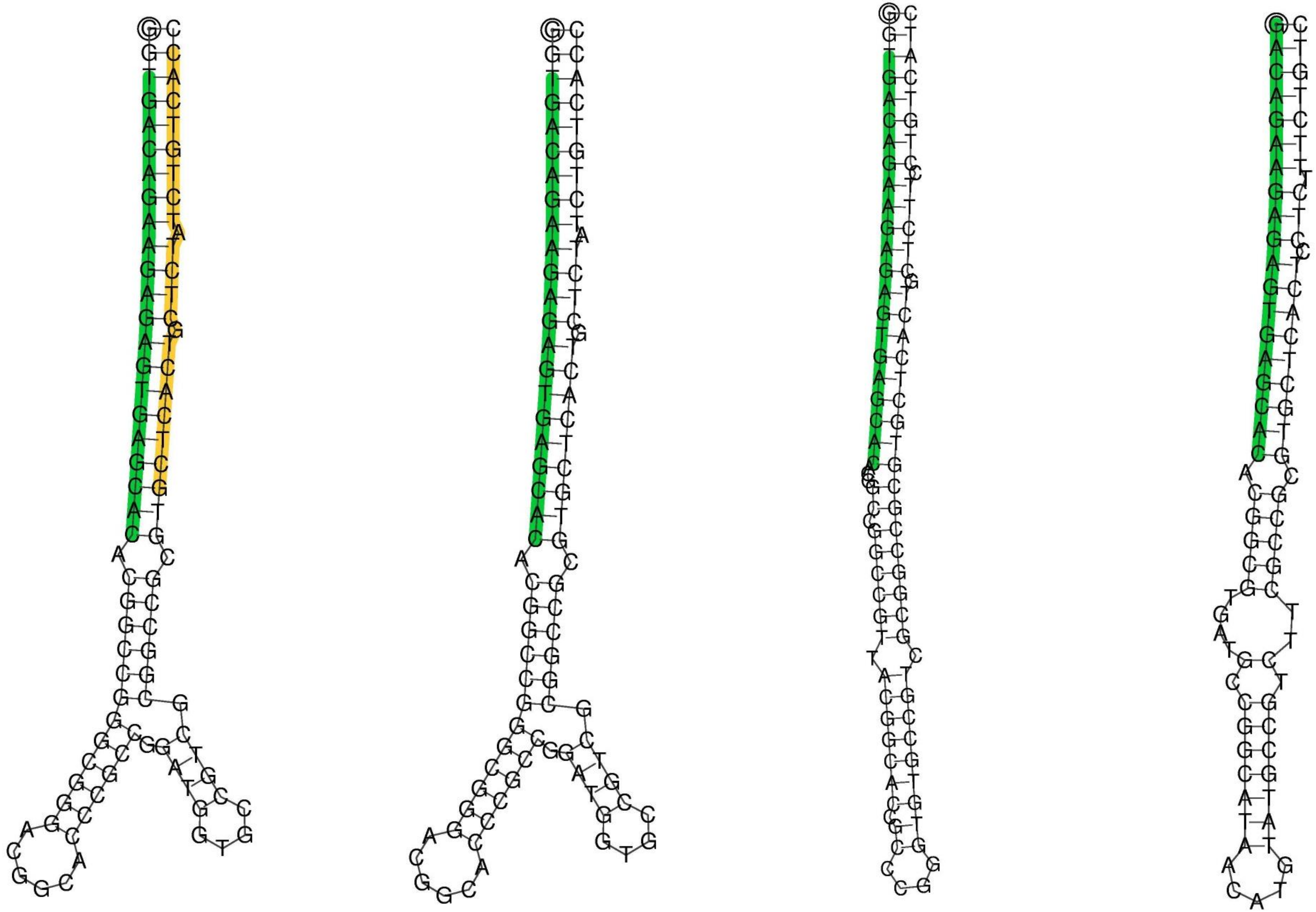

Hvu-new55

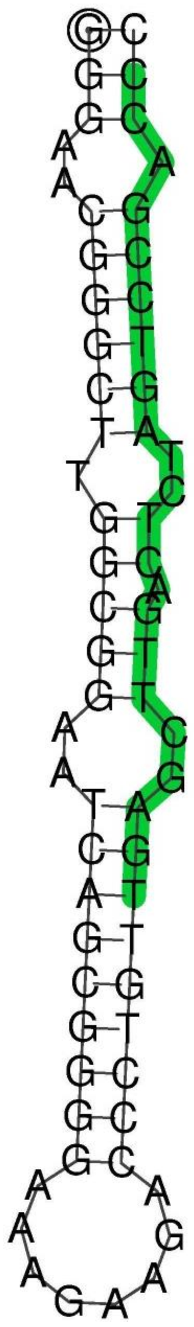

Hvu-new56

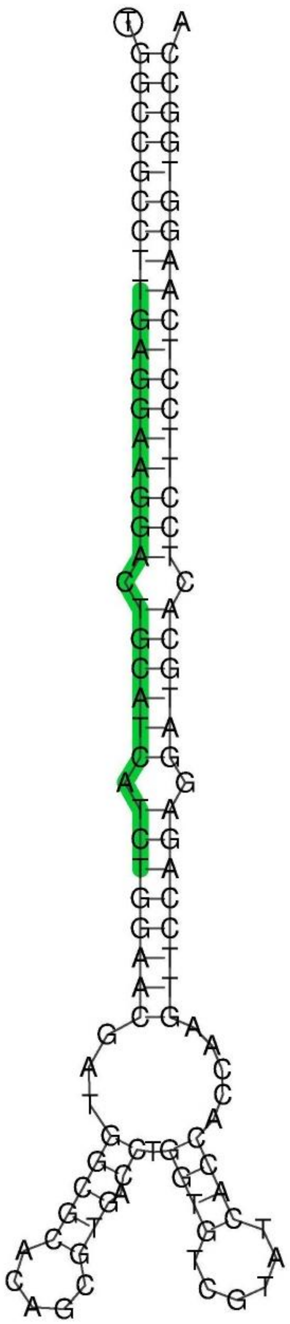

Hvu-new57

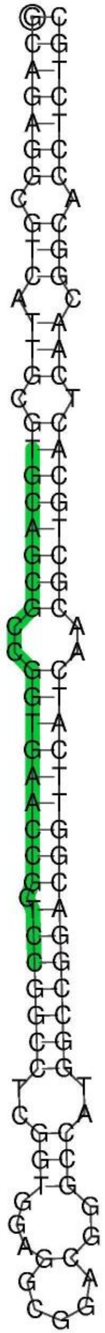

Hvu-new58

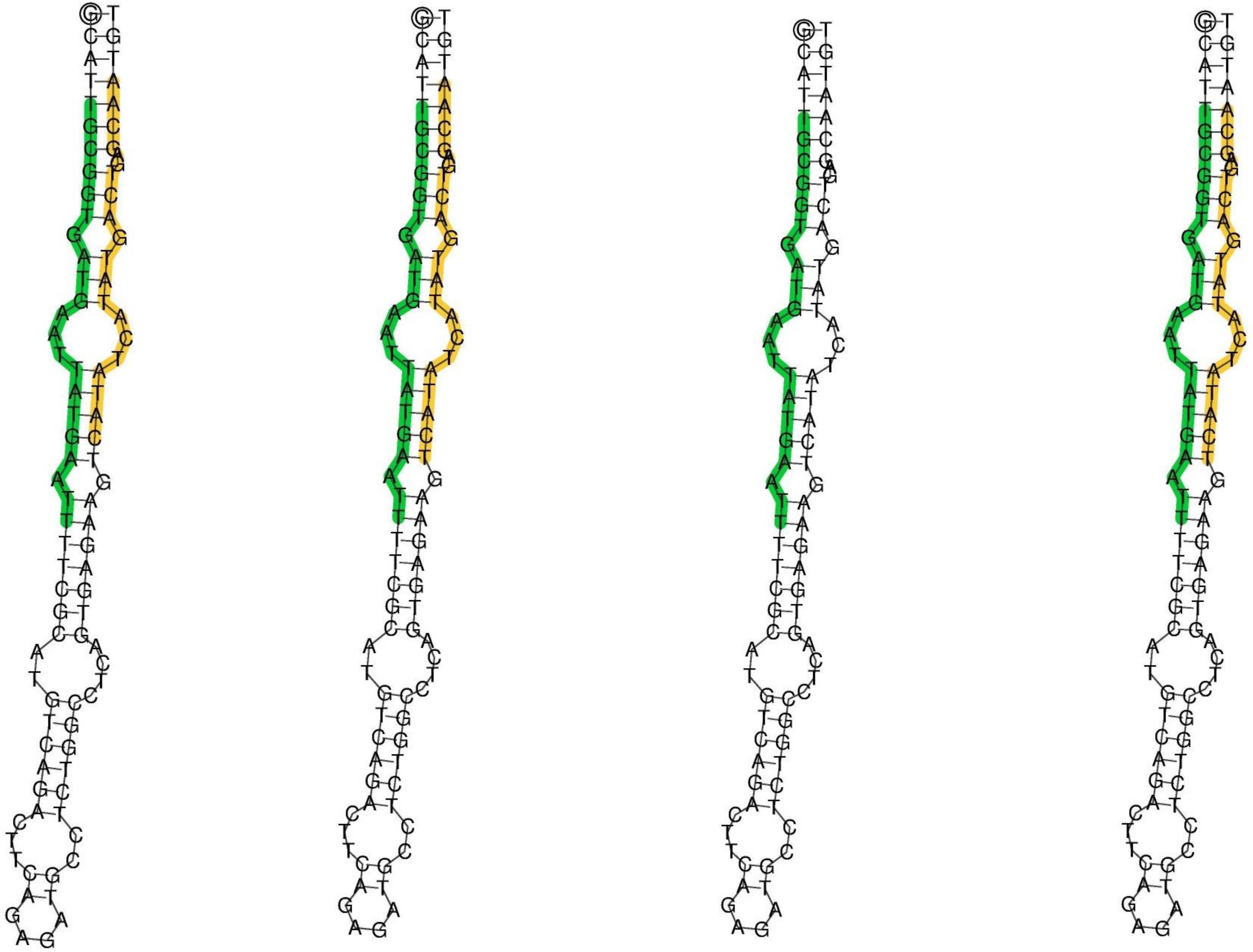

Hvu-new59

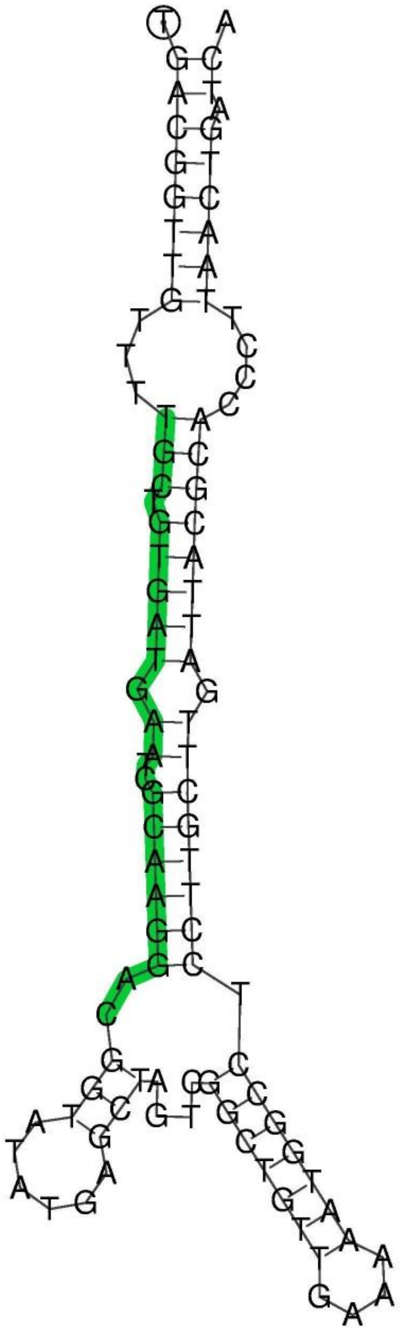

Hvu-new60

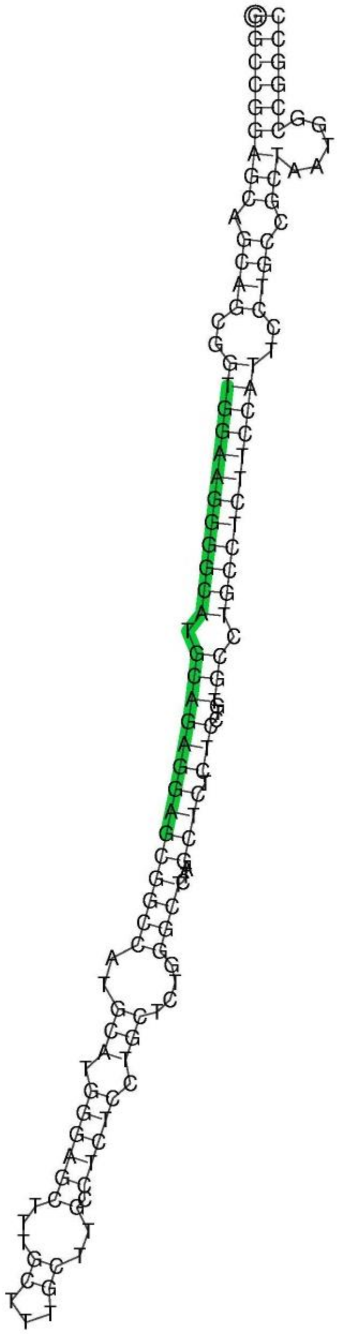

Hvu-new61

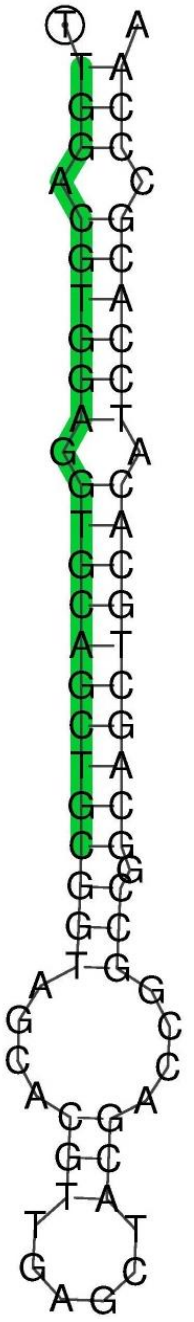

Hvu-new62

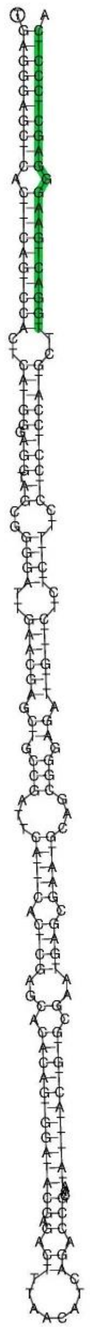

Hvu-new63

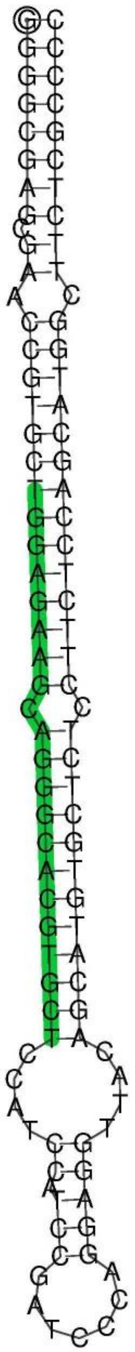

Hvu-new64

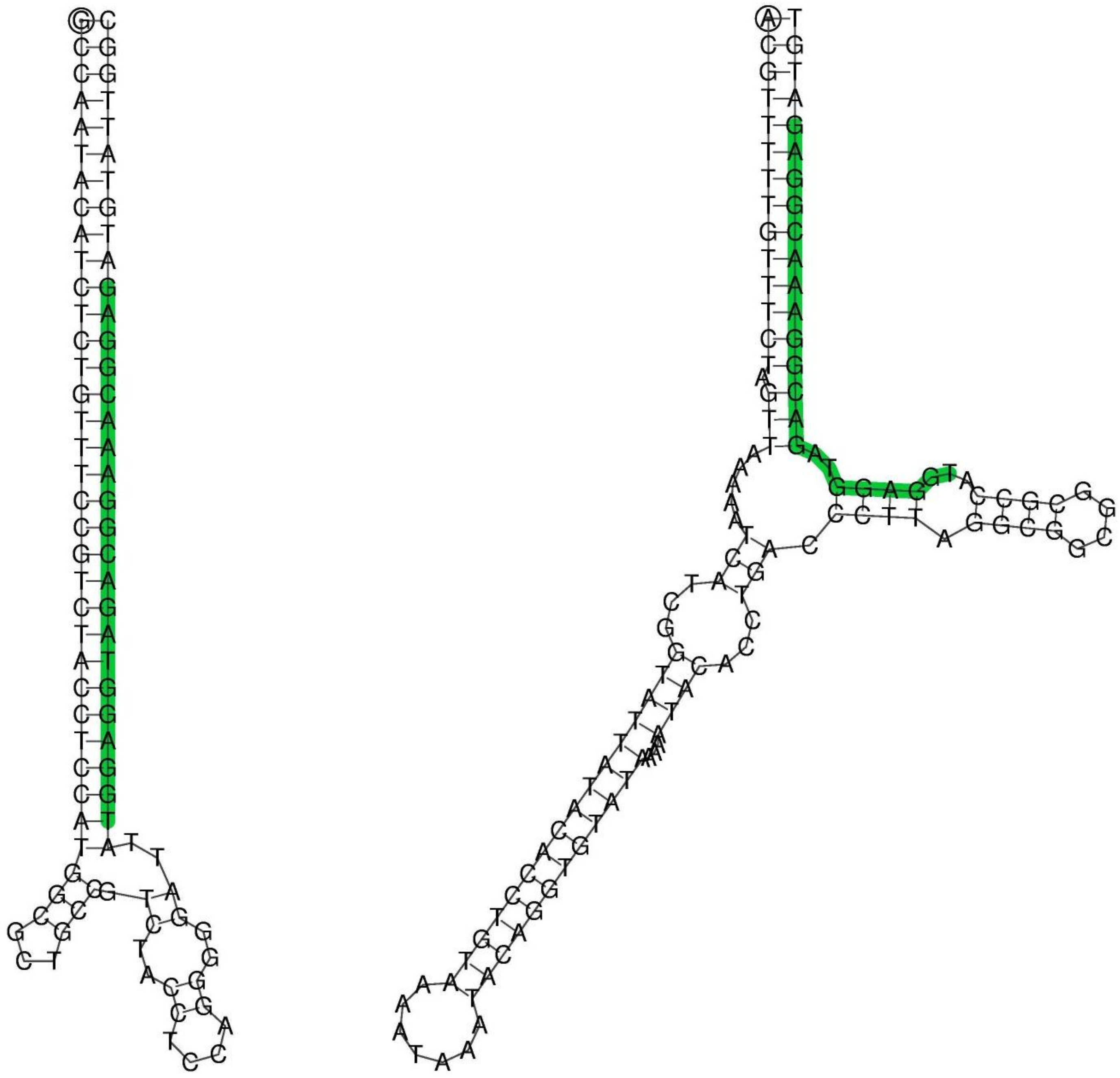

Hvu-new65

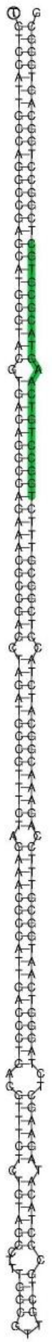

Hvu-new66

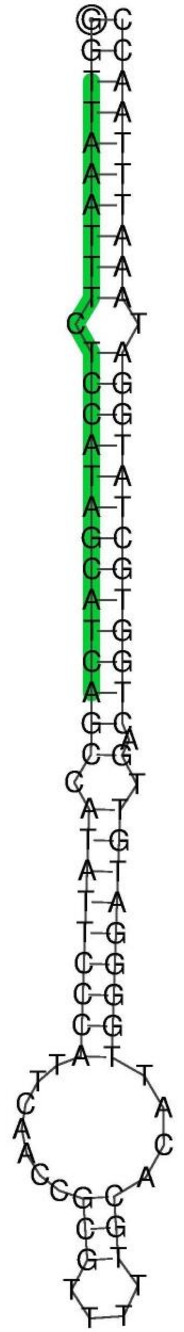

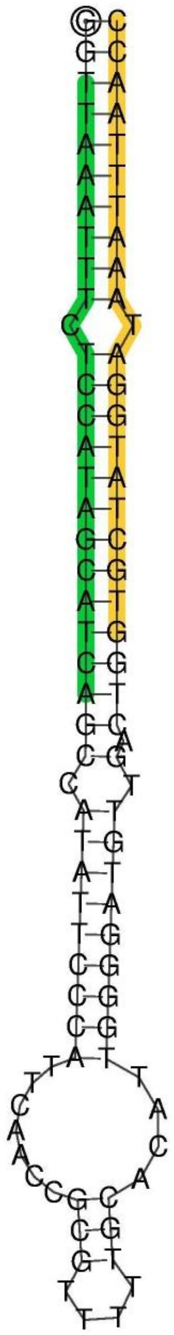

Hvu-new67

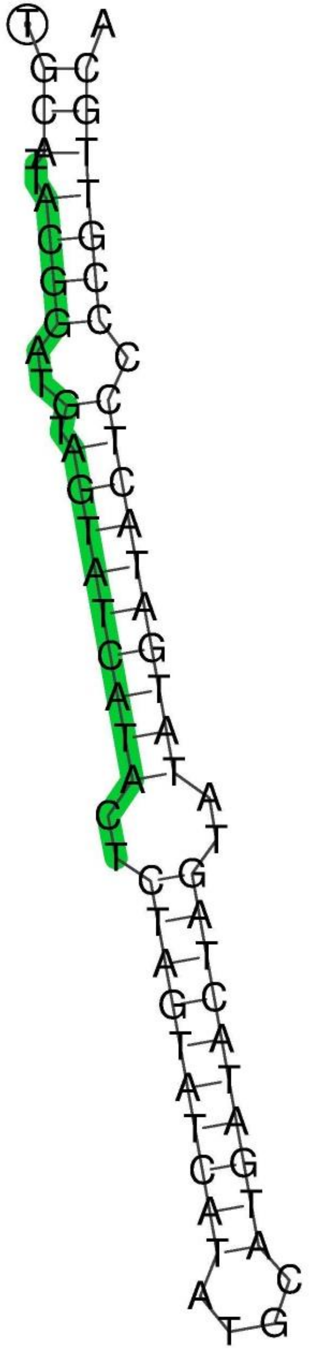

Hvu-new68

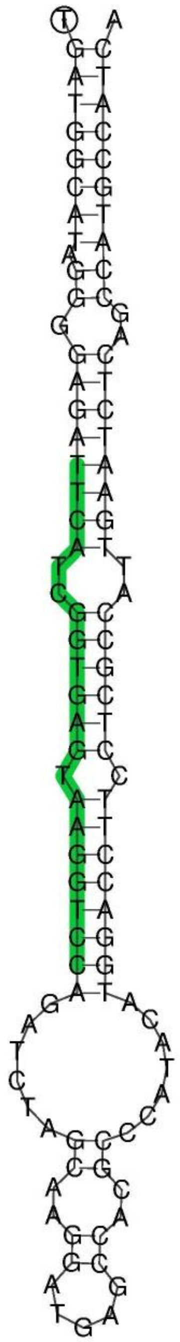

Hvu-new69

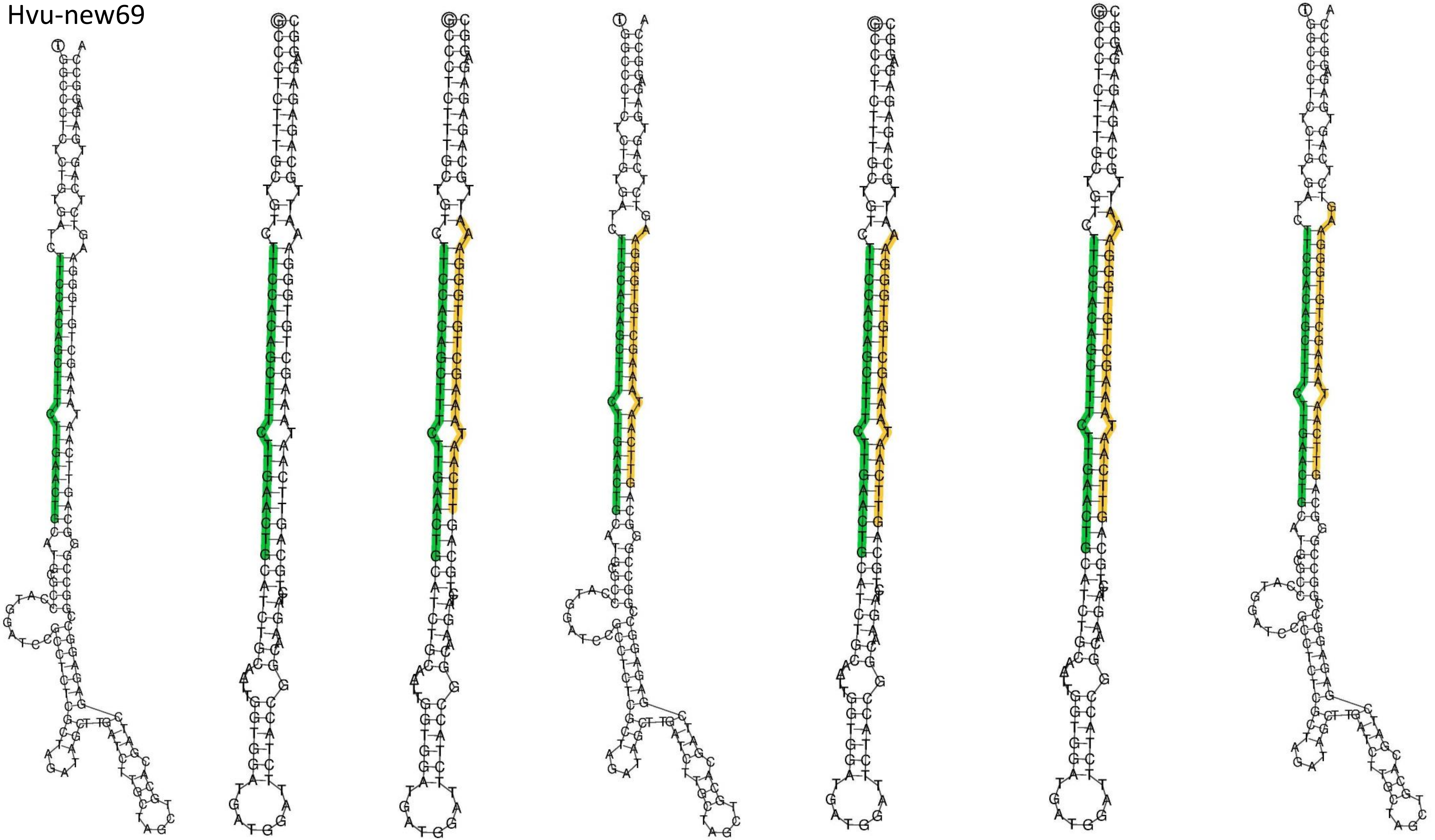

Hvu-new70

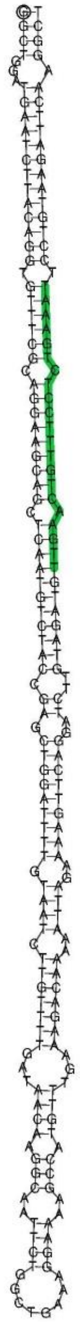

Hvu-new71

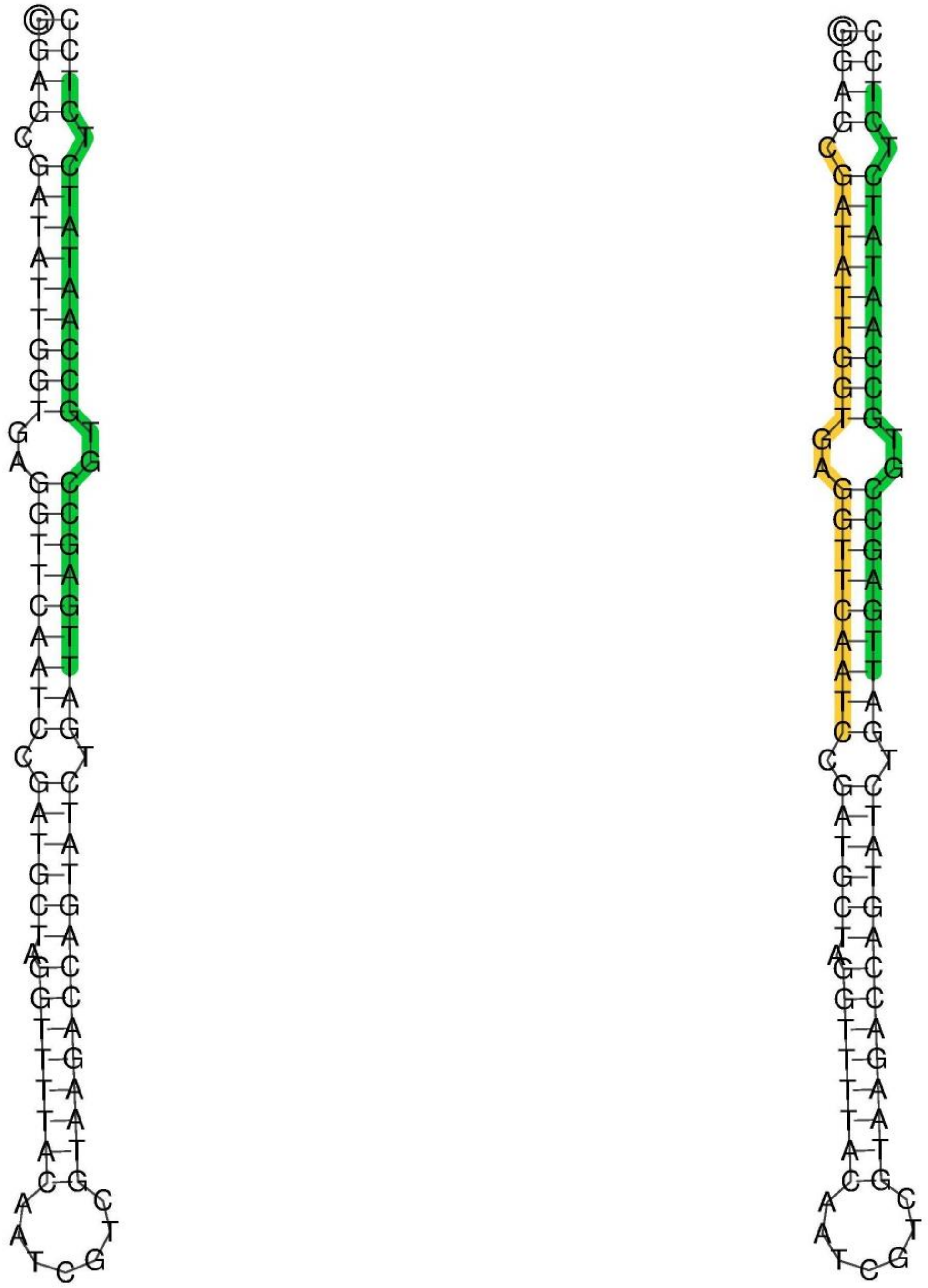

Hvu-new72

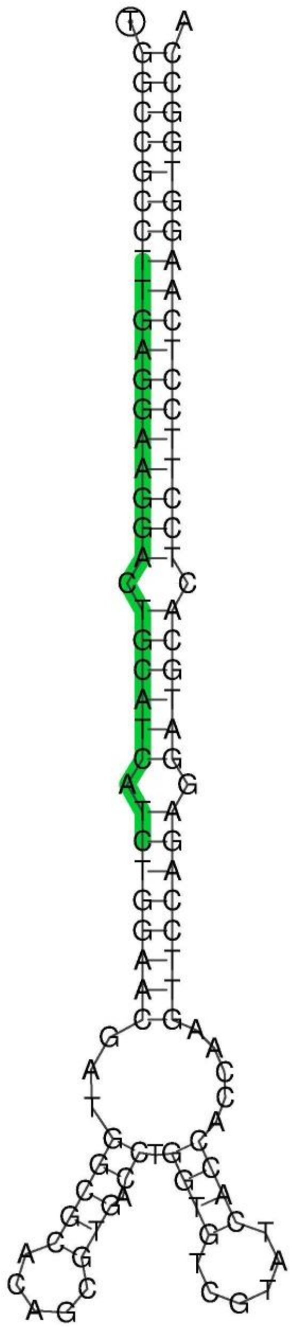

Hvu-new73

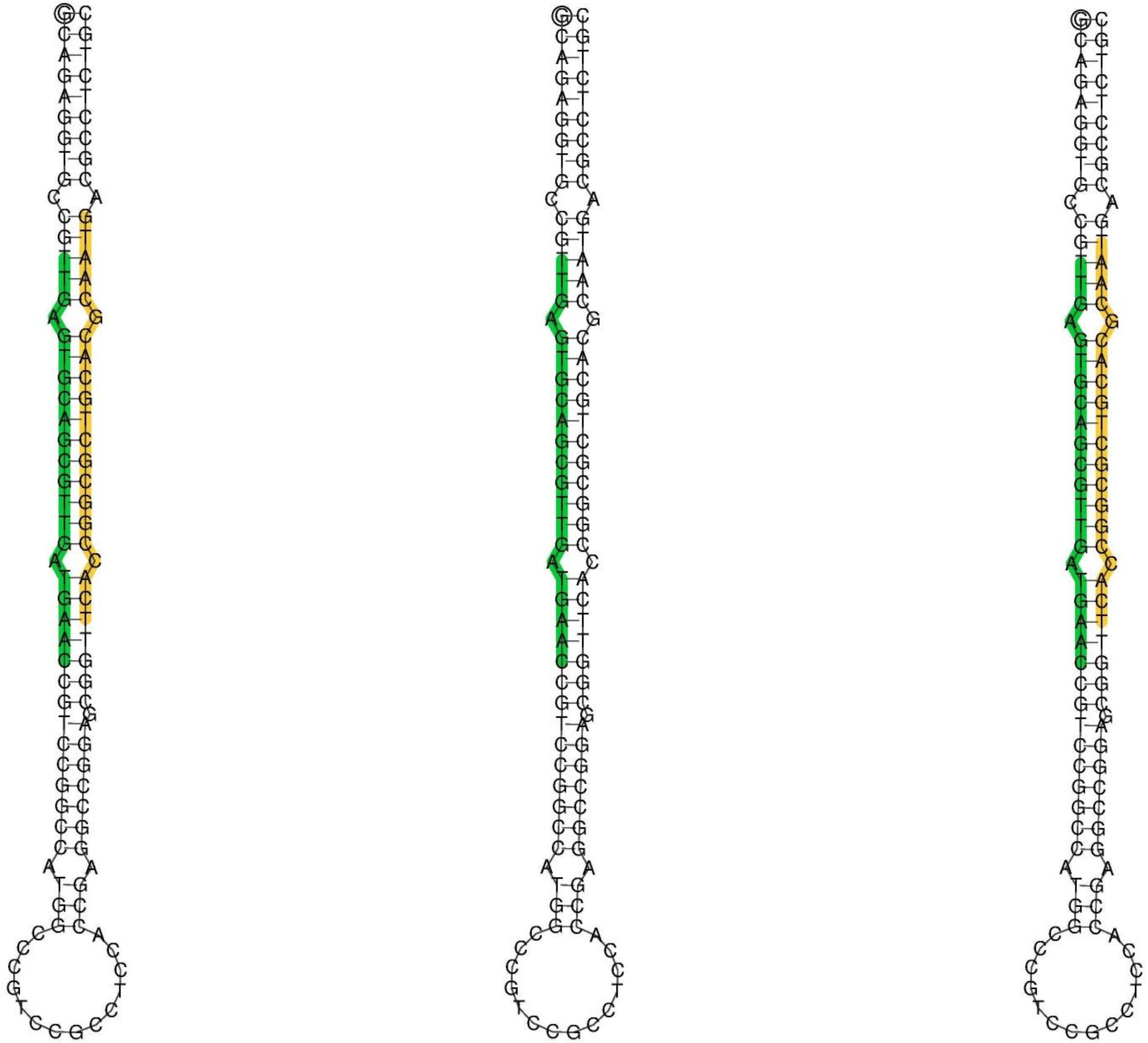

Hvu-new74

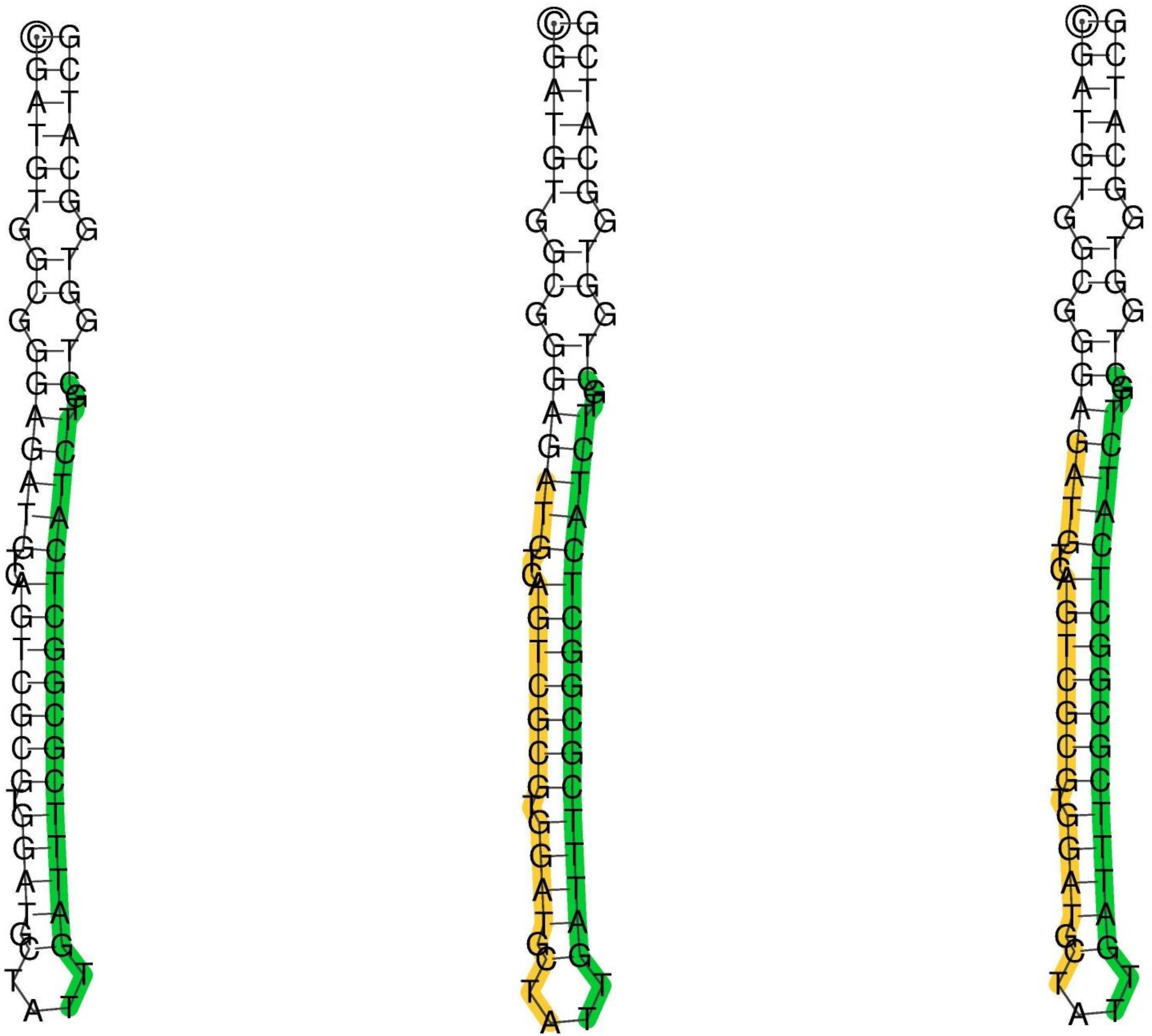

Hvu-new75

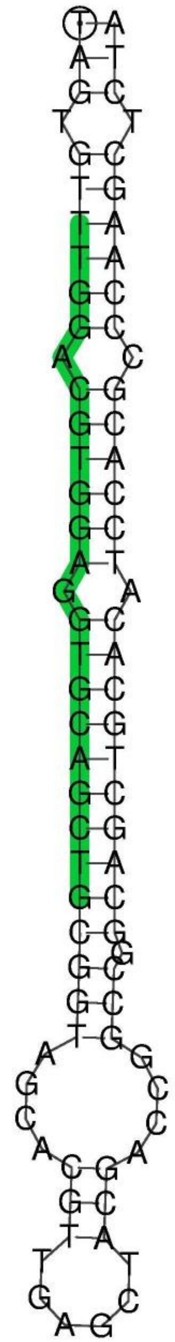

Hvu-new76

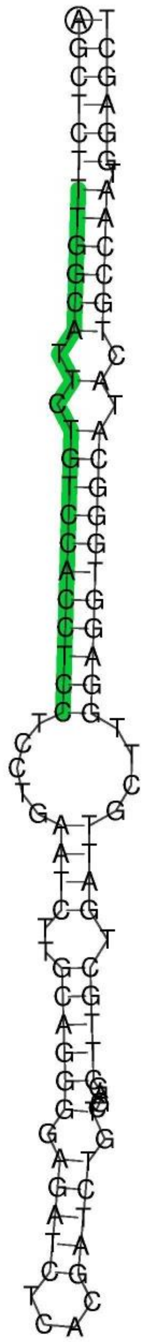

Hvu-new77

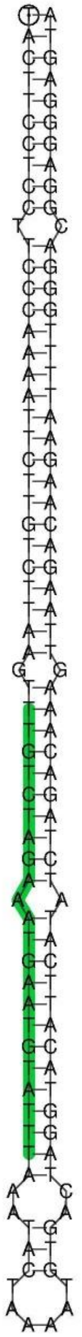

Hvu-new78

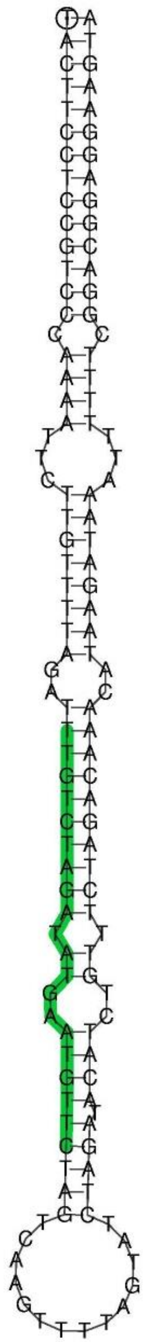

Hvu-new79

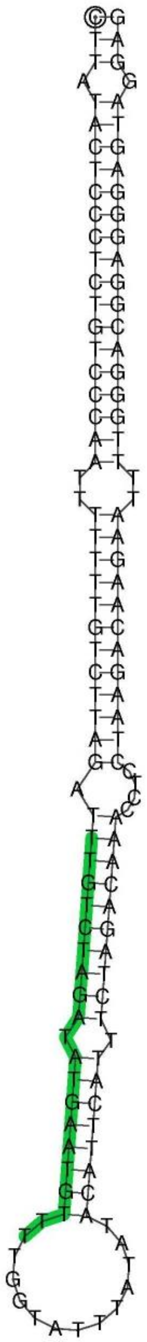

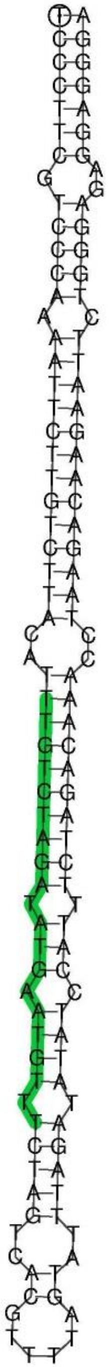

Hvu-new80

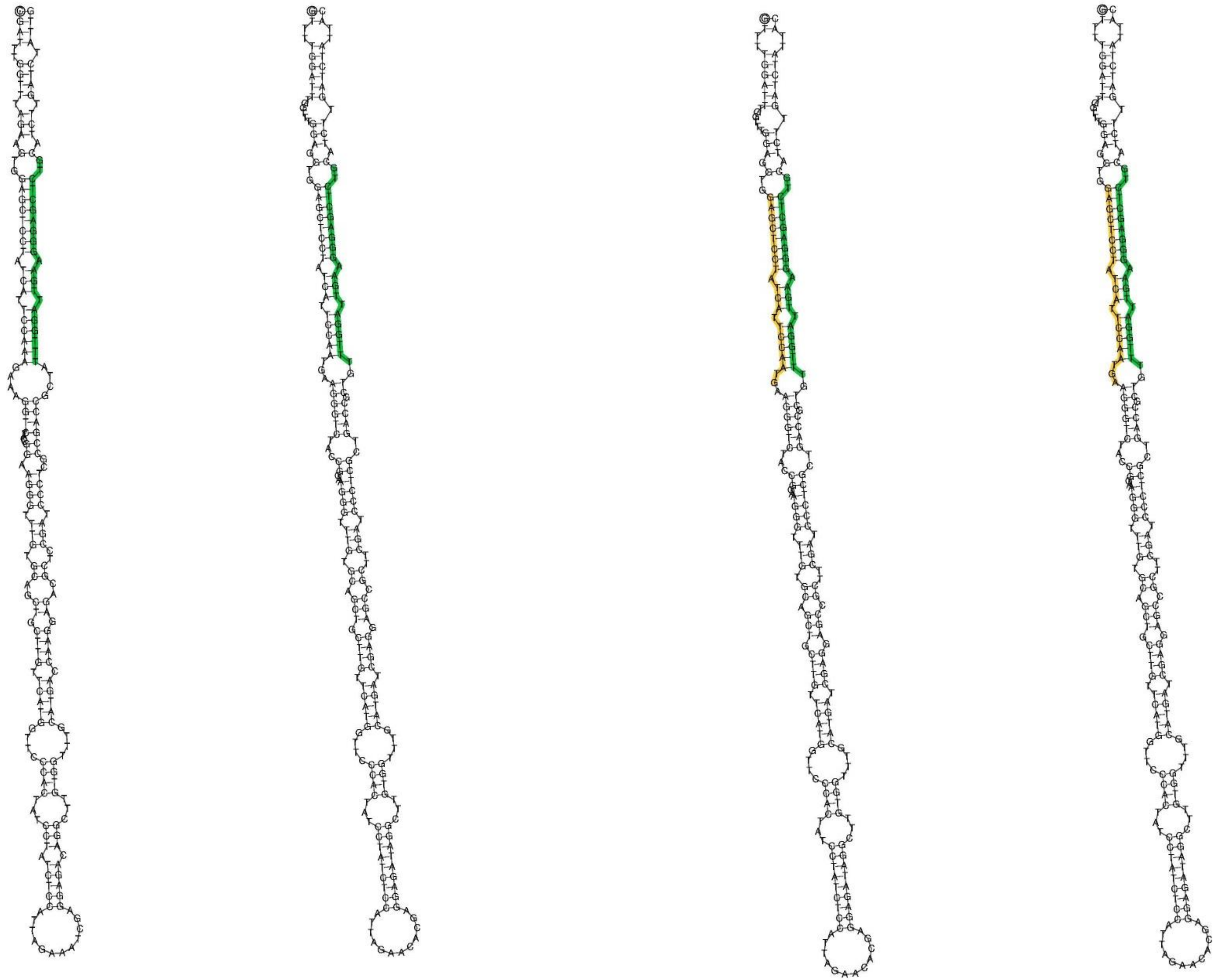

Hvu-new81

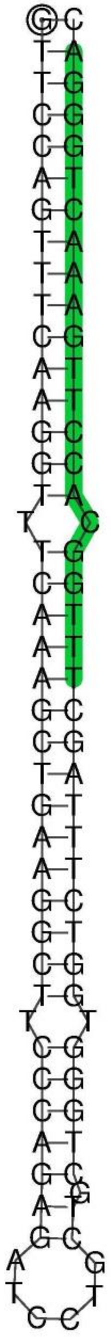

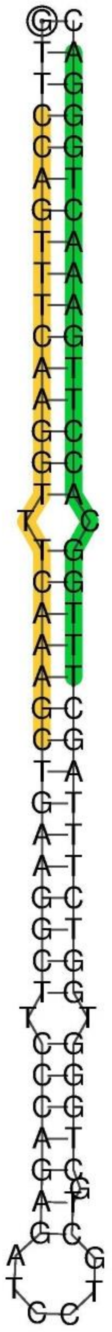

Supplement: Supplementary file 1 [file ijms-22-04315-s001.zip › S1 Figure.pdf]
