# Supplementary material for: Barley Seeds miRNome Stability during Long-Term Storage and Aging
Source: Int J Mol Sci. 2021 Apr 21;22(9):4315. doi: 10.3390/ijms22094315 (PMC8122619; doi:10.3390/ijms22094315)
Supplement: Supplementary file 1 [file ijms-22-04315-s001.zip › S1 Table.pdf]

Table S1. Known miRNAs identified in the tested seed samples [Rc - renewed seeds; Hv - seeds with high viability; Lv - seeds with low viability]

miRNA Sequence	Rc	Lv	Hv
miR1120.1		+	
miR1120.2	+	+	+
miR156a.1			+
miR156a.2		+	
miR156a.3	+	+	+
miR156a.4	+	+	+
miR156a.5	+	+	+
miR156a.6	+	+	+
miR159a.1			+
miR159a.2	+	+	+
miR159a.3	+	+	+
miR159a.4	+	+	+
miR159a.5	+	+	+
miR159a.6	+	+	+
miR166a.1			+
miR166a.2		+	
miR166a.3		+	
miR166a.4	+	+	+
miR166a.5	+	+	+
miR166a.6	+	+	+
miR166a.7	+	+	+
miR168_3p.1		+	+
miR168_3p.2		+	
miR168_3p.3	+	+	+
miR168_3p.4	+	+	
miR168_5p.5		+	
miR168_5p.6			+
miR168_5p.7	+		
miR168_5p.8	+	+	+
miR168_5p.9	+	+	+
miR168_5p.10	+	+	+
miR171_3p.1	+	+	+
miR171_3p.2	+	+	+
miR171_3p.3	+		
miR171_5p.4	+	+	
miR171_5p.5		+	
miR171_5p.6	+	+	+
miR397b_3p.1			+
miR444b.1	+	+	+
miR444b.2		+	
miR444b.3	+	+	+

miR5048a.1	+	+	+
miR5048a.2	+	+	+
miR5048a.3	+	+	+
miR5048a.44	+	+	+
miR5048a.5	+	+	+
miR5048a.6	+		+
miR5048a.7	+	+	+
miR5048a.8	+	+	+
miR5049e.1			+
miR5049e.2	+	+	+
miR5051.1			+
miR5051.2	+	+	
miR5051.3	+	+	+
miR5051.4	+	+	+
miR6200.1			+
miR6200.2			+
miR6201.1			+
miR6201.2	+		+
miR6201.3	+	+	
miR6201.4	+	+	+
