# Supplementary material for: Barley Seeds miRNome Stability during Long-Term Storage and Aging
Source: Int J Mol Sci. 2021 Apr 21;22(9):4315. doi: 10.3390/ijms22094315 (PMC8122619; doi:10.3390/ijms22094315)
Supplement: Supplementary file 1 [file ijms-22-04315-s001.zip › S2 Table.pdf]

Table S2. An average level of expression of known miRNAs identified in the tested seed samples [Rc - renewed seeds; Hv - seeds with high viability; Lv - seeds with low viability]

miRNA	sequence	averaged level of expression [RPM]		
		Rc	Lv	Hv
**miR1120 family**		**22.45**	**45.07**	**59.43**
**miR1120.1**	TTCTTATATTATGGGACGG	0	15.5	0
**miR1120.2**	TTCTTATATTATGGGACGGAG	22.45	29.57	59.43
**miR156 family**		**21539.61**	**15723.15**	**17281.67**
**miR156a.1**	ACAGAAGAGAGTGAGCACA	0	0	11.34
**miR156a.2**	GACAGAAGAGAGTGAGCAC	0	16.01	0
**miR156a.3**	TGACAGAAGAGAGTGAGC	9894.55	7297.93	8551.61
**miR156a.4**	TGACAGAAGAGAGTGAGCA	1524.98	958.67	959.61
**miR156a.5**	TGACAGAAGAGAGTGAGCAC	9740.25	7286.67	7434.18
**miR156a.6**	TGACAGAAGAGAGTGAGCACA	379.83	163.87	324.93
**miR159 family**		**42273.78**	**48560.66**	**40435.63**
**miR159a.1**	TGGATTGAAGGGAGCTCTG	0	0	13.25
**miR159a.2**	TTGGATTGAAGGGAGCTCTG	46.84	31.37	9.56
**miR159a.3**	TTTGGATTGAAGGGAGCT	8042.17	11496.05	9802.06
**miR159a.4**	TTTGGATTGAAGGGAGCTC	2161.7	2497.01	1774.04
**miR159a.5**	TTTGGATTGAAGGGAGCTCT	4390.36	6442.64	5159.53
**miR159a.6**	TTTGGATTGAAGGGAGCTCTG	27632.71	28093.59	23677.19
**miR166 family**		**21086.86**	**22964.34**	**18909.03**
**miR166a.1**	CGGACCAGGCTTCATTCC	0	0	9.7
**miR166a.2**	CGGACCAGGCTTCATTCCCC	0	17.22	0
**miR166a.3**	GGACCAGGCTTCATTCCCC	0	15.42	0
**miR166a.4**	TCGGACCAGGCTTCATTC	1023.67	931.61	840.17
**miR166a.5**	TCGGACCAGGCTTCATTCC	1864.28	1694.53	1850.68
**miR166a.6**	TCGGACCAGGCTTCATTCCC	1342.04	1920.68	1438.32
**miR166a.7**	TCGGACCAGGCTTCATTCCCC	16856.87	18384.88	14770.16
**miR168 family**		**3172,43**	**2088,39**	**2224,99**
**miR168_3p.1**	CCCGCCTTGCACCAAGTG	0	13.16	19.19
**miR168_3p.2**	CCCGCCTTGCACCAAGTGAA	0	13.18	0
**miR168_3p.3**	CCCGCCTTGCACCAAGTGAAT	194.11	85.51	55.21
**miR168_3p.4**	CCGCCTTGCACCAAGTGAAT	14.96	13.15	0
**miR168_5p.5**	CGCTTGGTGCAGATCGGGAC	0	17.55	0
**miR168_5p.6**	CTTGGTGCAGATCGGGAC	0	0	11.15
**miR168_5p.7**	GCTTGGTGCAGATCGGGAC	16.28	0	0
**miR168_5p.8**	TCGCTTGGTGCAGATCGGG	262.67	161.82	189.52
**miR168_5p.9**	TCGCTTGGTGCAGATCGGGA	1049	528.64	616.96
**miR168_5p.10**	TCGCTTGGTGCAGATCGGGAC	1635.41	1255.38	1332.96
**miR171 family**		**118**	**301.89**	**116.16**
**miR171_3p.1**	TGATTGAGCCGTGCCAATA	16.85	41.95	23.28
**miR171_3p.2**	TGATTGAGCCGTGCCAATATC	30.57	135.63	69.6

**miR171_3p.3**	TTGAGCCGTGCCAATATC	14.03	0	0
**miR171_5p.4**	TGTTGGCTCGACTCACTC	15	29.05	0
**miR171_5p.5**	TGTTGGCTCGACTCACTCAG	0	29.17	0
**miR171_5p.6**	TGTTGGCTCGACTCACTCAGA	41.55	66.09	23.28
**miR397 family**		**0**	**0**	**13.25**
**miR397b_3p.1**	ATCAACGCTGCACTCAACGGC	0	0	13.25
**miR444 family**		**70.07**	**76.04**	**51.94**
**miR444b.1**	TGCAGTTGCTGTCTCAAGC	14.24	30.6	19.95
**miR444b.2**	TGCAGTTGCTGTCTCAAGCT	0	15.42	0
**miR444b.3**	TGCAGTTGCTGTCTCAAGCTT	55.83	30.02	31.99
**miR5048 family**		**1739.1**	**1812.47**	**1518.54**
**miR5048a.1**	TATTTGCAGGTTTTAGGT	53.74	154.47	48.46
**miR5048a.2**	TATTTGCAGGTTTTAGGTC	362.74	334.34	380.42
**miR5048a.3**	TATTTGCAGGTTTTAGGTCT	244.76	173.97	222.25
**miR5048a.44**	TATTTGCAGGTTTTAGGTCTA	202.74	92.03	92.4
**miR5048a.5**	TATTTGCAGGTTTTAGGTCTAA	496.67	492.16	392.17
**miR5048a.6**	TTGCAGGTTTTAGGTCTA	8.42	0	9.5
**miR5048a.7**	TTTGCAGGTTTTAGGTCT	105.5	90.18	16.4
**miR5048a.8**	TTTGCAGGTTTTAGGTCTA	264.53	475.32	356.94
**miR5049 family**		**8.46**	**15.9**	**19.39**
**miR5049e.1**	AATTATTTAGGTACAGAGG	0	0	9.7
**miR5049e.2**	AATTATTTAGGTACAGAGGGA	8.46	15.9	9.7
**miR5051 family**		**641.65**	**560.27**	**476.2**
**miR5051.1**	TTTGGCACCTTGAAACTG	0	0	13.25
**miR5051.2**	TTTGGCACCTTGAAACTGG	16.84	13.16	0
**miR5051.3**	TTTGGCACCTTGAAACTGGG	115.21	55.39	42.76
**miR5051.4**	TTTGGCACCTTGAAACTGGGA	509.6	491.72	420.2
**miR6200 family**		**0**	**0**	**21.59**
**miR6200.1**	TTGGCCAACTAGATCTAT	0	0	10.25
**miR6200.2**	TTGGCCAACTAGATCTATG	0	0	11.34
**miR6201 family**		**111.35**	**78.47**	**88.36**
**miR6201.1**	TGACCCTGAGGCACTCATA	0	0	9.56
**miR6201.2**	TGACCCTGAGGCACTCATAC	25.05	0	10.25
**miR6201.3**	TGACCCTGAGGCACTCATACC	15	49.9	0
**miR6201.4**	TGACCCTGAGGCACTCATACCG	71.3	28.57	68.55
