# Supplementary material for: Barley Seeds miRNome Stability during Long-Term Storage and Aging
Source: Int J Mol Sci. 2021 Apr 21;22(9):4315. doi: 10.3390/ijms22094315 (PMC8122619; doi:10.3390/ijms22094315)
Supplement: Supplementary file 1 [file ijms-22-04315-s001.zip › S3 Table.pdf]

Table S3. Novel miRNAs identified in the tested seed samples [Rc - renewed seeds; Hv - seeds with high viability; Lv - seeds with low viability]

Name	Sequence	Rc	Lv	Hv
**hvu-new1**	AAGCTCAGGAGGGATAGCGCC	+		+
**hvu-new2**	AAGTCCTCGTGTTGCATTCCT		+	+
**hvu-new3**	ACACGCGTGATGTGGTCTCTT	+	+	+
**hvu-new4**	ACTGTTGTCGGCCGCGTCGGT	+	+	+
**hvu-new5**	AGTCCTCGTGTTGCATTCCT	+	+	+
**hvu-new6**	AGTGGTGATGAAAATGCTCT	+	+	+
**hvu-new7**	ATGCGGGATGAACCGGAAGT	+	+	
**hvu-new8**	ATGGAGGTAGACGGAAACGGA	+		
**hvu-new9**	ATGGGACTGAAGTTTACCTCT	+	+	
**hvu-new10**	ATGGGACTGAAGTTTACCTCTT	+	+	+
**hvu-new11**	ATTTGTAGTGTTCGGATTGAGT	+	+	+
**hvu-new12**	CAGTTCTTCTGTCCACATCG	+		+
**hvu-new13**	CATGACTCTCGGCAACGGATA	+	+	+
**hvu-new14**	CCCATGTCGCACGGATTCGA	+	+	+
**hvu-new15**	CCTGGGAAGTCCTCGTGTTA	+	+	+
**hvu-new16**	CCTGTTTGTCATTAAATTTCTT	+	+	+
**hvu-new17**	CGAAAGATGGTGAACTATGT		+	+
**hvu-new18**	CGGCAGTGAGGAGAGACAGA	+	+	+
**hvu-new19**	CGGGGTGTGGACTGTTGTCA	+	+	+
**hvu-new20**	CTCGCGAGCAACGGATGAATC	+	+	+
**hvu-new21**	CTGGCAACAAATTAGAGTCTC			+
**hvu-new22**	CTGGCAACAAATTAGAGTCTCT	+	+	
**hvu-new23**	CTGTTGAGCTTGACTCTAGTCT	+	+	+
**hvu-new24**	CTTCCCAACGGGCGGTGGGCTA			+
**hvu-new25**	GACTGTTGTCGGCCGCGTCA	+		+
**hvu-new26**	GAGGGCGGCGATAACATTTTC	+	+	+
**hvu-new27**	GCCAGGACTGTAACCATGTGT	+		
**hvu-new28**	GCCGACCTAGCTCAGTGGTA	+	+	+
**hvu-new29**	GCGGAACGGCTCTTGCTGGTT	+	+	
**hvu-new30**	GGTGTGGACTGTTGTCGGCG	+	+	+
**hvu-new31**	GTTGCCGTCGTCGACATGGT	+	+	+
**hvu-new32**	TAAGGACTTAAAGTGGGCATT	+		
**hvu-new33**	TAATAAGCATGTCTTCAGATG	+	+	+
**hvu-new34**	TAATGATCTTATCTCGGTGTTG	+		
**hvu-new35**	TATAAGTCTTTGTAGAGATC	+		+
**hvu-new36**	TATTGGCTCGGCTCACTCAGG	+	+	+
**hvu-new37**	TCAAGTGATGAGGAATAAACT	+	+	+
**hvu-new38**	TCAGACTTCGCTGGGACATC	+	+	+

**hvu-new39**	TCAGTGCGATCCCTCTGGAAT	+	+	+
**hvu-new40**	TCCACAGGCTTTCTTGAACTG	+	+	+
**hvu-new41**	TCCCAACGGGCGGTGGGCTA	+	+	
**hvu-new42**	TCCGTTGTAGTCTAGGTGGTT	+	+	+
**hvu-new43**	TCGCTTGGTGCAGATCGGGAC	+	+	+
**hvu-new44**	TCGGACCAGGCTTCAATCCCT	+	+	+
**hvu-new45**	TCGGACCAGGCTTCATTCCCC	+	+	+
**hvu-new46**	TCGGACCAGGCTTCATTCCCT	+	+	+
**hvu-new47**	TCTGGATGTTGATTAGATGTT	+		+
**hvu-new48**	TCTTATGTTGTGGGACGGAGG	+		+
**hvu-new49**	TGAAGCTGCCAGCATGATCT	+		+
**hvu-new50**	TGAAGCTGCCAGCATGATCTA	+	+	+
**hvu-new51**	TGAAGCTGCCAGCATGATCTG	+	+	+
**hvu-new52**	TGAAGCTGCCAGCATGATCTGA	+	+	+
**hvu-new53**	TGAAGCTGCCAGCATGATCTGC		+	+
**hvu-new54**	TGACAGAAGAGAGTGAGCAC	+	+	+
**hvu-new55**	TGAGCTTGACTCTAGTCCGACC	+	+	+
**hvu-new56**	TGAGGAAGGACTGCATCATCT	+	+	+
**hvu-new57**	TGCAGCGCCGGTGAACCGCTCC		+	+
**hvu-new58**	TGCGGTGATGAATTATGAATT	+	+	+
**hvu-new59**	TGCTGTGATGAATCGCAAGGAC	+	+	+
**hvu-new60**	TGGAAGGGGCATGCAGAGGAG	+		
**hvu-new61**	TGGACGTGGAGGTGCAGCTGC	+		
**hvu-new62**	TGGACTGAAGGGAGCTCCCTC	+	+	+
**hvu-new63**	TGGAGAAGCAGGGCACGTGCT		+	+
**hvu-new64**	TGGAGGTAGACGGAAACGGAG	+		+
**hvu-new65**	TGGCCTGTCAAGTACGCGTGC	+		
**hvu-new66**	TTAAATTTCTCCATAGCATCA	+	+	+
**hvu-new67**	TTACGGATGTAGTATCATACT	+		+
**hvu-new68**	TTCATCGGTGAGTAAGGTCC	+		+
**hvu-new69**	TTCCACAGCTTTCTTGAACTG	+	+	+
**hvu-new70**	TTGAACTGTTTCCTCTGAAAT	+	+	+
**hvu-new71**	TTGAGCCGTGCCAATATCTCT	+	+	+
**hvu-new72**	TTGAGGAAGGACTGCATCATC	+		
**hvu-new73**	TTGAGTGCAGCGTTGATGAAC	+		
**hvu-new74**	TTGATTTCGCGGCTCATCTTGC	+	+	+
**hvu-new75**	TTGGACGTGGAGGTGCAGCTG	+	+	+
**hvu-new76**	TTGGCATTCTGTCCACCTCC	+	+	+
**hvu-new77**	TTGTCTAGAAATGAATGTATTT	+	+	
**hvu-new78**	TTGTCTAGATATGAATGTTC	+	+	
**hvu-new79**	TTGTCTAGATATGAATGTTT	+	+	+

**hvu-new80**	TTTGGATTGAAGGGAGCTCTG	+	+	+
**hvu-new81**	TTTGGCACCTTGAAACTGGGA	+	+	+
