# Supplementary material for: Barley Seeds miRNome Stability during Long-Term Storage and Aging
Source: Int J Mol Sci. 2021 Apr 21;22(9):4315. doi: 10.3390/ijms22094315 (PMC8122619; doi:10.3390/ijms22094315)
Supplement: Supplementary file 1 [file ijms-22-04315-s001.zip › S4 Table.pdf]

Table S4. An average level of expression of novel miRNAs identified in the tested seed samples [Rc - renewed seeds; Hv - seeds with high viability; Lv - seeds with low viability]

name	mature sequence miRNA	averaged level of expression RPM		
		Rc	Hv	Lv
hvu-new1	AAGCTCAGGAGGGATAGCGCC	0.09	0.06	0.00
hvu-new2	AAGTCCTCGTGTTGCATTCCT	0.00	2.00	3.43
hvu-new3	ACACGCGTGATGTGGTCTCTT	0.48	0.12	0.50
hvu-new4	ACTGTTGTCGGCCGCGTCGGT	0.98	0.99	1.17
hvu-new5	AGTCCTCGTGTTGCATTCCT	3.70	1.80	0.76
hvu-new6	AGTGGTGATGAAAATGCTCT	0.41	0.16	0.73
hvu-new7	ATGCGGGATGAACCGGAAGT	0.14	0.00	0.05
hvu-new8	ATGGAGGTAGACGGAAACGGA	0.11	0.00	0.00
hvu-new9	ATGGGACTGAAGTTTACCTCT	0.05	0.00	0.08
hvu-new10	ATGGGACTGAAGTTTACCTCTT	0.04	0.04	0.18
hvu-new11	ATTTGTAGTGTTCGGATTGAGT	0.14	0.16	0.21
hvu-new12	CAGTTCTTCTGTCCACATCG	0.03	0.06	0.00
hvu-new13	CATGACTCTCGGCAACGGATA	0.10	0.31	0.39
hvu-new14	CCCATGTCGCACGGATTCGA	3.98	2.36	3.15
hvu-new15	CCTGGGAAGTCCTCGTGTTA	0.05	0.12	0.09
hvu-new16	CCTGTTTGTCATTAAATTTCTT	0.09	0.05	0.05
hvu-new17	CGAAAGATGGTGAACTATGT	0.00	0.18	0.24
hvu-new18	CGGCAGTGAGGAGAGACAGA	0.09	0.06	0.07
hvu-new19	CGGGGTGTGGACTGTTGTCA	0.27	0.80	0.26
hvu-new20	CTCGCGAGCAACGGATGAATC	0.07	0.08	0.26
hvu-new21	CTGGCAACAAATTAGAGTCTC	0.00	0.05	0.00
hvu-new22	CTGGCAACAAATTAGAGTCTCT	0.06	0.00	0.30
hvu-new23	CTGTTGAGCTTGACTCTAGTCT	1.90	1.12	1.88
hvu-new24	CTTCCCAACGGGCGGTGGGCTA	0.00	0.16	0.00
hvu-new25	GACTGTTGTCGGCCGCGTCA	0.04	0.10	0.00
hvu-new26	GAGGGCGGCGATAACATTTTC	0.28	0.29	0.09
hvu-new27	GCCAGGACTGTAACCATGTGT	0.03	0.00	0.00
hvu-new28	GCCGACCTAGCTCAGTGGTA	1.24	1.16	0.60
hvu-new29	GCGGAACGGCTCTTGCTGGTT	0.14	0.00	0.12
hvu-new30	GGTGTGGACTGTTGTCGGCG	0.19	0.29	0.16
hvu-new31	GTTGCCGTCGTCGACATGGT	0.19	0.16	0.09
hvu-new32	TAAGGACTTAAAGTGGGCATT	0.05	0.00	0.00
hvu-new33	TAATAAGCATGTCTTCAGATG	0.63	0.50	0.47
hvu-new34	TAATGATCTTATCTCGGTGTTG	0.52	0.00	0.00
hvu-new35	TATAAGTCTTTGTAGAGATC	0.06	0.11	0.00
hvu-new36	TATTGGCTCGGCTCACTCAGG	0.19	0.09	0.04
hvu-new37	TCAAGTGATGAGGAATAAACT	0.29	0.30	0.18
hvu-new38	TCAGACTTCGCTGGGACATC	0.95	0.40	0.51
hvu-new39	TCAGTGCGATCCCTCTGGAAT	0.17	0.04	0.05

**hvu-new40**	TCCACAGGCTTTCTTGAACTG	8.79	7.06	5.77
**hvu-new41**	TCCCAACGGGCGGTGGGCTA	0.04	0.00	0.14
**hvu-new42**	TCCGTTGTAGTCTAGGTGGTT	0.11	0.07	0.04
**hvu-new43**	TCGCTTGGTGCAGATCGGGAC	1.83	1.34	0.96
**hvu-new44**	TCGGACCAGGCTTCAATCCCT	2.76	1.79	1.91
**hvu-new45**	TCGGACCAGGCTTCATTCCCC	74.93	63.18	62.37
**hvu-new46**	TCGGACCAGGCTTCATTCCCT	7.29	5.45	5.73
**hvu-new47**	TCTGGATGTTGATTAGATGTT	0.17	0.03	0.00
**hvu-new48**	TCTTATGTTGTGGGACGGAGG	0.32	0.10	0.00
**hvu-new49**	TGAAGCTGCCAGCATGATCT	1.50	0.40	0.00
**hvu-new50**	TGAAGCTGCCAGCATGATCTA	0.59	0.46	0.90
**hvu-new51**	TGAAGCTGCCAGCATGATCTG	0.83	0.48	0.21
**hvu-new52**	TGAAGCTGCCAGCATGATCTGA	13.83	11.33	12.29
**hvu-new53**	TGAAGCTGCCAGCATGATCTGC	0.00	0.04	0.13
**hvu-new54**	TGACAGAAGAGAGTGAGCAC	43.49	31.99	25.08
**hvu-new55**	TGAGCTTGACTCTAGTCCGACC	0.15	0.12	0.09
**hvu-new56**	TGAGGAAGGACTGCATCATCT	0.05	0.13	0.10
**hvu-new57**	TGCAGCGCCGGTGAACCGCTCC	0.00	0.11	0.08
**hvu-new58**	TGCGGTGATGAATTATGAATT	0.10	0.18	0.12
**hvu-new59**	TGCTGTGATGAATCGCAAGGAC	0.03	0.04	0.10
**hvu-new60**	TGGAAGGGGCATGCAGAGGAG	0.66	0.00	0.00
**hvu-new61**	TGGACGTGGAGGTGCAGCTGC	0.20	0.00	0.00
**hvu-new62**	TGGACTGAAGGGAGCTCCCTC	7.18	7.32	5.93
**hvu-new63**	TGGAGAAGCAGGGCACGTGCT	0.00	0.07	0.04
**hvu-new64**	TGGAGGTAGACGGAAACGGAG	0.31	0.18	0.00
**hvu-new65**	TGGCCTGTCAAGTACGCGTGC	0.07	0.00	0.00
**hvu-new66**	TTAAATTTCTCCATAGCATCA	0.25	0.58	0.75
**hvu-new67**	TTACGGATGTAGTATCATACT	0.03	0.06	0.00
**hvu-new68**	TTCATCGGTGAGTAAGGTCC	0.06	0.08	0.00
**hvu-new69**	TTCCACAGCTTTCTTGAACTG	0.60	0.08	0.42
**hvu-new70**	TTGAACTGTTTCCTCTGAAAT	0.09	0.07	0.08
**hvu-new71**	TTGAGCCGTGCCAATATCTCT	0.10	0.06	0.03
**hvu-new72**	TTGAGGAAGGACTGCATCATC	0.05	0.00	0.00
**hvu-new73**	TTGAGTGCAGCGTTGATGAAC	0.62	0.00	0.00
**hvu-new74**	TTGATTTCGCGGCTCATCTTGC	26.79	16.75	15.93
**hvu-new75**	TTGGACGTGGAGGTGCAGCTG	0.10	0.06	0.03
**hvu-new76**	TTGGCATTCTGTCCACCTCC	0.60	0.40	0.38
**hvu-new77**	TTGTCTAGAAATGAATGTATTT	0.05	0.00	0.09
**hvu-new78**	TTGTCTAGATATGAATGTTC	0.04	0.00	0.07
**hvu-new79**	TTGTCTAGATATGAATGTTT	0.43	0.30	0.18
**hvu-new80**	TTTGGATTGAAGGGAGCTCTG	124.76	103.36	97.81
**hvu-new81**	TTTGGCACCTTGAAACTGGGA	0.60	0.37	0.37
