# Supplementary material for: Barley Seeds miRNome Stability during Long-Term Storage and Aging
Source: Int J Mol Sci. 2021 Apr 21;22(9):4315. doi: 10.3390/ijms22094315 (PMC8122619; doi:10.3390/ijms22094315)
Supplement: Supplementary file 1 [file ijms-22-04315-s001.zip › S9 Table.pdf]

Table S9. Primers used in the RT-qPCR of miRNA

Name of primers	Sequence 5'->3'	Tm [°C]
**Reference miRNA**		
**mir159**	TTTGGATTGAAGGGAGCTCTG	
**miR159_R3**	GGTCCAGTTTTTTTTTTTTTTTCAG	49.5
**miR159_F2**	GCGCAGTTTGGATTGAAG	48.0
**mir166**	TCGGACCAGGCTTCATTCCCC	
**miR166_R1**	CAGTTTTTTTTTTTTTTTGGGGAATG	50.1
**miR166_F4**	CAGTCGGACCAGGCT	47.4
**mir168**	CCCGCCTTGCACCAAGTGAAT	
**miR168_R1**	GGTCCAGTTTTTTTTTTTTTTTATTCACT	51.6
**miR168_F3**	CCCGCCTTGCACCA	46.2
**Explored miRNA**		
**hvu-new2_R_1**	GTCCAGTTTTTTTTTTTTTTTTAGGAATG	51.1
**hvu-new2_F_2**	GCAGAAGTCCTCGTGTTG	50.3
**hvu-new41_R_1**	GGTCCAGTTTTTTTTTTTTTTTAGC	49.5
**hvu-new41_F_1**	CCCAACGGGCGGT	46.0
**hvu-new48_R_1**	GTCCAGTTTTTTTTTTTTTTTCCTC	49.5
**hvu-new48_F_2**	AGCGCAGTCTTATGTTGTG	48.9
**hvu-new60_R_1**	TCCAGTTTTTTTTTTTTTTTCTCCTC	50.1
**hvu-new60_F_3**	GGAAGGGGCATGCAG	47.4
**hvu-new69_R_2**	GTCCAGTTTTTTTTTTTTTTTCAGTTC	50.6
**hvu-new69_F_2**	GCAGTTCCACAGCTTTCTTG	51.8
**miR156*_R_1**	TCCAGTTTTTTTTTTTTTTTGTGCT	47.8
**miR156*_F_2**	GCAGTGACAGAAGAGAGTGA	51.8
**miR171e_R_1**	CCAGTTTTTTTTTTTTTTTATTGGCA	48.5
**miR171e_F_2**	GCAGTGATTGAGCCGT	45.9
**miR171d_R_3**	AGGTCCAGTTTTTTTTTTTTTTTGAT	48.5
**miR171d_F_1**	TGATTGAGCCGTGCCA	45.9
**miR171c_R_1**	TCCAGTTTTTTTTTTTTTTTGAGTGA	48.5
**miR171c_F_4**	GCAGTGTTGGCTCGAC	48.5
**miR171b_R_4**	GGTCCAGTTTTTTTTTTTTTTTCTG	49.5
**miR171b_F_2**	GTGTTGGCTCGACTCAC	49.5
